# Healthy dietary patterns are associated with exposure to environmental chemicals in a pregnancy cohort

**DOI:** 10.1038/s43016-024-01013-x

**Published:** 2024-07-01

**Authors:** Guoqi Yu, Ruijin Lu, Jiaxi Yang, Mohammad L. Rahman, Ling-Jun Li, Dong D. Wang, Qi Sun, Wei Wei Pang, Claire Guivarch, Anna Birukov, Jagteshwar Grewal, Zhen Chen, Cuilin Zhang

**Affiliations:** 1 Global Centre for Asian Women’s Health, Yong Loo Lin School of Medicine, National University of Singapore, Singapore, Singapore; 2 Department of Obstetrics and Gynaecology, Yong Loo Lin School of Medicine, National University of Singapore, Singapore, Singapore; 3 Bia-Echo Asia Centre for Reproductive Longevity and Equality, Yong Loo Lin School of Medicine, National University of Singapore, Singapore, Singapore; 4 Division of Biostatistics, School of Medicine, Washington University in St. Louis, St. Louis, MO USA; 5 Occupational and Environmental Epidemiology Branch, Division of Cancer Epidemiology and Genetics, National Cancer Institute, Rockville, MD USA; 6 Channing Division of Network Medicine, Harvard Medical School and Brigham and Women’s Hospital and Department of Nutrition, Harvard T. H. Chan School of Public Health, Boston, MA USA; 7 Department of Nutrition, Harvard T. H. Chan School of Public Health, Boston, MA USA; 8 Division of Population Health Research, Eunice Kennedy Shriver National Institute of Child Health and Human Development, National Institutes of Health, Bethesda, MD USA

**Keywords:** Environmental impact, Epidemiology

## Abstract

Healthy dietary patterns, such as the alternate Mediterranean diet and alternate Healthy Eating Index, benefit cardiometabolic health. However, several food components of these dietary patterns are primary sources of environmental chemicals. Here, using data from a racially and ethnically diverse US cohort, we show that healthy dietary pattern scores were positively associated with plasma chemical exposure in pregnancy, particularly for the alternate Mediterranean diet and alternate Healthy Eating Index with polychlorinated biphenyls and per- and poly-fluoroalkyl substances. The associations appeared stronger among Asian and Pacific Islanders. These findings suggest that optimizing the benefits of a healthy diet requires concerted regulatory efforts aimed at lowering environmental chemical exposure.

## Abstract (web summary)

Data on chemical concentrations in commonly recommended diets are sparse. This study estimates the association between adherence to three dietary patterns considered healthy and exposure to environmental chemicals in a multi-racial pregnancy cohort, underscoring the need for increased regulation and monitoring of environmental contaminants.

## Main

Current dietary guidelines endorse higher adherence to healthy dietary patterns, such as the alternate Mediterranean diet (aMED), alternate Healthy Eating Index (aHEI) and Dietary Approaches to Stop Hypertension (DASH) over specific, individual foods or nutrients as people usually do not eat single foods^1,2^. Accumulating evidence demonstrates that adherence to aMED, aHEI and DASH is linked to reduced risks for cardiometabolic diseases and pregnancy complications^3^.

Several major food components of the three healthy dietary patterns, however, are sources of some environmental chemicals. For example, per- and poly-fluoroalkyl substances (PFAS), heavy metals and polychlorinated biphenyls (PCBs) can be traced to seafood consumption due to bioaccumulation^4^. Despite efforts to reduce their release, many of these chemicals persist in the environment and permeate the human body via diverse pathways, especially through the food chain^5^. The majority of these chemical exposure have been associated with diverse health outcomes, particularly for pregnant women and foetuses who are sensitive to environmental stimuli owing to intensive metabolic disturbance, incomplete immune protection and rapid cell division during early embryonic development^6^.

As such, it is pivotal to understand the environmental-chemical portfolio of the commonly recommended healthy dietary patterns. Such data are sparse so far, with most previous studies focusing on individual food items, overlooking the synergic effects of major food groups and nutrients on circulating chemicals^7^.

Elucidating the associations between healthy dietary patterns and blood concentrations of environmental chemicals has the potential to unveil the degree to which these healthy dietary patterns may be related to potentially ‘harmful’ chemical exposures. Such findings may raise caution in monitoring chemical exposure introduced by specific food consumption that is linked to healthy dietary patterns, and enrich dietary recommendations by promoting a healthy diet while minimizing the intake of toxic chemicals. In a large racially and ethnically diverse pregnancy cohort, we aimed to examine associations of three dietary patterns with a comprehensive panel of environmental chemicals and evaluate whether such associations could be attributed to the consumption of specific food groups.

## Results

Food groups and nutrients that constitute the three dietary patterns and the amounts consumed are listed in Supplementary Table 1. The characteristics of study participants are presented in Supplementary Table 2. Plasma concentration differences of chemicals according to aMED, aHEI and DASH scores (high versus low, dichotomized by median) are presented in Table 1 and Supplementary Table 3. Differences in the levels of chemicals between participants with high versus low dietary pattern scores were observed. Specifically, compared with those with lower scores, participants with higher aMED or aHEI scores had higher concentrations of perfluorododecanoic acid (PFDoDA), perfluorononanoic acid (PFNA), perfluorodecanoic acid (PFDA), perfluoroundecanoic acid (PFUnDA), total PFASs, caesium (Cs), mercury (Hg), molybdenum (Mo) and thallium (Tl) and lower concentrations of *N*-methylperfluoro-1-octanesulfonamidoacetic acid (NMeFOSAA), copper (Cu), zinc (Zn) and total metals. Higher scores of healthy dietary patterns tended to be linked to higher organochlorine pesticide (OCP) concentrations. On the contrary, participants with high dietary pattern scores tended to have low concentrations of total polybrominated diphenyl ethers (PBDEs), BDE47, BDE100, BDE99 and BDE183.

**Table 1:** Plasma concentration differences of chemicals according to aMED, aHEI and DASH scores among the NICHD Fetal Growth Study–Singletons cohort

	aMED			aHEI			DASH		
Chemical	Low aMED	High aMED	Adjusted *P* value	Low aHEI	High aHEI	Adjusted *P* value	Low DASH	High DASH	Adjusted *P* value
BetaHCH	0.74 (2.36)	1.21 (4.57)	**	0.59 (1.94)	1.51 (5.77)	***	0.89 (3.08)	1.28 (4.64)	
HCB	5.56 (9.68)	6.59 (7.42)	*	6.1 (9.64)	6.48 (7.53)	*	6.2 (9.18)	6.56 (6.67)	
TransNo_chlor	3.98 (4.44)	4.56 (5.46)	*	4.18 (4.85)	4.47 (5.16)		4.26 (4.92)	4.54 (4.97)	
P_P_DDE	66.59 (86.63)	83.22 (123.08)	***	63.59 (82.78)	88.78 (135.1)	***	71.71 (103.49)	85.4 (122.59)	*
P_P_DDD	0.31 (0.14)	0.32 (0.29)	*	0.32 (0.14)	0.32 (0.31)		0.32 (0.24)	0.32 (0.22)	
P_P_DDT	0.89 (1.8)	1.37 (2.64)	***	0.83 (1.59)	1.51 (3.05)	***	1.02 (2.18)	1.46 (2.61)	*
Mirex	0.32 (0.32)	0.33 (0.57)		0.32 (0.23)	0.34 (0.7)	***	0.32 (0.38)	0.33 (0.62)	
Total OCPs	90.92 (109.14)	109.92 (139.99)	***	87.41 (100.86)	118.54 (155.67)	***	98.55 (121.66)	110.06 (142.71)	*
BDE47	9.65 (14.3)	8.2 (13.28)	*	9.78 (14.46)	7.6 (12.34)	***	9.27 (14.56)	7.48 (11.12)	***
BDE100	2.86 (4.21)	2.3 (4.15)	**	2.84 (3.98)	2.12 (4.19)	***	2.72 (4.29)	2.02 (3.8)	***
BDE99	2.47 (4.89)	2.1 (4.37)		2.55 (5.2)	1.98 (4.33)	*	2.42 (5.12)	1.83 (3.59)	**
BDE183	0.31 (0.1)	0.3 (0.07)	*	0.31 (0.09)	0.3 (0.08)	*	0.31 (0.09)	0.3 (0.07)	*
Total PBDEs	26.68 (34.86)	21.53 (31.2)	***	25.71 (34.19)	21.19 (31.09)	***	25.34 (35.18)	18.53 (29.22)	***
PCB74_61	0.93 (1.08)	1.14 (1.33)	***	0.9 (0.94)	1.23 (1.48)	***	1.01 (1.14)	1.19 (1.35)	*
PCB66_80	0.61 (0.18)	0.63 (0.2)		0.61 (0.16)	0.63 (0.22)	*	0.62 (0.19)	0.62 (0.2)	
PCB99	0.91 (1.08)	1.12 (1.4)	***	0.92 (1.05)	1.15 (1.5)	***	1.06 (1.19)	1.07 (1.33)	
PCB118_106	1.6 (2.04)	2.05 (2.38)	***	1.58 (1.99)	2.13 (2.55)	***	1.84 (2.26)	2 (2.46)	
PCB105_127	0.65 (0.31)	0.69 (0.57)	***	0.65 (0.31)	0.71 (0.65)	***	0.67 (0.41)	0.69 (0.56)	
PCB146_161	0.63 (0.25)	0.69 (0.66)	***	0.64 (0.26)	0.72 (0.77)	***	0.66 (0.48)	0.68 (0.64)	
PCB153	4.43 (5.03)	6.09 (7.59)	***	4.43 (4.97)	6.63 (8.26)	***	5.15 (6.24)	6.1 (7.79)	***
PCB138_158	3.76 (4.15)	4.91 (6.06)	***	3.78 (4.07)	5.33 (6.19)	***	4.19 (4.85)	5.14 (6.22)	***
PCB156	0.63 (0.23)	0.67 (0.53)	***	0.64 (0.23)	0.68 (0.63)	***	0.65 (0.32)	0.69 (0.65)	**
PCB182_187	0.92 (1.21)	1.35 (1.82)	***	0.92 (1.15)	1.44 (1.95)	***	1.12 (1.54)	1.31 (1.78)	**
PCB183	0.61 (0.17)	0.65 (0.3)	***	0.62 (0.18)	0.65 (0.33)	***	0.63 (0.22)	0.64 (0.28)	
PCB177	0.6 (0.14)	0.61 (0.15)	*	0.6 (0.14)	0.6 (0.16)		0.61 (0.15)	0.6 (0.15)	
PCB180	2.54 (3)	3.69 (4.3)	***	2.43 (2.75)	4.02 (4.51)	***	2.87 (3.44)	3.99 (4.32)	***
PCB170	1.07 (1.1)	1.47 (1.68)	***	0.99 (1.07)	1.59 (1.79)	***	1.17 (1.33)	1.56 (1.77)	***
PCB199	0.64 (0.32)	0.71 (0.68)	***	0.65 (0.29)	0.73 (0.8)	***	0.67 (0.44)	0.71 (0.67)	
PCB196_203	0.67 (0.41)	0.76 (0.75)	***	0.67 (0.42)	0.78 (0.83)	***	0.69 (0.54)	0.77 (0.79)	*
PCB194	0.64 (0.26)	0.71 (0.58)	***	0.64 (0.25)	0.74 (0.64)	***	0.66 (0.36)	0.75 (0.63)	***
PCB206	0.6 (0.16)	0.62 (0.2)	***	0.61 (0.16)	0.62 (0.2)	*	0.61 (0.16)	0.62 (0.21)	
PCB209	0.59 (0.14)	0.61 (0.14)	*	0.6 (0.14)	0.6 (0.15)		0.6 (0.14)	0.59 (0.14)	
Total PCBs	21.21 (30.1)	29.96 (40.2)	***	20.86 (29.59)	32.9 (42.13)	***	25.33 (35.73)	29.95 (39)	**
NMeFOSAA	0.06 (0.09)	0.05 (0.08)	**	0.06 (0.1)	0.04 (0.08)	***	0.06 (0.09)	0.04 (0.08)	***
PFDS	0.04 (0)	0.04 (0)		0.04 (0)	0.04 (0)	**	0.04 (0)	0.04 (0)	
PFDoDA	0.02 (0.02)	0.03 (0.04)	***	0.02 (0.02)	0.03 (0.04)	***	0.02 (0.03)	0.02 (0.03)	
PFOS	4.48 (3.67)	4.84 (4.19)	*	4.69 (3.61)	4.81 (4.4)		4.8 (3.89)	4.66 (4.26)	
PFOA	1.73 (1.42)	1.81 (1.4)	*	1.72 (1.26)	1.89 (1.49)	***	1.73 (1.3)	1.92 (1.6)	***
PFNA	0.71 (0.52)	0.78 (0.63)	**	0.7 (0.49)	0.82 (0.64)	***	0.73 (0.57)	0.77 (0.58)	
PFDA	0.2 (0.19)	0.24 (0.27)	***	0.2 (0.19)	0.25 (0.29)	***	0.22 (0.23)	0.24 (0.25)	
PFUnDA	0.13 (0.16)	0.2 (0.28)	***	0.13 (0.17)	0.22 (0.32)	***	0.16 (0.24)	0.18 (0.22)	
Total PFASs	8.8 (6.66)	9.37 (6.92)	*	8.86 (5.63)	9.55 (7.69)	**	9.19 (6.33)	9.07 (7.37)	
As	0.49 (0)	0.49 (0)	***	0.49 (0)	0.49 (0)	***	0.49 (0)	0.49 (0)	
Cs	0.34 (0.18)	0.39 (0.19)	***	0.33 (0.16)	0.42 (0.2)	***	0.35 (0.19)	0.42 (0.19)	***
Cu	1,895 (463)	1,875 (464)		1,903 (454)	1,855 (460)	*	1,894 (475)	1,848 (453)	
Hg	0.19 (0.17)	0.19 (0.26)	***	0.19 (0.14)	0.29 (0.33)	***	0.19 (0.22)	0.19 (0.26)	
Mo	1.74 (1.18)	2.01 (1.3)	^***^	1.77 (1.16)	2.02 (1.28)	***	1.84 (1.21)	2.04 (1.28)	**
Tl	0.02 (0.01)	0.02 (0.02)	^*^	0.02 (0.01)	0.03 (0.02)	*	0.02 (0.01)	0.02 (0.02)	
Zn	817 (201)	776 (182)	^***^	813 (191)	775 (184)	***	810 (191)	753 (176)	***
Total metals	2,827 (533)	2,784 (523)	^*^	2,823 (512)	2,773 (545)	*	2,821 (520)	2,739 (539)	***

Significant chemicals are presented, while the complete table can be found in Supplementary Table 3. Group differences of chemicals (high versus low, dichotomized by the median of each dietary pattern score) were examined by a non-parametric test. To account for multiple comparisons, Benjamini–Hochberg (BH)-adjusted and two-sided *P* values were calculated. *P < 0.05, **P < 0.01, ***P < 0.001. Chemicals (ng g^−1^ lipid, except for PFASs and metals, ng ml^−1^) were standardized by total lipids.

**Fig. 1:**
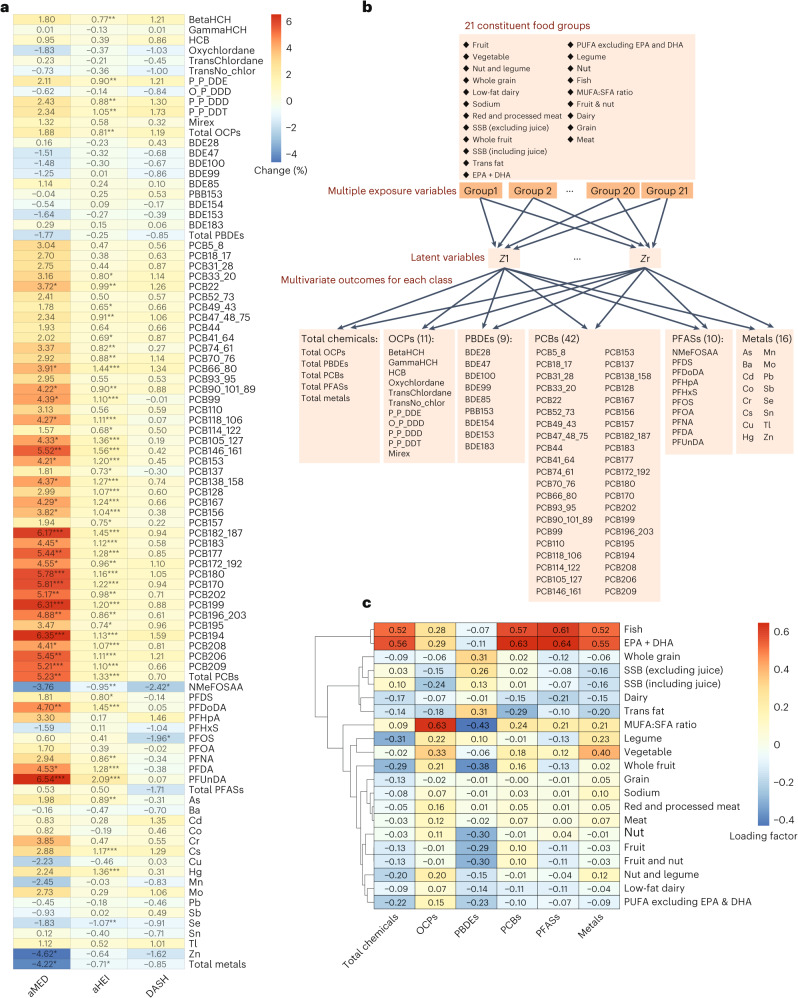
Associations of different dietary patterns with chemicals among the NICHD Fetal Growth Study–Singleton cohort. **a**, Per cent difference in grouped and individual plasma chemical concentrations per 1 s.d. increase in dietary pattern indices of aHEI, aMED and DASH. All estimations were assessed by multivariable linear regression models with adjustment for maternal race/ethnicity, age, physical activity level, pre-pregnancy body mass index (BMI), education level, income, parity, tobacco exposure and total energy intake. Significance with two-sided raw *P* value <0.05 is bolded. To account for multiple comparisons, Benjamini–Hochberg (BH)-adjusted *P* values were calculated. *P < 0.001, **P < 0.01, ***P < 0.05. Per cent change ((exp(*β*) − 1) × 100) was reported to benefit interpretation. **b**, Conceptual diagram of the Kernal RRR. The black arrows represent the dependency structure. **c**, The loading effect of different food groups and components on chemical classes with residuals of the above confounders adjusted, which can help describe the strength and directionality of how the intake of each food group is loaded onto a specific dietary pattern with different chemical classes. SSB, sugar-sweetened beverage; PUFA, polyunsaturated fatty acid.

Overall, we observed significant and positive associations of the three healthy dietary pattern scores with plasma concentrations of PCBs, with notable associations observed for aMED and aHEI with PCBs. Specifically, aMED and aHEI were positively associated with total PCBs, with per cent change (95% CI) of 5.2 (2.5–8.0) and 1.3 (0.8–1.8), respectively (Fig. 1a and Supplementary Table 4). Positive associations with aMED and aHEI were observed for the majority of individual PCB congeners. Adherence to aMED was positively associated with PFDoDA, PFDA and PFUnDA. Adherence to aHEI was positively associated with perfluorodecane sulfonate (PFDS), PFDoDA, PFNA, PFDA and PFUnDA but inversely associated with NMeFOSAA. aHEI score was positively associated with β-hexachlorocyclohexane (BetaHCH), *p*,*p*′-dichlorodiphenyldichloroethylene (P_P_DDE), *p*,*p*′-dichlorodiphenyldichloroethane (P_P_DDD), *p*,*p*′-dichlorodiphenyltrichloroethane (P_P_DDT) and total OCPs as well. The DASH score was inversely associated with NMeFOSAA and perfluorooctanesulfonic acid (PFOS). The aMED was inversely associated with Zn and total metals; the aHEI score was positively associated with As, Cs and Hg but inversely associated with selenium (Se).

Reduced rank regression (RRR) analyses indicated that fish (0.52) and eicosapentaenoic acid (EPA) + docosahexaenoic acid (DHA) (0.56) were the highest loading factors for the variation in total chemicals (Fig. 1c). Loading factors of similar food groups/nutrients were observed for variations in PCBs and PFASs, with 0.57 and 0.61 for fish and 0.63 and 0.64 for EPA + DHA, respectively. The ratio of monounsaturated fatty acids (MUFAs) to saturated fatty acids (SFAs) (0.63) and vegetables (0.33) were high loading factors for OCPs. Fish (0.52), EPA + DHA (0.55) and vegetable (0.40) contributed positively to the variation in metals. Consistent findings were observed in multivariable linear regression models (Supplementary Fig. 3).

Associations of the healthy dietary pattern scores with chemicals appeared to be more pronounced in Asian and Pacific Islanders (Supplementary Fig. 4a) than in other race or ethnic groups, with *P* for interaction <0.05 (Supplementary Fig. 4b). Group differences between different races and ethnicities were mainly observed in the associations of aHEI with plasma PCB and PFAS concentrations. Meanwhile, the race and ethnic heterogeneity in the associations between food groups and chemicals was more pronounced in fish and EPA + DHA (Supplementary Fig. 5).

Overall, primary findings on the associations of dietary pattern scores with chemical concentrations were consistent and did not change materially after applying inverse probability weighting to represent the total cohort population^8^ and after additional adjustment for total lipids (Supplementary Tables 5 and 6), modelling chemicals as a binary variable (≥80th percentile versus <80th percentile) (Supplementary Table 7), controlling for clinical centres as random effect intercept (Supplementary Table 8), or imputing chemical values below the limit of detection (Supplementary Table 9). After excluding nutrients that were used to calculate dietary pattern scores, fish consumption remained the highest loading factor, indicating the highest contribution to variation in total chemicals, total PCBs and total PFASs (Supplementary Fig. 6). Results of the elastic network regression analyses were in line with results from RRR (Supplementary Fig. 7).

## Discussion

In this large multi-racial pregnancy cohort in the United States, we observed that greater adherence to aMED, aHEI and DASH in peri-conception and early pregnancy was significantly associated with higher levels of plasma chemical concentrations. The associations were most pronounced for aMED and aHEI with PCBs and PFASs and appeared driven mainly by the associations with fish, EPA + DHA, the MUFA:SFA ratio and vegetables. These associations were more pronounced in the Asian and Pacific Islander population. Given that the World Health Organization and the Food and Agricultural Organization of the United Nations both recommend the consumption of a combination of healthy foods rather than single foods to avoid over-nutrition or nutritional deficiency^9^, findings from this study provide a more holistic portrayal of the interplay between diet and chemicals coming from diet.

Our study systematically examined the associations between dietary patterns and concentrations of diverse chemicals in a large sample of women. Our findings provide a significant clue for future investigations into the joint impacts of these factors on pregnancy and foetal outcomes. Only one previous study examined such associations, albeit using umbilical cord blood^10^. Studies among non-pregnant individuals are sparse, too, with only two studies identified and inconsistent associations reported^11,12^. Inferences from these studies were limited by their relatively small sample size, measured chemical classes, absence of food groups/nutrients and diet variations by regions and populations. The present study examined multiple purportedly healthy dietary patterns and diverse classes of chemicals comprehensively, which can facilitate the identification of toxic chemicals associated with healthy dietary patterns, shedding light on the previously overlooked aspect of single pattern or chemical class in similar investigations.

Seafood and aquatic products may have driven the associations of aMED and aHEI with PFASs and PCBs as the calculation of dietary pattern scores include fish and EPA + DHA (supplemental use was not included) for aMED and aHEI, respectively. The majority of EPA + DHA comes from fish and other seafood consumption. Fish consumption is one of the major sources of exposure to PFAS and PCB in humans among diverse populations of different ages^13,14^. Besides, inverse associations of aHEI, fish and EPA + DHA with the precursor NMeFOSAA suggest that the transformation reactions may have occurred already. While specific PFAS control standards for human blood have not been established, the US Environmental Protection Agency is consistently lowering environmental limits for PFAS exposures from food sources due to a growing body of evidence indicating their potential metabolic and reproductive toxicity, even at low dose^15^. Similarly, PCB concentrations in marine fish are generally higher than in other foods. Despite that most of the PCBs have been phased out for many years, people are still at risk of exposure due to historical emission and multi-media transportation^16^.

Even though fish is generally regarded as healthy food, it also carries specific heavy metals such as As and Hg owing to their bioaccumulation along the food chain^17^. Women with high aHEI scores in our study have comparable metal concentrations to participants of the National Health and Nutrition Examination Survey (NHANES) conducted in the same study period (2009–2012)^18^. Despite the low detection rates of toxic heavy metals in our present study, the extrapolation to the broader population suggests a potential public health threat and that raising the awareness of heavy metal contamination for healthy food in the general public is needed. Similarly, associations between OCPs and vegetable, whole fruit and the MUFA:SFA ratio in this study have shown that, even though some OCPs have been phased out, the US population is still at risk due to their historical release or transportation globally through various environmental media. Consistent associations were found in European and Asian populations^14,19^, suggesting a potentially worldwide threat to food safety.

The more pronounced associations among Asians and Pacific Islanders may be due to higher consumption of fish and EPA + DHA among the Asian and Pacific Islander ethnic groups than other races and ethnicities^16^. Thus, the health benefits of a given healthy dietary pattern may vary across ethnic groups, and population-specific targeted dietary guidance that considers the burden from potential pollutant exposure is needed to optimize health interventions.

This study is distinguished by several notable strengths. First, this study examined the association of three of the most widely recommended dietary patterns with a comprehensive profile of environmental chemicals in a relatively large multi-racial population. Second, we used a well-validated food frequency questionnaire (FFQ) to capture long-term habitual dietary patterns, and detailed information on covariates was assessed to control potential confounders. Third, we examined the associations of healthy dietary patterns with multiple chemicals simultaneously and further examined the associated drivers of food groups and nutrients.

Several potential limitations merit consideration. First, as with the nature of other observational studies, we cannot completely rule out the possibility of impacts from unmeasured confounders such as variables related to air and water sources of chemicals and chemical accumulation across organs. Second, given that our study was conducted on multi-racial cohort, it is possible that FFQ may not capture all the commonly consumed foods in each race and ethnicity group. It would be optimal to develop and apply race- and ethnicity-specific FFQ to assess habitual dietary patterns. Third, it is noteworthy that our population was exposed to relatively low chemical levels compared with other populations at the same period. Thus, caution should be exercised when extrapolating our findings. Though the effect size of associations was modest, considering the long-term and gradual accumulation of chemicals from diet over years, it underscores the significance of considering the combined impact of chemicals and dietary patterns on human health, particularly for countries where pollution levels are rising.

In conclusion, we observed that greater adherence to healthy dietary patterns was associated with higher chemical exposure, especially aMED and aHEI dietary patterns with PCBs and PFASs. The associations were probably driven by the consumption of fish and related ingredients and appeared to be more pronounced among Asian and Pacific Islanders. Strengthening the regulation and supervision of chemicals in fish (PCBs and PFASs) and vegetables (OCPs) is critical, especially for unregulated seafood markets, coastal dwellers and farmers. Collective efforts from both government and society along with unified worldwide regulatory efforts are pivotal in mitigating exposure to hazardous chemicals, particularly for vulnerable populations such as pregnant women. Findings from the present study suggest that future studies characterizing healthy diets should consider both healthy food and nutrient components and related chemical exposure to better optimize the health benefits of healthy diets.

## Methods

Detailed methods beyond the shortened description are provided in Supplementary Information. Briefly, the study was based on pregnant individuals from the Eunice Kennedy Shriver National Institute of Child Health and Human Development (NICHD) Fetal Growth Studies–Singletons, and 1,618 women with both chemical measurements and FFQ data were included (Supplementary Fig. 1)^8^. Averaged maternal age of the included women was 28 (standard deviation (s.d.) 9) years, and all recruited women provided written informed consent. This study was approved by the institutional review board of the National Institutes of Health, and all participating clinical sites with a Clinical Trial Registry registered (NCT 00912132). aMED, aHEI and DASH scores were derived from FFQ, and 88 of 97 chemicals of different classes with detection rates above 1% were analysed. Covariates were selected on the basis of a priori evidence outlined in a causal diagram using a directed acyclic graph (Supplementary Fig. 2).

Multivariable linear regression models were applied to assess associations of individual dietary pattern scores and food components with each of the chemicals. To aid in the interpretation, *β* coefficients were converted into per cent difference using the following formula: (e^*β*^ − 1) × 100. Stratified analyses for different covariates were conducted to explore potential effect modifications. All significant levels of the *P* values were adjusted by the Benjamini–Hochberg procedure. Meanwhile, we performed RRR analysis to assess the contribution of each constituent food group and nutrient to the variations of individual chemicals and chemical classes (Fig. 1b)^20^.

Several additional analyses were conducted to validate the robustness of our results, including analysis that (1) incorporated the population weight by inverse probability weighting; (2) additionally adjusted for total lipids; (3) dichotomized each of the chemicals according to the 80th percentile (high level: ≥80th, common level <80th); (4) additionally adjusted for clinical centres as a random effect intercept using generalized linear mixed models; (5) excluded EPA + DHA, trans fat and the MUFA:SFA ratio in RRR analyses and elastic network regression models; (6) applied multivariate imputation by chained equations to impute chemical values below the limit of detection.

### Reporting summary

Further information on research design is available in the Nature Portfolio Reporting Summary linked to this article.

### Supplementary information

Supplementary Information Supplementary study protocol, Tables 1–9, Figs. 1–7 and references.

Reporting Summary

## Data Availability

The data used in this study are not publicly available due to privacy and confidentiality agreements. Access to the data is restricted to protect the personal and health information of the participants, in accordance with ethical guidelines and regulations. Researchers interested in accessing the data may contact the corresponding author with a detailed request and may be required to sign a data use agreement to ensure the protection of participant confidentiality.

## Code availability

Open source codes and scripts used for the analyses or figures are publicly available at the GitHub repository (https://github.com/GuoqiYu2023/Nature-Food-2024).
