# Supplementary material for: Healthy dietary patterns are associated with exposure to environmental chemicals in a pregnancy cohort
Source: Nat Food. 2024 Jul 1;5(7):563–8. doi: 10.1038/s43016-024-01013-x (PMC11272572; doi:10.1038/s43016-024-01013-x)
Supplement: Supplementary file 1 — Supplementary study protocol, Tables 1–9, Figs. 1–7 and references. [file 43016_2024_1013_MOESM1_ESM.pdf]

nature food

Supplementary information https://doi.org/10.1038/s43016-024-01013-x

Healthy dietary patterns are associated with exposure to environmental chemicals in a pregnancy cohort

In the format provided by the authors and unedited

Supplementary study protocol

Study population

The study was based on pregnant individuals from the *Eunice Kennedy Shriver* National Institute of Child Health and Human Development (NICHD) Fetal Growth Studies–Singletons (FGS) (2009–2013). This prospective birth cohort recruited pregnant women from 12 US clinical centers and endeavored to establish racially specific fetal growth standards. More details about the cohort have been described elsewhere [1]. Briefly, women with chronic pre-gravid diseases, a history of pregnancy complications such as hypertension and diabetes, or any well-recognized lifestyle risk factors (active smoking, alcohol or illicit drug use) were excluded at the recruitment stage, leaving a total of 2,802 pregnant women recruited at early pregnancy (8-13 gestational weeks). All recruited women provided written informed consent. No compensation was provided to the participants. Institutional review board approval was obtained at the National Institutes of Health and all participating clinical sites with a Clinical Trial Registry registered (NCT 00912132).

Among the 2,802 participants, 2,707 had chemical measurements, including 2,696 with POPs and 2,203 with metal measurements. At recruitment, 1,699 of the total participants completed the semi-quantitative food frequency questionnaires (FFQ), which reflected the habitual diet during the past 3 months. Compared to the overall women recruited for dietary assessment, women who completed the FFQ at baseline were slightly older, had higher levels of education, and were more likely to be non-Hispanic white or Hispanic, but they were still largely representative of the total population [2]. The FFQ used was a modified version of the Diet History Questionnaire-II, which has been validated among nonpregnant individuals [3]. Participants reported how often they usually consumed each food as the number of times per day, week, or month, and the usual portion size. Nutrients were estimated from the FFQ using the Diet∗Calc software (National Cancer Institute). In total, 1,618 women with both chemical measurements and FFQ data were included for final analysis (Fig S1).

Healthy dietary patterns

In this study, three healthy dietary pattern scores were calculated: alternate Mediterranean diet (aMED), alternate Healthy Eating Index (aHEI) and Dietary Approaches to Stop Hypertension (DASH) (constituent food groups and nutrients were detailed in Table S1).

Both aHEI and aMED represent the guidelines that are more updated in time and have been shown to be consistently associated with lower risks of chronic diseases [4]. A higher value of each score indicates greater adherence to the healthy dietary pattern. aMED, adapted from the Mediterranean diet score originally proposed by Trichopoulou et al., is comprised of 8 components [vegetable, legume, fruit, nut, whole grain, red & processed meat, fish, the ratio of monounsaturated fatty acids to saturated fatty acids (MUFA: SFA ratio)] [5]. aHEI was subsequently developed based on the original Healthy Eating Index, with the use of more specific food items and the inclusion of alcohol and multivitamin use [6]. It is comprised of 10 food groups and nutrients [vegetable, whole fruit, whole grain, sugar-sweetened beverage (SSB, including juice), nut and legume, red & processed meat, trans-fat, eicosapentaenoic acid and docosahexaenoic acid (EPA+DHA), polyunsaturated fatty acids excluding eicosapentaenoic acid and docosahexaenoic acid (PUFA excluding EPA & DHA), and sodium]. Fatty acids, including EPA+DHA and PUFA excluding EPA & DHA, were mainly derived from food items of FFQ without supplement use included. The aMED score is calculated by totaling the scores for each dietary component, assigning a score of 1 when the consumption amount of a food group/nutrient exceeds its corresponding median, and a score of 0 when it falls below the median. The aHEI score is calculated by assigning points to specific dietary components and then summing the score of each component. DASH was derived from the sum of 8 food group scores [fruit, vegetable, nut and legume, whole grain, low fatty dairy, sodium, red & processed meat, and SSB (excluding juice)] and each of the calculated components were scaled from 1 (least healthy) to 5 (most healthy) according to the quintiles of food consumption amounts. Several common food groups, such as vegetables, whole grain and red & processed meat, were used in the scoring of different dietary patterns. Alcohol consumption was removed from aMED and aHEI as it remains controversial whether it should be part of a healthy diet for pregnant women [2].

Chemicals assessment

Chemicals were measured in maternal plasma samples collected at recruitment (8–13 weeks of gestation, median: 12.9) by the Wadsworth Center, New York State Department of Health, according to the standardized procedure. A total of 97 chemicals, including 11 organochlorine pesticide (OCPs) (beta-hexachlorocyclohexane [BetaHCH], gamma-hexachlorocyclohexane [GammaHCH], hexachlorobenzene [HCB], Oxychlordane, trans-chlordane, transnonachlor, p,p' dichlorodiphenyldichloroethylene [P_P_DDE], o,p'-dichlorodiphenyldichloroethane [O_P_DDD], p,p'-dichlorodiphenyldichloroethane

[P_P_DDD], p,p'- dichlorodiphenyltrichloroethane [P_P_DDT], and mirex); 10 polybrominated diphenyl ethers (PBDEs) (PBB153, BDE[28, 47, 85, 99, 100, 153, 154, 183, 209]); 44 PCBs (PCB [5_8, 18_17, 22, 31_28, 33_20, 37, 41_64, 44, 47_48_75, 49_43, 52_73, 66_80, 70_76, 74_61, B90_101_89, 93_95, 99, 85_120, 110, 118_106, 105_127, 114_122, 128, 137, 138_158, 146_161, 153, 156, 157, 167, 170, 172_192, 177, 180, 182_187, 183, 194, 195, 196_203, 199, 202, 206, 208, and 209]); 11 PFASs (N-methylperfluoro-1-octanesulfonamidoacetic acid [NMeFOSAA], perfluorodecanoic acid [PFDA], perfluorododecanoic acid [PFDoDA], perfluorodecane sulfonate [PFDS], perfluoroheptanoic acid [PFHpA], perfluorohexanesulfonic acid [PFHxS], perfluorononanoic acid [PFNA], perfluorooctanoic acid [PFOA], perfluorooctanesulfonic acid [PFOS], perfluorooctane sulfonamide [PFOSA], and perfluoroundecanoic acid [PFUnDA]); and 21 metals (Arsenic [As], Barium [Ba], Beryllium [Be], Cadmium [Cd], Cobalt [Co], Chromium [Cr], Cesium [Cs], Copper [Cu], Mercury [Hg], Manganese [Mn], Molybdenum [Mo], Lead [Pb], Platinum [Pt], Antimony [Sb], Selenium [Se], Tin [Sn], Tellurium [Te], Thallium [Tl], Uranium [U], Tungsten [W], Zinc [Zn]) were measured.

OCPs and PBDEs were analyzed using gas chromatography coupled with a mass spectrometer (GC-MS), while PCBs were analyzed by a JEOL UltraFocus high-resolution mass spectrometer [7,8]. PFAS were analyzed using ultra-performance liquid chromatography coupled with an electrospray triple quadrupole tandem mass spectrometry (UPLC-MS) [7,8]. Metals were analyzed using inductively coupled plasma-mass spectrometry (ICP-MS) [9].

Briefly, for the analysis of PCBs, PBDEs, and OCPs, 1 ml of plasma was spiked with a 13C-labeled internal standard mixture (250 pg each for OCPs, PBDEs, and PCBs), vortexed, and refrigerated overnight. Subsequently, 1 ml of 88% formic acid was added, and the mixture was sonicated for 15 minutes, followed by the addition of 2 ml of Milli-Q water. The samples were then subjected to solid-phase extraction (SPE) using cartridges packed with 1.3 g of Sepra C18-E (Rapid Trace SPE Workstation) and eluted with dichloromethane, which was then concentrated to 1 ml. The extracts were further purified using SPE cartridges packed with 0.2 g of silica gel/1.1 g of sulfuric acid silica gel and eluted with 30% dichloromethane in hexane, then concentrated to a final volume of 50 μl under a gentle stream of nitrogen.

PBDEs were analyzed using an Agilent Technologies (Atlanta, GA) gas chromatograph (GC 7890A) coupled with a mass spectrometer (MSD 5975). Analyte separation was performed on a Zebron 5MS (15 m, 0.25 mm i.d., and 0.10 μm film thickness; Phenomenex) capillary column. OCPs were analyzed with a Thermo Finnigan (Bremen, Germany) Trace GC Ultra coupled with a double focusing sector mass spectrometer (DFS), using a DB-5MS (30 m, 0.25 mm i.d., and 0.25 μm film thickness; Agilent Technologies) capillary column for analyte separation. PCBs were analyzed using a JEOL (Tokyo, Japan) UltraFocus high-resolution mass spectrometer (JMS-800D), with analyte separation achieved via an HP-5MS (30 m, 0.25 mm i.d., and 0.25 μm film thickness; Agilent Technologies) capillary column. Quantification of PBDEs, OCPs, and PCBs was based on the isotope dilution method with 13C-labeled internal standards. Two procedural blanks and SRM1958 were analyzed for every set of 27 samples.

For PFAS quantification, briefly, 200 μl of plasma was transferred into a polypropylene (PP) tube and spiked with 13C-labeled internal standards. To this mixture, 100 μl of 10% ammonia solution (v/v) was added. After 30 minutes, 780 μl of 1% ammonium formate in methanol (w/v) was added and vortexed. The sample was then centrifuged, and the supernatant was loaded onto a Hybrid-SPE cartridge (Supleco, Bellefonte, PA). The eluent was concentrated three times under a gentle stream of nitrogen. The target analytes in the eluate were quantified using an ultra-performance liquid chromatography system (Acquity I Class; Waters, Milford, MA, US) coupled with an electrospray triple quadrupole tandem mass spectrometer (API 5500; AB SCIEX, Framingham, MA, US). Analyte separation was achieved using an Acquity UPLC BEH C18 column (1.7 μm, 50×2.1 mm, Waters). Serum cotinine was measured using ultra-performance liquid chromatography coupled with electrospray triple quadrupole tandem mass spectrometry.

Concentrations of metals were measured in blood plasma samples collected during the late first trimester of pregnancy (median: 12 weeks' gestation) and stored at −70°C pending analysis. Plasma specimens were shipped to the Wadsworth Center, New York State Department of Health, for trace element analysis using inductively coupled plasma-mass spectrometry (ICP-MS), a multi-element method optimized for serum/plasma samples and validated for use in biomonitoring studies. The ICP-MS instrument was calibrated with

matrix-matched standards traceable to the National Institute of Standards and Technology (NIST). Levels of serum internal quality control materials were included in each analytical run, and 2% of all samples were analyzed in duplicate. Method validation was established using NIST standard reference materials, and method performance was assessed by successful participation in external proficiency testing programs for serum/plasma trace elements operated by the Center de Toxicologie du Québec, UK NEQAS for Trace Elements, German EQUALM, and the New York State proficiency testing program for trace elements. All metal(loid) measurements were above the limit of detection, calculated according to the International Standards Organization / International Union of Pure and Applied Chemistry harmonized guidelines.

Concentrations of POPs and metals in all samples were expressed in ng/mL. The level of quantifications (LOQs) was adopted in the formal analysis to determine the chemical detection rate and varied by analytes between 0.0025-0.05 ng/mL for OCPs, 0.0025-0.01 ng/mL for PBDEs, 0.005 ng/mL for PCBs, 0.007-0.01 ng/mL for PFASs and 0.01-340 ng/mL for metals. Chemicals with concentrations below LOQ were replaced with LOQ/sqrt (2). Total OCPs, total PBDEs, total PCBs, and total PFASs were calculated by summing over the detected chemical concentrations within each chemical class due to their similar food sources and environmental fate [8,10]. Metals, regardless of the route and sources of exposure and health effects, were totalled as well to reflect the general changes introduced by adherence to dietary patterns [11].

Some POPs such as OCPs, PBDEs and PCBs are lipophilic and the plasma concentrations of these chemicals might be influenced by lipids [12]. Thus, we further measured lipid concentrations using commercially available enzymatic methods [13] and calculated total lipids by the equation as follows: plasma total lipids (mg/dL) = plasma total cholesterol×2.27+plasma triglycerides+62.3 [14]. Plasma cotinine level was measured by LC-MS to confirm the status of active or passive smoking.

Covariates

Data on maternal characteristics and socio-economic status such as age, weight, height, education level, tobacco and alcohol use, physical activity level, and household income were

collected using standardized questionnaires at recruitment. Parity and disease history were extracted from medical records. Pre-pregnancy body mass index (BMI, kg/m^2) was calculated as weight in kilograms divided by the square of height in meters. Maternal race/ethnicity was self-identified by the participants, including Hispanic, non-Hispanic White, non-Hispanic Black and Asian & Pacific Islander. Maternal education level was grouped into high school or below, some college and undergraduate, graduate and postgraduate. Parity was classified into primiparous and multiparous. Maternal income was grouped into three levels based on the income during the past year. We classified an individual's status of tobacco exposure as yes or no based on whether the plasma cotinine concentration was above the limit of detection. Alcohol consumption was excluded due to a low proportion of drinkers (0.75%). Physical activity was measured using the validated Pregnancy Physical Activity Questionnaire and reported in weekly physical activity level (MET-hour/w). Total energy intake (kcal) was estimated from the same FFQ administered at recruitment.

Data analysis

Distributions of women's characteristics were reported according to quartiles of the different dietary pattern scores and tested using the Chi-square test for categorical characteristics or ANOVA for continuous ones. Chemicals (except for PFASs and metals) were corrected by total lipids at recruitment to account for variation in the concentrations due to lipid solubility. All POPs included were natural log-transformed and metals were natural log (1+metal) transformed to stabilize the variation and approximate normal distribution. All chemicals were scaled by standard deviation (SD) for final analyses. Analyses were performed on chemicals with a detection rate above 1%, including 11 OCPs (BetaHCH, GammaHCH, HCB, Oxychlordane, TransChlordane, TransNo_chlor, P_P_DDE, O_P_DDD, P_P_DDD, P_P_DDT, Mirex), 9 PBDEs (BDE28, BDE47, BDE100, BDE99, BDE85, PBB153, BDE154, BDE153, BDE183), 42 PCBs (PCB5_8, PCB18_17, PCB31_28, PCB33_20, PCB22, PCB52_73, PCB49_43, PCB47_48_75, PCB44, PCB41_64, PCB74_61, PCB70_76, PCB66_80, PCB93_95, PCB90_101_89, PCB99, PCB110, PCB118_106, PCB114_122, PCB105_127, PCB146_161, PCB153, PCB137, PCB138_158, PCB128, PCB167, PCB156, PCB157, PCB182_187, PCB183, PCB177, PCB172_192, PCB180, PCB170, PCB202, PCB199, PCB196_203, PCB195, PCB194, PCB208, PCB206, PCB209), 10 PFASs (NMeFOSAA, PFDS, PFDoDA, PFHpA, PFHxS, PFOS, PFOA, PFNA, PFDA, PFUnDA), and 16 metals (As, Ba, Cd, Co, Cr, Cs, Cu, Hg, Mn, Mo, Pb, Sb, Se, Sn, Tl, Zn). To estimate the percent difference in chemicals per SD change in dietary pattern scores, we

categorized the dietary pattern scores into binary low and high groups by the median of each dietary pattern score, and then estimated the group-specific chemical concentrations. Group differences were examined by non-parametric test.

Multivariable linear regression models were used to assess the associations of individual dietary pattern scores and food components with each of the chemicals. Models were adjusted for maternal age, physical activity level, pre-pregnancy BMI, education, income, parity, total energy intake, and tobacco exposure. Both dietary pattern scores and consumption of each food group were modelled as continuous variables in the main models. Covariates included in the models were selected based on a priori evidence depicted in a causal diagram using a directed acyclic graph (DAG) (Figure S2) [9]. To aid in the interpretation of the results, beta coefficients were converted into percent difference using the following formula: $(e^{\beta} - 1) \times 100$, which represents the percent difference in plasma chemical concentrations by 1-unit SD increase in each dietary pattern score or contributing food group/nutrient. Stratified analyses for different covariates (race/ethnicity, parity, pre-pregnancy BMI) were conducted to explore potential effect modifications. To account for the possible multiple comparisons, significant levels of the *P*-values were adjusted by the Benjamini-Hochberg procedure in all the association analyses.

To examine the association between the dietary pattern and plasma concentrations of chemicals at a finer scale, we performed reduced rank regression analysis (RRR) to assess the contribution of each constituent food group/nutrient to the variations of individual chemicals and chemical classes, which has been generally adopted to identify key food groups/nutrients and obtain factor scores which would describe the degree of a participant's adherence to each identified dietary pattern. The workflow of the RRR analysis is summarized in the conceptual diagram (Fig 1a) [15]. For the RRR analysis, natural log-transformed chemical plasma concentrations were included and the food group/nutrient consumption was standardized by default in the model set of SAS procedure pls. As RRR does not allow the incorporation of confounding variables directly, we used the residual method of adjustment to minimize the correlation between the dietary patterns and confounding variables [16]. To calculate the residuals, we regressed each of the chemicals on confounding covariates. The covariate-adjusted chemicals and all food groups/nutrients were included and modelled as dependent

and independent variables, respectively. We further derived the model loadings of each constitution food group/nutrient, which represent the strength and direction of contributions to variation in chemicals.

Several additional analyses were performed to assess the robustness of our results. 1) To account for the baseline difference between included and excluded participants, we calculated the population weight by inverse probability weighting and did additional analysis while incorporating weight to represent the total cohort population. 2) We additionally adjusted for total lipids as previous studies showed that incorporating both covariate-adjusted standardization and the inclusion of lipids as a covariate in the regression model might have low bias and perform well [17]. 3) The associations of dietary pattern scores with chemicals might be attenuated due to the relatively low concentrations of exposure in the study population. Thus, each of the chemicals was dichotomized according to the 80th percentile (high level: ≥ 80th, common level< 80th) to examine whether the associations would remain significant. 4) Since chemical concentration and dietary pattern may vary by geographical regions, we additionally adjusted for clinical centers as a random effect intercept using generalized linear mixed models. 5) Given that certain nutrients, such as EPA+DHA, PUFA excluding EPA & DHA, Trans-fat, and MUFA:SFA ratio, were derived from other food groups, their inclusion in the analysis may introduce collinearity concerns. To address this, we conducted RRR analyses by excluding these nutrients. Additionally, we employed elastic network regression (ENR) models to identify key food groups/nutrients and assess the reliability and robustness of our findings. Both inclusion and exclusion of nutrients were considered in the ENR models. 6) Multivariate Imputation by Chained Equations (MICE) was applied to impute chemical values below the limit of detection (LOD) by specifying the skewed distribution and setting an upper limit according to the LOQ of each chemical [18]. MICE is an imputation method often used in environmental health studies to impute pollutant values that fall below the LOD. Values that are below the LOD are typically assigned a value of LOD/2, which can introduce bias and impact the validity of the analysis. MICE can handle multivariate missing data, where the missingness is not completely at random, and can generate plausible values for the missing data while taking into account the correlation between variables and the uncertainty associated with imputed values [19]. The results were further pooled by Rubin's rule. All analyses were conducted using SAS version 9.4 (SAS Institute, Cary, NC, USA) and R 4.2.1.

Table S1. Categories and distribution of the consumption amounts of different food groups among the NICHD Fetal Growth Study–Singletons cohort. No supplements were included in the calculations of dietary pattern scores. aMED score was derived from 8 food groups: vegetable, legume, fruit, nut, whole grain, red & processed meat, fish, the ratio of monounsaturated fatty acids to saturated fatty acids (MUFA: SFA ratio); the aHEI score was derived from 10 food groups: vegetable, whole fruit, whole grain, sugar-sweetened beverage (SSB, including juice), nut and legume, red & processed meat, trans-fat, eicosapentaenoic acid and docosahexaenoic acid (EPA+DHA), polyunsaturated fatty acids excluding eicosapentaenoic acid and docosahexaenoic acid (PUFA excluding EPA & DHA), and sodium. All fatty acids, including n-3 fatty acids (EPA+DHA), and other PUFAs were estimated based on the food items included in the food frequency questionnaire. DASH score was derived from 8 food groups: fruit, vegetable, nut and legume, whole grain, low fatty dairy, sodium, red & processed meat, and SSB (excluding juice).

Dietary patterns	Variable	Evaluation	Mean (SD)	Median (IQR)	Min-Max
aMED	Vegetable, (serving/d)	Healthful	3.7 (4)	2.9 (2.9)	0.1-85.9
aMED	Fruit, (serving/d)	Healthful	6.7 (7.8)	4.5 (5.1)	0.2-97.4
aMED	Whole grain, (gram/d)	Healthful	26.9 (25.3)	20.6 (24.1)	0.6-353.2
aMED	Nut, (serving/d)	Healthful	0.4 (0.7)	0.2 (0.3)	0-14.8
aMED	Legume, (serving/d)	Healthful	0.3 (0.6)	0.1 (0.3)	0-9.5
aMED	Red & processed meat, (serving/d)	Unhealthful	2.4 (4.2)	1 (1.9)	0-35.1
aMED	Fish, (serving/d)	Healthful	0.3 (0.5)	0.2 (0.4)	0-13.7
aMED	MUFA: SFA ratio	Healthful	1.2 (0.3)	1.2 (0.3)	0.5-3.1
aHEI	Vegetable, (serving/d)	Healthful	3.7 (4)	2.9 (2.9)	0.1-85.9
aHEI	Whole fruit, (serving/d)	Healthful	1.2 (2.9)	0.2 (0.8)	0-35
aHEI	Whole grain, (gram/d)	Healthful	26.9 (25.3)	20.6 (24.1)	0.6-353.2
aHEI	SSB (including juice), (serving/d)	Unhealthful	4.1 (5.4)	2.6 (3.4)	0-62.9
aHEI	Nut and legume, (serving/d)	Healthful	0.7 (1)	0.4 (0.6)	0-21.5
aHEI	Red & processed meat, (serving/d)	Unhealthful	2.4 (4.2)	1 (1.9)	0-35.1
aHEI	PUFA excluding EPA & DHA, (%energy)	Healthful	6.9 (2)	6.8 (2.5)	1.3-21.4
aHEI	EPA+DHA, (mg/d)	Healthful	244.5 (329.8)	150 (270)	0-8170
aHEI	Trans-fat, (%energy)	Unhealthful	1.9 (0.6)	1.9 (0.8)	0.3-4.2
aHEI	Sodium, (mg/d)	Unhealthful	3659.7 (3673.5)	3055.8 (1950.9)	352.5-112798.7
DASH	Vegetable, (serving/d)	Healthful	3.7 (4)	2.9 (2.9)	0.1-85.9
DASH	Fruit, (serving/d)	Healthful	6.7 (7.8)	4.5 (5.1)	0.2-97.4
DASH	Whole grain, (gram/d)	Healthful	26.9 (25.3)	20.6 (24.1)	0.6-353.2
DASH	SSB (excluding juice), (serving/d)	Unhealthful	0.6 (1.1)	0.4 (0.5)	0-36.1
DASH	Nut and legume, (serving/d)	Healthful	0.7 (1)	0.4 (0.6)	0-21.5
DASH	Red & processed meat, (serving/d)	Unhealthful	2.4 (4.2)	1 (1.9)	0-35.1
DASH	Low fatty dairy, (serving/d)	Healthful	1 (1.5)	0.5 (1.2)	0-16.4
DASH	Sodium, (mg/d)	Unhealthful	3659.7 (3673.5)	3055.8 (1950.9)	352.5-112798.7

Table S2. Characteristics of study participants at enrollment (gestational weeks 8–13, n = 1,618) according to quartiles of the aMED, aHEI, and DASH scores among the NICHD Fetal Growth Study–Singletons cohort. The chi-square test and analysis of variance were used to examine the group differences. Two-sided *p*-values were reported. aMED: alternate Mediterranean diet; aHEI: alternate Healthy Eating Index; DASH: Dietary Approaches to Stop Hypertension. SD: standard deviation. Continuous variables were reported with mean (SD) and count (%) was reported for categorical variables.

**Maternal Characteristics**	Overall	aMED			aHEI			DASH		
		Q1 (n=356)	Q4 (n=650)	*P*-value	Q1 (n=404)	Q4 (n=404)	*P*-value	Q1 (n=363)	Q4 (n=476)	*p*-value
**Race/ethnicity, n (%)**				<0.001			<0.001			<0.001
Hispanic	496 (30.7)	108 (30.3)	179 (27.5)		100 (24.8)	103 (25.5)		83 (22.9)	148 (31.1)	
Non-Hispanic White	334 (20.6)	73 (20.5)	148 (22.8)		56 (13.9)	116 (28.7)		34 (9.4)	165 (34.7)	
Non-Hispanic Black	489 (30.2)	136 (38.2)	163 (25.1)		216 (53.5)	60 (14.9)		198 (54.6)	73 (15.3)	
Asian & Pacific Islander	299 (18.5)	39 (11.0)	160 (24.6)		32 (7.9)	125 (30.9)		48 (13.2)	90 (18.9)	
**Pre-pregnant BMI, n (%)**				<0.001			<0.001			0.002
Normal weight	918 (56.7)	173 (48.6)	411 (63.2)		202 (50)	269 (66.6)		186 (51.2)	299 (62.8)	
Overweight	434 (26.8)	103 (28.9)	164 (25.2)		120 (29.7)	86 (21.3)		109 (30)	122 (25.6)	
Obesity	266 (16.4)	80 (22.5)	75 (11.5)		82 (20.3)	49 (12.1)		68 (18.7)	55 (11.6)	
**Highest level of education, n (%)**				<0.001			<.0001			<0.001
High school or below	511 (31.6)	156 (43.8)	160 (24.6)		184 (45.5)	84 (20.8)		172 (47.4)	104 (21.9)	
Some college/undergraduate	856 (52.9)	169 (47.5)	350 (53.9)		195 (48.3)	204 (50.5)		171 (47.1)	248 (52.1)	
Graduate/post-graduate	251 (15.5)	31 (8.7)	140 (21.5)		25 (6.2)	116 (28.7)		20 (5.5)	124 (26.1)	
**Income during last year, n (%)**				<0.001			<.0001			<0.001
Less than 3000	467 (28.9)	124 (34.8)	149 (22.9)		170 (42.1)	72 (17.8)		160 (44.1)	89 (18.7)	
3000~74999	410 (25.3)	104 (29.2)	152 (23.4)		109 (27)	86 (21.3)		90 (24.8)	106 (22.3)	
75000~	494 (30.5)	73 (20.5)	246 (37.9)		61 (15.1)	180 (44.6)		51 (14.1)	215 (45.2)	
Unknown	247 (15.3)	55 (15.5)	103 (15.9)		64 (15.8)	66 (16.3)		62 (17.1)	66 (13.9)	
**Parity, n (%)**				0.15			0.30			0.49
Primiparous	734 (45.4)	151 (42.4)	317 (48.8)		192 (47.5)	190 (47)		163 (44.9)	229 (48.1)	
Multiparous	884 (54.6)	205 (57.6)	333 (51.2)		212 (52.5)	214 (53)		200 (55.1)	247 (51.9)	
**Tobacco exposure, n (%)**				<0.001			<0.001			<0.001
Not exposed	1119 (69.2)	209 (58.7)	488 (75.1)		225 (55.7)	338 (83.7)		185 (51)	386 (81.1)	
Exposed	475 (29.4)	142 (39.9)	152 (23.4)		175 (43.3)	60 (14.9)		175 (48.2)	82 (17.2)	
Unknown	24 (1.5)	5 (1.4)	10 (1.5)		4 (1)	6 (1.5)		3 (0.8)	8 (1.7)	
**Age at enrolment, years, mean (SD)**	28 (9)	25 (9)	30 (8)	<0.001	24 (8)	31 (7)	<0.001	24 (8)	30 (7)	<0.001
**Total physical activity, MET hour per week, mean (SD)**	290 (186.5)	290 (203.9)	298 (180.5)	<0.001	312 (212.7)	275 (157.9)	<0.001	295 (194)	292 (175.1)	0.15
**Total lipid concentration, mg/dl, mean (SD)**	1928 (1271.6)	1430 (953.7)	2359 (1468.6)	0.15	2130 (1715.1)	1847 (901.7)	0.08	1931 (1538.3)	2133 (1090.6)	0.998
**Total energy intake (TEI; kilocalories), mean (SD)**	601 (129.1)	596 (139.9)	598 (125.5)	0.19	587 (140)	607 (126.6)	0.03	583 (133.7)	610 (132.1)	0.009

Table S3. Plasma concentration differences of chemicals according to aMED, aHEI, and DASH scores among the NICHD Fetal Growth Study–Singletons cohort. Group differences of chemicals (High vs. Low, dichotomized by the median of each dietary pattern score) were examined by a non-parametric test. Estimations with raw *p*-value< 0.05 were bolded. To account for multiple comparisons, Benjamini-Hochberg (BH) adjusted *p*-values were calculated with *p*< 0.001, <0.01 and <0.05 marked as ***, **, and * respectively. All *p*-values were two-sided. Chemicals (ng/g lipid, except for PFASs and metals, ng/mL) were standardized by total lipids. aHEI: alternate Healthy Eating Index; aMED: alternate Mediterranean diet; DASH: Dietary Approaches to Stop Hypertension. Chemicals were reported with median± interquartile range (IQR). DR: detection rate. LOQ: limit of quantification (ng/mL).

					aMED			aHEI			DASH		
Chemical class	Chemical	Total population (DR%)	LOQ	Median (IQR)	Low aMED	High aMED	*P*	Low aHEI	High aHEI	*P*	Low DASH	High DASH	*P*
OCPs (11)	BetaHCH	1599 (55.91)	0.0025	0.96 (3.42)	0.74 (2.36)	1.21 (4.57)	**0.001****	0.59 (1.94)	1.51 (5.77)	**<0.001*****	0.89 (3.08)	1.28 (4.64)	0.144
	Gamma HCH	1599 (13.01)	0.0025	0.30 (0.08)	0.3 (0.09)	0.3 (0.08)	0.827	0.31 (0.09)	0.3 (0.07)	**0.042**	0.31 (0.08)	0.3 (0.07)	**0.046**
	HCB	1599 (78.36)	0.0025	6.33 (8.26)	5.56 (9.68)	6.59 (7.42)	**0.005***	6.1 (9.64)	6.48 (7.53)	**0.014***	6.2 (9.18)	6.56 (6.67)	0.128
	Oxychlordane	1599 (72.17)	0.005	2.22 (3.29)	2.21 (3.02)	2.22 (3.47)	0.609	2.16 (3.12)	2.3 (3.46)	0.306	2.15 (3.17)	2.42 (3.58)	0.148
	TransChlordane	1599 (31.08)	0.005	0.64 (0.72)	0.65 (0.7)	0.64 (0.78)	0.812	0.65 (0.83)	0.63 (0.66)	0.058	0.65 (0.85)	0.63 (0.37)	**0.019**
	TransNo_chlor	1599 (90.12)	0.005	4.39 (4.94)	3.98 (4.44)	4.56 (5.46)	**0.021***	4.18 (4.85)	4.47 (5.16)	0.464	4.26 (4.92)	4.54 (4.97)	0.77
	P_P_DDE	1599 (99.62)	0.005	75.42 (107.99)	66.59 (86.63)	83.22 (123.08)	**<0.001*****	63.59 (82.78)	88.78 (135.1)	**<0.001*****	71.71 (103.49)	85.4 (122.59)	**0.002***
	O_P_DDD	1599 (3.31)	0.0025	0.30 (0.07)	0.3 (0.07)	0.3 (0.07)	0.466	0.3 (0.07)	0.29 (0.07)	0.375	0.3 (0.07)	0.29 (0.07)	0.244
	P_P_DDD	1599 (28.39)	0.0025	0.32 (0.23)	0.31 (0.14)	0.32 (0.29)	**0.014***	0.32 (0.14)	0.32 (0.31)	0.075	0.32 (0.24)	0.32 (0.22)	0.639
	P_P_DDT	1599 (61.66)	0.0025	1.17 (2.30)	0.89 (1.8)	1.37 (2.64)	**<0.001*****	0.83 (1.59)	1.51 (3.05)	**<0.001*****	1.02 (2.18)	1.46 (2.61)	**0.011***
	Mirex	1599 (32.27)	0.0025	0.33 (0.45)	0.32 (0.32)	0.33 (0.57)	**0.03**	0.32 (0.23)	0.34 (0.7)	**<0.001*****	0.32 (0.38)	0.33 (0.62)	0.155
	Total OCPs			102.29 (128.60)	90.92 (109.14)	109.92 (139.99)	**<0.001*****	87.41 (100.86)	118.54 (155.67)	**<0.001*****	98.55 (121.66)	110.06 (142.71)	**0.004***
PBDEs (9)	BDE28	1607 (33.67)	0.0025	0.33 (0.65)	0.33 (0.68)	0.33 (0.63)	0.708	0.34 (0.72)	0.32 (0.48)	0.086	0.33 (0.66)	0.33 (0.59)	0.671
	BDE47	1607 (92.59)	0.0025	8.69 (13.72)	9.65 (14.3)	8.2 (13.28)	**0.005***	9.78 (14.46)	7.6 (12.34)	**<0.001*****	9.27 (14.56)	7.48 (11.12)	**<0.001*****
	BDE100	1607 (76.1)	0.0025	2.53 (4.33)	2.86 (4.21)	2.3 (4.15)	**0.001****	2.84 (3.98)	2.12 (4.19)	**<0.001*****	2.72 (4.29)	2.02 (3.8)	**<0.001*****
	BDE99	1607 (66.15)	0.005	2.19 (4.56)	2.47 (4.89)	2.1 (4.37)	0.07	2.55 (5.2)	1.98 (4.33)	**0.016***	2.42 (5.12)	1.83 (3.59)	**<0.001****
	BDE85	1607 (2.3)	0.005	0.59 (0.13)	0.59 (0.13)	0.59 (0.13)	0.548	0.6 (0.14)	0.58 (0.13)	0.211	0.59 (0.13)	0.58 (0.13)	0.158
	PBB153	1607 (5.35)	0.005	0.30 (0.07)	0.3 (0.08)	0.3 (0.07)	0.488	0.3 (0.07)	0.29 (0.07)	0.075	0.3 (0.07)	0.29 (0.07)	**0.03**
	BDE154	1607 (49.35)	0.01	1.58 (2.81)	1.67 (3.41)	1.54 (2.47)	0.128	1.69 (3.22)	1.51 (2.52)	0.112	1.65 (3.21)	1.51 (2.37)	0.055
	BDE153	1607 (52.46)	0.0025	1.88 (7.17)	2.59 (7.89)	0.67 (6.69)	0.107	2.37 (7.51)	0.41 (6.81)	0.105	2.1 (7.72)	0.39 (6.31)	**0.044**
	BDE183	1607 (14.13)	0.0025	0.30 (0.08)	0.31 (0.1)	0.3 (0.07)	**0.022***	0.31 (0.09)	0.3 (0.08)	**0.013***	0.31 (0.09)	0.3 (0.07)	**0.003***
	Total PBDEs			23.27 (33.69)	26.68 (34.86)	21.53 (31.2)	**<0.001*****	25.71 (34.19)	21.19 (31.09)	**<0.001*****	25.34 (35.18)	18.53 (29.22)	**<0.001*****
PCBs (42)	PCB5_8	1602 (26.15)	0.005	0.63 (0.33)	0.63 (0.35)	0.63 (0.3)	0.991	0.64 (0.44)	0.63 (0.27)	0.245	0.64 (0.41)	0.62 (0.22)	0.057
	PCB18_17	1602 (30.27)	0.005	0.65 (0.45)	0.64 (0.4)	0.65 (0.51)	0.75	0.65 (0.43)	0.64 (0.46)	0.479	0.65 (0.46)	0.64 (0.43)	0.313
	PCB31_28	1602 (45.19)	0.005	0.74 (1.33)	0.75 (1.3)	0.74 (1.35)	0.648	0.75 (1.24)	0.74 (1.39)	0.525	0.75 (1.34)	0.73 (1.32)	0.522
	PCB33_20	1602 (8.74)	0.005	0.60 (0.15)	0.6 (0.16)	0.6 (0.14)	0.796	0.6 (0.15)	0.59 (0.14)	0.432	0.6 (0.15)	0.59 (0.14)	0.242
	PCB22	1602 (1.94)	0.005	0.59 (0.13)	0.59 (0.13)	0.59 (0.13)	0.593	0.59 (0.13)	0.59 (0.13)	0.345	0.59 (0.13)	0.58 (0.13)	0.233
	PCB52_73	1602 (28.28)	0.005	0.64 (0.53)	0.64 (0.52)	0.64 (0.53)	0.929	0.64 (0.39)	0.65 (0.66)	0.494	0.65 (0.59)	0.64 (0.42)	0.159
	PCB49_43	1602 (9.24)	0.005	0.60 (0.15)	0.6 (0.16)	0.6 (0.14)	0.617	0.61 (0.15)	0.59 (0.15)	0.356	0.61 (0.16)	0.59 (0.14)	0.095
	PCB47_48_75	1602 (7.55)	0.005	0.60 (0.15)	0.59 (0.16)	0.6 (0.14)	0.829	0.6 (0.15)	0.59 (0.14)	0.424	0.6 (0.15)	0.59 (0.14)	0.165
	PCB44	1602 (12.67)	0.005	0.61 (0.17)	0.6 (0.17)	0.61 (0.16)	0.838	0.61 (0.17)	0.6 (0.16)	0.344	0.61 (0.17)	0.59 (0.14)	0.076
	PCB41_64	1602 (11.92)	0.005	0.61 (0.16)	0.6 (0.17)	0.61 (0.15)	0.906	0.61 (0.16)	0.6 (0.16)	0.38	0.61 (0.17)	0.59 (0.14)	0.064
	PCB74_61	1602 (60.11)	0.005	1.05 (1.25)	0.93 (1.08)	1.14 (1.33)	**<0.001*****	0.9 (0.94)	1.23 (1.48)	**<0.001*****	1.01 (1.14)	1.19 (1.35)	**0.003***
	PCB70_76	1602 (15.48)	0.005	0.61 (0.18)	0.61 (0.18)	0.61 (0.18)	0.678	0.61 (0.17)	0.61 (0.18)	0.695	0.61 (0.18)	0.6 (0.18)	0.403

Continued Table S3. Plasma concentration differences of chemicals according to aMED, aHEI, and DASH scores among the NICHD Fetal Growth Study–Singletons cohort. Group differences of chemicals (High vs. Low, dichotomized by the median of each dietary pattern score) were examined by a non-parametric test. Estimations with raw *p*-value< 0.05 were bolded. To account for multiple comparisons, Benjamini-Hochberg (BH) adjusted *p*-values were calculated with *p*< 0.001, <0.01 and <0.05 marked as ***, **, and * respectively. All *p*-values were two-sided. Chemicals (ng/g lipid, except for PFASs and metals, ng/mL) were standardized by total lipids. aHEI: alternate Healthy Eating Index; aMED: alternate Mediterranean diet; DASH: Dietary Approaches to Stop Hypertension. Chemicals were reported with median± interquartile range (IQR). DR: detection rate. LOQ: limit of quantification (ng/mL).

Chemical class	Chemical	Total population (DR%)	LOQ	Median (IQR)	aMED Low aMED	aMED High aMED	aMED *P*	aHEI Low aHEI	aHEI High aHEI	aHEI *P*	DASH Low DASH	DASH High DASH	DASH *P*
	PCB66_80	1602 (18.29)	0.005	0.62 (0.19)	0.61 (0.18)	0.63 (0.2)	0.079	0.61 (0.16)	0.63 (0.22)	**0.005***	0.62 (0.19)	0.62 (0.2)	0.752
	PCB93_95	1602 (21.60)	0.005	0.62 (0.23)	0.62 (0.22)	0.63 (0.23)	0.329	0.62 (0.22)	0.62 (0.23)	0.948	0.62 (0.23)	0.62 (0.22)	0.186
	PCB90_101_89	1602 (21.66)	0.005	0.62 (0.23)	0.61 (0.21)	0.63 (0.24)	0.067	0.62 (0.22)	0.63 (0.25)	0.347	0.62 (0.24)	0.62 (0.22)	0.371
	PCB99	1602 (59.18)	0.005	1.07 (1.24)	0.91 (1.08)	1.12 (1.4)	**<0.001*****	0.92 (1.05)	1.15 (1.5)	**<0.001*****	1.06 (1.19)	1.07 (1.33)	0.449
	PCB110	1602 (12.48)	0.005	0.61 (0.17)	0.6 (0.17)	0.61 (0.16)	0.51	0.61 (0.17)	0.6 (0.16)	0.664	0.61 (0.17)	0.59 (0.15)	0.115
	PCB118_106	1602 (76.72)	0.005	1.88 (2.31)	1.6 (2.04)	2.05 (2.38)	**<0.001*****	1.58 (1.99)	2.13 (2.55)	**<0.001*****	1.84 (2.26)	2 (2.46)	0.05
	PCB114_122	1601 (1.06)	0.005	0.59 (0.13)	0.59 (0.13)	0.59 (0.13)	0.776	0.59 (0.13)	0.59 (0.13)	0.337	0.59 (0.13)	0.58 (0.13)	0.274
	PCB105_127	1602 (35.39)	0.005	0.67 (0.47)	0.65 (0.31)	0.69 (0.57)	**<0.001*****	0.65 (0.31)	0.71 (0.65)	**<0.001*****	0.67 (0.41)	0.69 (0.56)	0.064
	PCB146_161	1602 (33.96)	0.005	0.67 (0.52)	0.63 (0.25)	0.69 (0.66)	**<0.001*****	0.64 (0.26)	0.72 (0.77)	**<0.001*****	0.66 (0.48)	0.68 (0.64)	0.169
	PCB153	1602 (93.95)	0.005	5.37 (6.66)	4.43 (5.03)	6.09 (7.59)	**<0.001*****	4.43 (4.97)	6.63 (8.26)	**<0.001*****	5.15 (6.24)	6.1 (7.79)	**<0.001*****
	PCB137	1602 (8.24)	0.005	0.6 (0.14)	0.6 (0.15)	0.61 (0.14)	0.119	0.6 (0.14)	0.6 (0.15)	0.288	0.6 (0.14)	0.6 (0.15)	0.862
	PCB138_158	1602 (93.82)	0.005	4.45 (5.32)	3.76 (4.15)	4.91 (6.06)	**<0.001*****	3.78 (4.07)	5.33 (6.19)	**<0.001*****	4.19 (4.85)	5.14 (6.22)	**<0.001*****
	PCB128	1602 (1.87)	0.005	0.59 (0.13)	0.59 (0.13)	0.59 (0.13)	0.464	0.59 (0.13)	0.59 (0.13)	0.688	0.59 (0.13)	0.58 (0.13)	0.381
	PCB167	1602 (4.62)	0.005	0.60 (0.14)	0.59 (0.14)	0.6 (0.13)	0.099	0.6 (0.13)	0.59 (0.14)	0.448	0.6 (0.14)	0.59 (0.14)	0.906
	PCB156	1602 (30.71)	0.005	0.65 (0.41)	0.63 (0.23)	0.67 (0.53)	**<0.001*****	0.64 (0.23)	0.68 (0.63)	**<0.001*****	0.65 (0.32)	0.69 (0.65)	**<0.001****
	PCB157	1602 (1.69)	0.005	0.59 (0.13)	0.59 (0.13)	0.59 (0.13)	0.58	0.59 (0.13)	0.59 (0.13)	0.499	0.59 (0.13)	0.58 (0.13)	0.185
	PCB182_187	1602 (63.17)	0.005	1.17 (1.62)	0.92 (1.21)	1.35 (1.82)	**<0.001*****	0.92 (1.15)	1.44 (1.95)	**<0.001*****	1.12 (1.54)	1.31 (1.78)	**0.001****
	PCB183	1602 (23.47)	0.005	0.63 (0.23)	0.61 (0.17)	0.65 (0.3)	**<0.001*****	0.62 (0.18)	0.65 (0.33)	**<0.001*****	0.63 (0.22)	0.64 (0.28)	0.372
	PCB177	1602 (8.93)	0.005	0.60 (0.15)	0.6 (0.14)	0.61 (0.15)	**0.022***	0.6 (0.14)	0.6 (0.16)	0.111	0.61 (0.15)	0.6 (0.15)	0.508
	PCB172_192	1601 (5.25)	0.005	0.60 (0.14)	0.59 (0.14)	0.6 (0.13)	0.089	0.6 (0.14)	0.59 (0.14)	0.608	0.6 (0.14)	0.59 (0.14)	0.963
	PCB180	1602 (94.94)	0.005	3.16 (3.81)	2.54 (3)	3.69 (4.3)	**<0.001*****	2.43 (2.75)	4.02 (4.51)	**<0.001*****	2.87 (3.44)	3.99 (4.32)	**<0.001*****
	PCB170	1602 (69.16)	0.005	1.28 (1.47)	1.07 (1.1)	1.47 (1.68)	**<0.001*****	0.99 (1.07)	1.59 (1.79)	**<0.001*****	1.17 (1.33)	1.56 (1.77)	**<0.001*****
	PCB202	1602 (5.49)	0.005	0.60 (0.14)	0.59 (0.14)	0.6 (0.14)	**0.042**	0.6 (0.14)	0.6 (0.14)	0.622	0.6 (0.14)	0.59 (0.14)	0.537
	PCB199	1602 (35.71)	0.005	0.68 (0.53)	0.64 (0.32)	0.71 (0.68)	**<0.001*****	0.65 (0.29)	0.73 (0.8)	**<0.001*****	0.67 (0.44)	0.71 (0.67)	**0.022**
	PCB196_203	1602 (41.01)	0.005	0.70 (0.60)	0.67 (0.41)	0.76 (0.75)	**<0.001*****	0.67 (0.42)	0.78 (0.83)	**<0.001*****	0.69 (0.54)	0.77 (0.79)	**0.006***
	PCB195	1601 (2.87)	0.005	0.59 (0.13)	0.59 (0.13)	0.59 (0.13)	0.211	0.6 (0.14)	0.59 (0.13)	0.766	0.59 (0.14)	0.59 (0.14)	0.537
	PCB194	1602 (35.08)	0.005	0.68 (0.46)	0.64 (0.26)	0.71 (0.58)	**<0.001*****	0.64 (0.25)	0.74 (0.64)	**<0.001*****	0.66 (0.36)	0.75 (0.63)	**<0.001*****
	PCB208	1602 (3.93)	0.005	0.59 (0.14)	0.59 (0.14)	0.6 (0.13)	0.113	0.6 (0.14)	0.59 (0.13)	0.918	0.6 (0.14)	0.59 (0.14)	0.501
	PCB206	1601 (14.87)	0.005	0.61 (0.17)	0.6 (0.16)	0.62 (0.2)	**<0.001*****	0.61 (0.16)	0.62 (0.2)	**0.004***	0.61 (0.16)	0.62 (0.21)	0.444
	PCB209	1601 (7.56)	0.005	0.60 (0.14)	0.59 (0.14)	0.61 (0.14)	**0.012***	0.6 (0.14)	0.6 (0.15)	0.367	0.6 (0.14)	0.59 (0.14)	0.355
	Total PCBs			27.00 (36.29)	21.21 (30.1)	29.96 (40.2)	**<0.001*****	20.86 (29.59)	32.9 (42.13)	**<0.001*****	25.33 (35.73)	29.95 (39)	**<0.001****
PFASs (10)	NMeFOSAA	1351 (77.72)	0.0226	0.05 (0.08)	0.06 (0.09)	0.05 (0.08)	**<0.001****	0.06 (0.1)	0.04 (0.08)	**<0.001*****	0.06 (0.09)	0.04 (0.08)	**<0.001*****
	PFDS	1594 (6.02)	0.0586	0.04 (0.00)	0.04 (0)	0.04 (0)	0.199	0.04 (0)	0.04 (0)	**0.003****	0.04 (0)	0.04 (0)	0.057
	PFDoDA	1594 (43.54)	0.025	0.02 (0.03)	0.02 (0.02)	0.03 (0.04)	**<0.001*****	0.02 (0.02)	0.03 (0.04)	**<0.001*****	0.02 (0.03)	0.02 (0.03)	0.605

Continued Table S3. Plasma concentration differences of chemicals according to aMED, aHEI, and DASH scores among the NICHD Fetal Growth Study–Singletons cohort. Group differences of chemicals (High vs. Low, dichotomized by the median of each dietary pattern score) were examined by a non-parametric test. Estimations with raw *p*-value< 0.05 were bolded. To account for multiple comparisons, Benjamini-Hochberg (BH) adjusted *p*-values were calculated with *p*< 0.001, <0.01 and <0.05 marked as ***, **, and * respectively. All *p*-values were two-sided. Chemicals (ng/g lipid, except for PFASs and metals, ng/mL) were standardized by total lipids. aHEI: alternate Healthy Eating Index; aMED: alternate Mediterranean diet; DASH: Dietary Approaches to Stop Hypertension. Chemicals were reported with median± interquartile range (IQR). DR: detection rate. LOQ: limit of quantification (ng/mL).

					aMED			Ahei			DASH		
Chemical class	Chemical	Total population (DR%)	LOQ	Median (IQR)	Low aMED	High aMED	*P*	Low aHEI	High aHEI	*P*	Low DASH	High DASH	*P*
	PFHpA	1594 (43.98)	0.0318	0.02 (0.04)	0.02 (0.05)	0.02 (0.04)	0.453	0.02 (0.05)	0.02 (0.04)	0.472	0.02 (0.04)	0.02 (0.04)	0.976
	PFHxS	1594 (100)	0.0499	0.74 (0.88)	0.82 (0.92)	0.71 (0.84)	**0.025**	0.73 (0.86)	0.76 (0.89)	0.958	0.75 (0.85)	0.73 (0.92)	0.902
	PFOS	1594 (100)	0.0224	4.75 (3.98)	4.48 (3.67)	4.84 (4.19)	**0.02***	4.69 (3.61)	4.81 (4.4)	**0.041**	4.8 (3.89)	4.66 (4.26)	0.475
	PFOA	1594 (99.94)	0.0236	1.78 (1.40)	1.73 (1.42)	1.81 (1.4)	**0.01***	1.72 (1.26)	1.89 (1.49)	**<0.001*****	1.73 (1.3)	1.92 (1.6)	**<0.001*****
	PFNA	1594 (100)	0.0263	0.75 (0.58)	0.71 (0.52)	0.78 (0.63)	**0.002****	0.7 (0.49)	0.82 (0.64)	**<0.001*****	0.73 (0.57)	0.77 (0.58)	0.18
	PFDA	1594 (97.37)	0.0269	0.22 (0.24)	0.2 (0.19)	0.24 (0.27)	**<0.001*****	0.2 (0.19)	0.25 (0.29)	**<0.001*****	0.22 (0.23)	0.24 (0.25)	0.076
	PFUnDA	1594 (96.55)	0.025	0.16 (0.24)	0.13 (0.16)	0.2 (0.28)	**<0.001*****	0.13 (0.17)	0.22 (0.32)	**<0.001*****	0.16 (0.24)	0.18 (0.22)	0.172
	Total PFASs			9.18 (6.68)	8.8 (6.66)	9.37 (6.92)	**0.021***	8.86 (5.63)	9.55 (7.69)	**0.002****	9.19 (6.33)	9.07 (7.37)	0.957
Metals (16)	As	1325 (16.98)	0.7	0.49 (0.00)	0.49 (0)	0.49 (0)	**<0.001*****	0.49 (0)	0.49 (0)	**<0.001*****	0.49 (0)	0.49 (0)	0.41
	Ba	1325 (84.83)	0.67	1.13 (0.84)	1.12 (0.84)	1.14 (0.84)	0.865	1.15 (0.85)	1.1 (0.84)	0.24	1.14 (0.87)	1.1 (0.81)	0.325
	Cd	1325 (1.81)	0.1	0.07 (0.00)	0.07 (0)	0.07 (0)	0.403	0.07 (0)	0.07 (0)	0.813	0.07 (0)	0.07 (0)	0.802
	Co	1325 (74.42)	0.23	0.26 (0.15)	0.26 (0.08)	0.26 (0.15)	0.624	0.27 (0.12)	0.26 (0.15)	0.226	0.26 (0.15)	0.26 (0.07)	0.689
	Cr	1311 (4.04)	1.3	0.92 (0.00)	0.92 (0)	0.92 (0)	0.09	0.92 (0)	0.92 (0)	0.934	0.92 (0)	0.92 (0)	0.706
	Cs	1325 (98.79)	0.17	0.37 (0.19)	0.34 (0.18)	0.39 (0.19)	**<0.001*****	0.33 (0.16)	0.42 (0.2)	**<0.001*****	0.35 (0.19)	0.42 (0.19)	**<0.001*****
	Cu	1325 (100)	216.7	1884.45 (465.81)	1894.77 (463.2)	1875.3 (463.61)	0.104	1903.31 (453.75)	1854.83 (459.92)	**0.008***	1894.42 (474.51)	1848.49 (453.42)	**0.02**
	Hg	1325 (42.64)	0.27	0.19 (0.23)	0.19 (0.17)	0.19 (0.26)	**<0.001*****	0.19 (0.14)	0.29 (0.33)	**<0.001*****	0.19 (0.22)	0.19 (0.26)	0.066
	Mn	1325 (11.17)	2	1.41 (0.00)	1.41 (0)	1.41 (0)	0.133	1.41 (0)	1.41 (0)	0.539	1.41 (0)	1.41 (0)	0.476
	Mo	1200 (100)	0.3	1.9 (1.25)	1.74 (1.18)	2.01 (1.3)	**<0.001*****	1.77 (1.16)	2.02 (1.28)	**<0.001*****	1.84 (1.21)	2.04 (1.28)	**0.001****
	Pb	1303 (1.53)	2	1.41 (0.00)	1.41 (0)	1.41 (0)	0.504	1.41 (0)	1.41 (0)	0.296	1.41 (0)	1.41 (0)	0.289
	Sb	1325 (100)	0.07	2.89 (1.06)	2.89 (1.07)	2.89 (1.05)	0.922	2.89 (1.08)	2.89 (1.04)	0.906	2.87 (1.05)	2.92 (1.14)	0.475
	Se	1325 (100)	37	122.19 (19.01)	122.74 (19.14)	121.64 (18.82)	0.321	122.84 (19.82)	121.54 (18.79)	0.058	121.85 (19.69)	122.58 (17.73)	0.347
	Sn	1288 (20.11)	0.27	0.19 (0.00)	0.19 (0)	0.19 (0)	0.51	0.19 (0)	0.19 (0)	0.114	0.19 (0)	0.19 (0)	0.558
	Tl	1325 (47.09)	0.027	0.02 (0.01)	0.02 (0.01)	0.02 (0.02)	**0.019***	0.02 (0.01)	0.03 (0.02)	**0.009***	0.02 (0.01)	0.02 (0.02)	0.965
	Zn	1325 (100)	340	789.22 (189.8)	816.79 (200.7)	776.05 (181.65)	**<0.001*****	812.66 (190.65)	774.81 (184.41)	**<0.001*****	810.49 (190.94)	753.17 (176)	**<0.001*****
	Total metals			2799.44 (510.16)	2826.88 (532.65)	2783.51 (523.24)	**0.005***	2823.3 (512.29)	2773.26 (544.91)	**0.001***	2821.15 (519.74)	2739.44 (539.44)	**<0.001*****

Table S4. Percent difference in grouped and individual plasma chemical concentrations per 1 SD increase in dietary pattern indices of aHEI, aMED, and DASH among the NICHD Fetal Growth Study–Singletons cohort. Multiple linear regression model was used. a, no covariate was adjusted; b, adjusted for maternal race/ethnicity, age, physical activity level, pre-pregnancy BMI, education level, income, parity, tobacco exposure, and total energy intake. Estimations with raw p-value< 0.05 were bolded. To account for multiple comparisons, Benjamini-Hochberg (BH) adjusted p-values were calculated with p< 0.001, <0.01 and <0.05 marked as ***, **, and * respectively. All p-values were two-sided. Chemicals (except for PFASs and metals) were standardized by total lipids, and all chemicals were further log-transformed and scaled. All chemicals were log-transformed. Change% [(exp(beta) − 1) × 100] was reported to benefit interpretation.

Chemicals	aMED Change% (95% CI)[a]	aMED Change% (95% CI)[b]	aHEI [Change% (95% CI)][a]	aHEI [Change% (95% CI)][b]	DASH [Change% (95% CI)][a]	DASH [Change% (95% CI)][b]
BetaHCH	**6.9 (4.0, 9.9)** ***	1.8 (-0.7, 4.4)	**2.3 (1.8, 2.9)** ***	**0.8 (0.3, 1.2)** **	**2.4 (1.3, 3.5)** ***	**1.2 (0.2, 2.2)**
GammaHCH	-1.8 (-4.5, 1.0)	0.0 (-2.9, 3.1)	**-0.5 (-1.0, -0.0)**	-0.1 (-0.7, 0.4)	**-1.2 (-2.3, -0.1)**	0.0 (-1.2, 1.2)
HCB	**6.4 (3.5, 9.4)** ***	1.0 (-1.5, 3.4)	**1.2 (0.6, 1.7)** ***	0.4 (-0.1, 0.8)	**2.0 (0.9, 3.1)** ***	0.9 (-0.1, 1.8)
Oxychlordane	2.2 (-0.6, 5.1)	-1.8 (-4.6, 1.0)	0.5 (-0.1, 1.0)	-0.4 (-0.9, 0.2)	**1.3 (0.2, 2.4)** *	-1.0 (-2.2, 0.1)
TransChlordane	-0.6 (-3.3, 2.2)	0.2 (-2.8, 3.3)	**-0.6 (-1.2, -0.1)** *	-0.2 (-0.8, 0.4)	**-1.4 (-2.4, -0.3)** *	-0.5 (-1.7, 0.8)
TransNo_chlor	**3.4 (0.5, 6.3)** *	-0.7 (-3.6, 2.2)	0.2 (-0.3, 0.8)	-0.4 (-0.9, 0.2)	0.4 (-0.7, 1.5)	-1.0 (-2.2, 0.2)
P_P_DDE	**7.0 (4.1, 10.0)** ***	2.1 (-0.5, 4.8)	**2.3 (1.8, 2.8)** ***	**0.9 (0.4, 1.4)** **	**3.0 (1.9, 4.1)** ***	**1.2 (0.1, 2.3)**
O_P_DDD	-1.5 (-4.2, 1.3)	-0.6 (-3.6, 2.5)	-0.4 (-1.0, 0.1)	-0.1 (-0.7, 0.4)	**-1.5 (-2.6, -0.4)** *	-0.8 (-2.1, 0.4)
P_P_DDD	1.7 (-1.1, 4.6)	2.4 (-0.6, 5.5)	**0.6 (0.1, 1.2)** *	**0.9 (0.3, 1.4)** **	-0.2 (-1.2, 0.9)	**1.3 (0.1, 2.5)**
P_P_DDT	**6.4 (3.5, 9.4)** ***	2.3 (-0.3, 5.1)	**2.1 (1.6, 2.6)** ***	**1.1 (0.5, 1.6)** ***	**2.4 (1.3, 3.5)** ***	**1.7 (0.6, 2.8)**
Mirex	**5.4 (2.5, 8.4)** ***	1.3 (-1.6, 4.4)	**1.5 (0.9, 2.0)** ***	**0.6 (0.0, 1.1)**	**1.4 (0.3, 2.5)** *	0.3 (-0.9, 1.5)
Total OCPs	**7.1 (4.2, 10.2)** ***	1.9 (-0.8, 4.6)	**2.3 (1.8, 2.8)** ***	**0.8 (0.3, 1.3)** **	**3.0 (1.9, 4.1)** ***	**1.2 (0.1, 2.3)**
BDE28	0.1 (-2.6, 3.0)	0.2 (-2.8, 3.2)	**-0.8 (-1.3, -0.3)** **	-0.2 (-0.8, 0.3)	-0.7 (-1.8, 0.4)	0.4 (-0.8, 1.6)
BDE47	**-3.7 (-6.3, -1.0)** *	-1.5 (-4.4, 1.4)	**-1.6 (-2.1, -1.1)** ***	-0.3 (-0.9, 0.2)	**-2.7 (-3.8, -1.7)** ***	-0.7 (-1.8, 0.5)
BDE100	**-4.9 (-7.5, -2.2)** **	-1.5 (-4.3, 1.4)	**-1.5 (-2.0, -1.0)** ***	-0.3 (-0.8, 0.2)	**-2.6 (-3.6, -1.5)** ***	-0.7 (-1.8, 0.5)
BDE99	**-2.9 (-5.6, -0.2)**	-1.3 (-4.2, 1.8)	**-0.9 (-1.4, -0.4)** ***	0.0 (-0.6, 0.6)	**-2.2 (-3.3, -1.2)** ***	-0.9 (-2.1, 0.4)
BDE85	0.8 (-1.9, 3.7)	1.1 (-1.9, 4.2)	-0.4 (-0.9, 0.1)	0.2 (-0.3, 0.8)	-1.0 (-2.1, 0.0)	0.1 (-1.1, 1.3)
PBB153	-2.0 (-4.7, 0.8)	-0.0 (-3.0, 3.0)	-0.1 (-0.6, 0.4)	0.3 (-0.3, 0.8)	-0.5 (-1.6, 0.5)	0.5 (-0.7, 1.8)
BDE154	**-4.0 (-6.6, -1.3)** **	-0.5 (-3.1, 2.1)	**-0.6 (-1.1, -0.1)** *	0.1 (-0.4, 0.6)	**-1.3 (-2.3, -0.2)** *	-0.2 (-1.2, 0.9)
BDE153	**-3.4 (-6.0, -0.6)** *	-1.6 (-4.5, 1.3)	**-0.7 (-1.3, -0.2)** **	-0.3 (-0.8, 0.3)	**-1.1 (-2.2, -0.0)**	-0.4 (-1.5, 0.8)
BDE183	**-5.9 (-8.5, -3.3)** ***	0.3 (-1.3, 1.9)	**-0.8 (-1.3, -0.3)** **	0.1 (-0.2, 0.5)	**-1.7 (-2.7, -0.6)** **	0.1 (-0.6, 0.7)
Total PBDEs	**-4.4 (-7.1, -1.7)** **	-1.8 (-4.6, 1.2)	**-1.6 (-2.1, -1.1)** ***	-0.3 (-0.8, 0.3)	**-3.0 (-4.0, -1.9)** ***	-0.9 (-2.0, 0.3)
PCB5_8	-0.9 (-3.6, 1.9)	**3.0 (0.1, 6.1)**	**-0.5 (-1.0, -0.0)**	0.5 (-0.1, 1.0)	**-1.8 (-2.9, -0.8)** **	0.6 (-0.6, 1.7)
PCB18_17	0.4 (-2.3, 3.3)	2.7 (-0.4, 5.9)	-0.3 (-0.8, 0.3)	0.4 (-0.2, 1.0)	-1.0 (-2.0, 0.1)	0.6 (-0.6, 1.9)
PCB31_28	1.0 (-1.8, 3.9)	2.7 (-0.4, 5.9)	0.1 (-0.4, 0.6)	0.4 (-0.1, 1.0)	-0.2 (-1.3, 0.9)	0.9 (-0.4, 2.1)
PCB33_20	0.6 (-2.1, 3.5)	**3.2 (0.1, 6.3)**	-0.2 (-0.7, 0.3)	**0.8 (0.2, 1.4)** *	-0.8 (-1.9, 0.3)	1.1 (-0.1, 2.4)
PCB22	1.3 (-1.5, 4.2)	**3.7 (0.7, 6.8)** *	-0.2 (-0.7, 0.3)	**1.0 (0.4, 1.6)** **	-0.9 (-2.0, 0.2)	**1.3 (0.1, 2.5)**
PCB52_73	-0.0 (-2.8, 2.8)	2.4 (-0.7, 5.6)	-0.1 (-0.6, 0.5)	0.5 (-0.1, 1.1)	-0.8 (-1.9, 0.3)	0.6 (-0.7, 1.8)
PCB49_43	-0.5 (-3.2, 2.3)	1.8 (-1.3, 4.9)	-0.3 (-0.8, 0.3)	**0.6 (0.1, 1.2)** *	**-1.1 (-2.2, -0.0)**	0.7 (-0.6, 1.9)
PCB47_48_75	-0.0 (-2.8, 2.8)	2.3 (-0.7, 5.5)	-0.1 (-0.6, 0.4)	**0.9 (0.3, 1.5)** **	-1.0 (-2.1, 0.1)	1.1 (-0.2, 2.3)
PCB44	-0.6 (-3.3, 2.2)	1.9 (-1.1, 5.1)	-0.2 (-0.7, 0.3)	**0.6 (0.1, 1.2)**	-1.1 (-2.1, 0.0)	0.7 (-0.6, 1.9)
PCB41_64	-0.6 (-3.3, 2.2)	2.0 (-1.0, 5.2)	-0.2 (-0.7, 0.3)	**0.7 (0.1, 1.3)** *	-0.9 (-2.0, 0.2)	0.9 (-0.4, 2.1)
PCB74_61	**7.9 (4.9, 10.9)** ***	**3.4 (0.5, 6.3)**	**2.0 (1.5, 2.6)** ***	**0.8 (0.3, 1.4)** **	**2.8 (1.7, 3.9)** ***	0.3 (-0.9, 1.4)
PCB70_76	1.1 (-1.7, 4.0)	2.9 (-0.2, 6.1)	0.2 (-0.4, 0.7)	**0.9 (0.3, 1.5)** **	-0.4 (-1.5, 0.7)	1.1 (-0.1, 2.4)
PCB66_80	**3.0 (0.1, 5.9)**	**3.9 (0.8, 7.1)** *	**1.0 (0.5, 1.5)** ***	**1.4 (0.9, 2.0)** ***	0.2 (-0.9, 1.3)	**1.3 (0.1, 2.6)**

Continued Table S4. Percent difference in grouped and individual plasma chemical concentrations per 1 SD increase in dietary pattern indices of aHEI, aMED, and DASH among the NICHD Fetal Growth Study–Singletons cohort. Multiple linear regression model was used. a, no covariate was adjusted; b, adjusted for maternal race/ethnicity, age, physical activity level, pre-pregnancy BMI, education level, income, parity, tobacco exposure, and total energy intake. Estimations with raw *p*-value< 0.05 were bolded. To account for multiple comparisons, Benjamini-Hochberg (BH) adjusted *p*-values were calculated with *p*< 0.001, <0.01 and <0.05 marked as ***, **, and * respectively. All *p*-values were two-sided. Chemicals (except for PFASs and metals) were standardized by total lipids, and all chemicals were further log-transformed and scaled. All chemicals were log-transformed. Change% [(exp(beta) − 1) × 100] was reported to benefit interpretation.

Chemicals	aMED Change% (95% CI)[a]	aMED Change% (95% CI)[b]	aHEI [Change% (95% CI)][a]	aHEI [Change% (95% CI)][b]	DASH [Change% (95% CI)][a]	DASH [Change% (95% CI)][b]
PCB93_95	1.3 (-1.4, 4.2)	2.9 (-0.2, 6.2)	-0.0 (-0.5, 0.5)	0.5 (-0.0, 1.1)	-0.6 (-1.7, 0.4)	0.5 (-0.7, 1.8)
PCB90_101_89	2.7 (-0.1, 5.6)	**4.2 (1.1, 7.5)** *	0.3 (-0.2, 0.9)	**0.9 (0.3, 1.5)** **	-0.4 (-1.5, 0.7)	0.9 (-0.4, 2.1)
PCB99	**6.6 (3.7, 9.6)** ***	**4.4 (1.3, 7.5)** *	**1.6 (1.1, 2.1)** ***	**1.1 (0.5, 1.7)** ***	0.8 (-0.3, 1.9)	-0.0 (-1.2, 1.2)
PCB110	0.8 (-2.0, 3.7)	**3.1 (0.0, 6.3)**	-0.2 (-0.7, 0.3)	0.6 (-0.0, 1.1)	-1.0 (-2.1, 0.1)	0.6 (-0.6, 1.8)
PCB118_106	**7.7 (4.8, 10.8)** ***	**4.3 (1.3, 7.4)** *	**2.0 (1.5, 2.5)** ***	**1.1 (0.6, 1.7)** ***	**1.9 (0.8, 3.0)** **	0.1 (-1.1, 1.3)
PCB114_122	-0.2 (-2.9, 2.6)	1.6 (-1.4, 4.6)	-0.2 (-0.8, 0.3)	**0.7 (0.1, 1.2)** *	**-1.1 (-2.2, -0.0)**	0.5 (-0.7, 1.7)
PCB105_127	**6.4 (3.5, 9.4)** ***	**4.3 (1.3, 7.5)** *	**1.9 (1.4, 2.4)** ***	**1.4 (0.8, 1.9)** ***	1.0 (-0.0, 2.1)	0.2 (-1.0, 1.4)
PCB146_161	**10.1 (7.1, 13.2)** ***	**5.5 (2.6, 8.5)** **	**2.6 (2.1, 3.1)** ***	**1.6 (1.0, 2.1)** ***	**1.8 (0.7, 2.9)** **	0.4 (-0.7, 1.6)
PCB153	**11.7 (8.6, 14.7***)**	**4.2 (1.4, 7.1)** *	**2.8 (2.3, 3.4)** ***	**1.2 (0.7, 1.7)** ***	**3.5 (2.4, 4.6)** ***	0.4 (-0.6, 1.5)
PCB137	0.8 (-1.9, 3.7)	1.8 (-1.2, 4.9)	0.4 (-0.1, 0.9)	**0.7 (0.2, 1.3)** *	-0.6 (-1.7, 0.5)	-0.3 (-1.5, 0.9)
PCB138_158	**11.2 (8.2, 14.3)** ***	**4.4 (1.6, 7.3)** *	**2.8 (2.3, 3.3)** ***	**1.3 (0.8, 1.8)** ***	**3.6 (2.5, 4.7)** ***	0.7 (-0.4, 1.9)
PCB128	1.5 (-1.3, 4.3)	3.0 (-0.1, 6.1)	0.2 (-0.3, 0.7)	**1.1 (0.5, 1.6)** ***	-1.0 (-2.1, 0.1)	0.6 (-0.6, 1.8)
PCB167	**3.7 (0.9, 6.6)** *	**4.3 (1.2, 7.5)** *	**0.8 (0.3, 1.3)** **	**1.2 (0.7, 1.8)** ***	-0.4 (-1.4, 0.7)	0.7 (-0.6, 1.9)
PCB156	**8.8 (5.8, 11.8)** ***	**3.8 (0.9, 6.8)** *	**2.2 (1.7, 2.7)** ***	**1.0 (0.5, 1.6)** ***	**3.0 (1.9, 4.1)** ***	0.4 (-0.8, 1.5)
PCB157	0.4 (-2.4, 3.2)	1.9 (-1.1, 5.0)	-0.1 (-0.6, 0.4)	**0.7 (0.2, 1.3)** *	**-1.2 (-2.3, -0.1)**	0.2 (-1.0, 1.4)
PCB182_187	**12.5 (9.5, 15.6)** ***	**6.2 (3.3, 9.1)** ***	**2.9 (2.4, 3.4)** ***	**1.5 (0.9, 2.0)** ***	**3.1 (2.0, 4.2)** ***	0.9 (-0.2, 2.0)
PCB183	**8.9 (5.9, 11.9)** ***	**4.5 (1.5, 7.5)** *	**2.0 (1.5, 2.5)** ***	**1.1 (0.6, 1.7)** ***	**1.6 (0.5, 2.7)** **	0.6 (-0.6, 1.8)
PCB177	**6.9 (4.0, 9.9)** ***	**5.4 (2.4, 8.6)** **	**1.4 (0.9, 1.9)** ***	**1.3 (0.7, 1.8)** ***	0.4 (-0.7, 1.5)	0.8 (-0.3, 2.1)
PCB172_192	**4.7 (1.8, 7.7)** **	**4.5 (1.4, 7.7)** *	**0.9 (0.3, 1.4)** **	**1.0 (0.4, 1.5)** **	0.4 (-0.7, 1.5)	1.1 (-0.1, 2.3)
PCB180	**14.7 (11.7, 17.9)** ***	**5.8 (3.2, 8.4)** ***	**3.4 (2.9, 3.9)** ***	**1.2 (0.7, 1.6)** ***	**5.1 (4.0, 6.2)** ***	**1.0 (0.0, 2.1)**
PCB170	**14.2 (11.2, 17.4)** ***	**5.8 (3.1, 8.6)** ***	**3.3 (2.8, 3.8)** ***	**1.2 (0.7, 1.7)** ***	**4.6 (3.5, 5.8)** ***	0.9 (-0.1, 2.0)
PCB202	**5.7 (2.8, 8.7)** ***	**5.2 (2.1, 8.3)** **	**0.9 (0.4, 1.4)** **	**1.0 (0.4, 1.5)** **	-0.1 (-1.2, 0.9)	0.7 (-0.5, 1.9)
PCB199	**11.4 (8.4, 14.5)** ***	**6.3 (3.4, 9.3)** ***	**2.4 (1.9, 3.0)** ***	**1.2 (0.7, 1.7)** ***	**2.5 (1.4, 3.6)** ***	0.9 (-0.2, 2.0)
PCB196_203	**11.4 (8.4, 14.5)** ***	**4.9 (2.0, 7.8)** **	**2.3 (1.8, 2.8)** ***	**0.9 (0.3, 1.4)** **	**2.9 (1.8, 4.1)** ***	0.6 (-0.5, 1.7)
PCB195	2.7 (-0.1, 5.6)	**3.5 (0.4, 6.6)**	0.2 (-0.3, 0.7)	**0.7 (0.2, 1.3)** *	-0.5 (-1.6, 0.6)	1.0 (-0.3, 2.2)
PCB194	**12.5 (9.4, 15.6)** ***	**6.3 (3.5, 9.3)** ***	**2.6 (2.1, 3.1)** ***	**1.1 (0.6, 1.6)** ***	**3.9 (2.8, 5.0)** ***	**1.6 (0.5, 2.7)**
PCB208	**3.5 (0.7, 6.4)** *	**4.4 (1.3, 7.6)** *	**0.6 (0.0, 1.1)**	**1.1 (0.5, 1.6)** ***	-0.6 (-1.7, 0.5)	0.8 (-0.4, 2.0)
PCB206	**7.3 (4.3, 10.3)** ***	**5.4 (2.4, 8.6)** **	**1.4 (0.9, 1.9)** ***	**1.1 (0.5, 1.7)** ***	1.0 (-0.1, 2.1)	**1.2 (0.0, 2.4)**
PCB209	**5.8 (2.9, 8.8)** ***	**5.2 (2.1, 8.4)** **	**1.0 (0.5, 1.6)** ***	**1.1 (0.5, 1.7)** ***	-0.1 (-1.2, 1.0)	0.7 (-0.5, 1.9)
Total PCBs	**12.9 (9.9, 16.0)** ***	**5.2 (2.5, 8.0)** **	**3.1 (2.6, 3.6)** ***	**1.3 (0.8, 1.8)** ***	**4.0 (2.9, 5.1)** ***	0.7 (-0.4, 1.8)
NMeFOSAA	**-5.4 (-8.2, -2.5)** ***	**-3.8 (-6.7, -0.7)**	**-1.9 (-2.5, -1.4)** ***	**-1.0 (-1.5, -0.4)** **	**-3.8 (-4.9, -2.7)** ***	**-2.4 (-3.6, -1.2)** *
PFDS	2.6 (-0.2, 5.5)	1.8 (-1.2, 4.9)	**0.9 (0.3, 1.4)** **	**0.8 (0.2, 1.4)** *	-0.7 (-1.7, 0.4)	-0.1 (-1.3, 1.1)
PFDoDA	**8.1 (5.1, 11.1)** ***	**4.7 (1.8, 7.7)** **	**2.0 (1.5, 2.5)** ***	**1.5 (0.9, 2.0)** ***	0.4 (-0.6, 1.5)	0.1 (-1.1, 1.2)
PFHpA	-1.5 (-4.2, 1.3)	**3.3 (0.5, 6.2)**	-0.4 (-0.9, 0.1)	0.2 (-0.3, 0.7)	0.0 (-1.1, 1.1)	**1.5 (0.4, 2.6)**
PFHxS	**-3.3 (-5.9, -0.6)** *	-1.6 (-4.2, 1.1)	-0.1 (-0.7, 0.4)	0.1 (-0.4, 0.6)	-0.5 (-1.6, 0.6)	-1.0 (-2.1, 0.0)

Continued Table S4. Percent difference in grouped and individual plasma chemical concentrations per 1 SD increase in dietary pattern indices of aHEI, aMED, and DASH among the NICHD Fetal Growth Study–Singletons cohort. Multiple linear regression model was used. a, no covariate was adjusted; b, adjusted for maternal race/ethnicity, age, physical activity level, pre-pregnancy BMI, education level, income, parity, tobacco exposure, and total energy intake. Estimations with raw *p*-value< 0.05 were bolded. To account for multiple comparisons, Benjamini-Hochberg (BH) adjusted *p*-values were calculated with *p*< 0.001, <0.01 and <0.05 marked as ***, **, and * respectively. All *p*-values were two-sided. Chemicals (except for PFASs and metals) were standardized by total lipids, and all chemicals were further log-transformed and scaled. All chemicals were log-transformed. Change% [(exp(beta) − 1) × 100] was reported to benefit interpretation.

Chemicals	aMED Change% (95% CI)[a]	aMED Change% (95% CI)[b]	aHEI [Change% (95% CI)][a]	aHEI [Change% (95% CI)][b]	DASH [Change% (95% CI)][a]	DASH [Change% (95% CI)][b]
PFOS	**3.6 (0.8, 6.5)** *	0.6 (-2.2, 3.5)	**0.8 (0.3, 1.3)** **	0.4 (-0.1, 0.9)	-0.4 (-1.5, 0.7)	**-2.0 (-3.1, -0.8)** *
PFOA	**4.8 (2.0, 7.8)** **	1.7 (-1.1, 4.6)	**1.2 (0.7, 1.7)** ***	0.4 (-0.1, 0.9)	**2.8 (1.7, 3.9)** ***	-0.0 (-1.1, 1.1)
PFNA	**4.8 (1.9, 7.7)** **	2.9 (-0.0, 6.0)	**1.6 (1.1, 2.1)** ***	**0.9 (0.3, 1.4)** **	**1.4 (0.3, 2.5)** *	-0.3 (-1.5, 0.8)
PFDA	**10.1 (7.1, 13.2)** ***	**4.5 (1.6, 7.5)** *	**2.3 (1.8, 2.8)** ***	**1.3 (0.8, 1.8)** ***	**1.9 (0.8, 3.0)** **	-0.4 (-1.5, 0.7)
PFUnDA	**11.5 (8.5, 14.6)** ***	**6.5 (3.6, 9.5)** ***	**2.9 (2.4, 3.4)** ***	**2.1 (1.6, 2.6)** ***	**1.5 (0.4, 2.6)** *	0.1 (-1.0, 1.2)
Total PFASs	**3.9 (0.8, 7.1)** *	0.5 (-2.5, 3.6)	**1.1 (0.5, 1.6)** ***	0.5 (-0.1, 1.1)	0.3 (-0.8, 1.5)	**-1.7 (-2.9, -0.5)**
As	**6.1 (2.9, 9.4)** ***	2.0 (-1.2, 5.3)	**1.9 (1.3, 2.4)** ***	**0.9 (0.3, 1.5)** **	0.8 (-0.4, 2.0)	-0.3 (-1.6, 1.0)
Ba	-0.0 (-3.1, 3.1)	-0.2 (-3.5, 3.3)	-0.3 (-0.9, 0.2)	-0.5 (-1.1, 0.2)	-0.6 (-1.8, 0.5)	-0.7 (-2.0, 0.6)
Cd	0.2 (-2.8, 3.3)	0.8 (-2.5, 4.3)	0.1 (-0.4, 0.7)	0.3 (-0.4, 0.9)	0.7 (-0.5, 1.9)	1.4 (-0.0, 2.7)
Co	-0.6 (-3.6, 2.5)	0.8 (-2.5, 4.3)	-0.4 (-1.0, 0.1)	-0.2 (-0.8, 0.4)	-0.5 (-1.7, 0.7)	0.5 (-0.9, 1.8)
Cr	1.9 (-1.2, 5.1)	**3.9 (0.4, 7.5)**	0.2 (-0.4, 0.8)	0.5 (-0.2, 1.1)	0.1 (-1.1, 1.2)	0.6 (-0.8, 1.9)
Cs	**9.1 (5.8, 12.4)** ***	2.9 (-0.2, 6.1)	**3.0 (2.5, 3.6)** ***	**1.2 (0.6, 1.7)** ***	**4.9 (3.7, 6.1)** ***	**1.3 (0.1, 2.5)**
Cu	**-4.0 (-6.9, -1.0)** *	-2.2 (-5.3, 1.0)	**-1.1 (-1.6, -0.5)** ***	-0.5 (-1.1, 0.1)	**-1.2 (-2.4, -0.1)**	0.0 (-1.2, 1.3)
Hg	**6.5 (3.3, 9.8)** ***	2.2 (-0.9, 5.5)	**2.4 (1.8, 2.9)** ***	**1.4 (0.8, 1.9)** ***	**1.4 (0.3, 2.6)** *	0.3 (-0.9, 1.6)
Mn	-2.6 (-5.6, 0.4)	-2.5 (-5.7, 0.9)	-0.1 (-0.6, 0.5)	-0.0 (-0.7, 0.6)	-0.4 (-1.6, 0.7)	-0.8 (-2.2, 0.5)
Mo	**6.0 (2.6, 9.4)** ***	2.7 (-0.7, 6.3)	**1.3 (0.7, 1.9)** ***	0.3 (-0.4, 0.9)	**2.5 (1.2, 3.7)** ***	1.1 (-0.3, 2.5)
Pb	-0.9 (-3.9, 2.2)	-0.5 (-3.8, 3.0)	-0.4 (-1.0, 0.2)	-0.2 (-0.8, 0.5)	-0.8 (-2.0, 0.4)	-0.5 (-1.8, 0.9)
Sb	-0.1 (-3.1, 3.1)	-0.9 (-4.2, 2.5)	0.1 (-0.5, 0.6)	0.0 (-0.6, 0.7)	0.8 (-0.3, 2.0)	0.5 (-0.9, 1.9)
Se	-0.9 (-3.9, 2.2)	-1.8 (-5.0, 1.5)	**-0.7 (-1.3, -0.2)** *	**-1.1 (-1.7, -0.5)** **	0.3 (-0.9, 1.5)	-0.9 (-2.2, 0.4)
Sn	-1.0 (-4.0, 2.2)	0.1 (-3.3, 3.6)	**-0.7 (-1.2, -0.1)** *	-0.4 (-1.0, 0.2)	-1.1 (-2.2, 0.1)	-0.7 (-2.1, 0.7)
Tl	2.4 (-0.7, 5.6)	1.1 (-2.2, 4.5)	**0.8 (0.2, 1.3)** *	0.5 (-0.1, 1.1)	0.6 (-0.5, 1.8)	1.0 (-0.3, 2.4)
Zn	**-6.1 (-8.9, -3.1)** ***	**-4.6 (-7.8, -1.4)** *	**-1.2 (-1.8, -0.7)** ***	**-0.6 (-1.3, -0.0)**	**-2.7 (-3.9, -1.6)** ***	**-1.6 (-2.9, -0.3)**
Total metals	**-6.6 (-9.5, -3.6)** ***	**-4.2 (-7.4, -1.0)** *	**-1.5 (-2.1, -0.9)** ***	**-0.7 (-1.3, -0.1)** *	**-2.6 (-3.8, -1.4)** ***	-0.8 (-2.2, 0.5)

Table S5. Associations of dietary pattern scores with chemicals with accounting for non-response of FFQ by inverse probability weighting among the NICHD Fetal Growth Study–Singletons cohort. Multiple linear regression model was applied with weight calculated and included in the model to represent the total population. Statistically significant associations were bolded with the significance of two-sided adjusted *p*-values indicated by the asterisk: *: *p*-value<0.05; **: *p*-value<0.01; ***: *p*-value<0.001, respectively. All models were adjusted for maternal age, physical activity level, pre-pregnancy BMI, education level, income, parity, total energy intake, and tobacco exposure; All chemicals were log-transformed. Chemicals (except for PFASs and metals) were standardized by total lipids, and all chemicals were further log-transformed and scaled. Change% [(exp(beta) − 1) × 100] was reported to benefit interpretation.

Chemicals	aMED [Change% (95% CI)]	aHEI [Change% (95% CI)]	DASH [Change% (95% CI)]
BetaHCH	0.7 (-1.7, 3.1)	**0.8 (0.3, 1.2)** **	**1.2 (0.2, 2.2)**
GammaHCH	0.2 (-2.8, 3.3)	-0.1 (-0.7, 0.4)	0.0 (-1.2, 1.2)
HCB	1.1 (-1.3, 3.5)	0.4 (-0.1, 0.8)	0.9 (-0.1, 1.8)
Oxychlordane	-2.0 (-5.5, 1.5)	-0.1 (-0.7, 0.4)	-0.8 (-2.1, 0.4)
TransChlordane	-0.5 (-3.4, 2.5)	-0.2 (-0.8, 0.4)	-0.5 (-1.7, 0.8)
TransNo_chlor	-1.5 (-4.3, 1.4)	-0.4 (-0.9, 0.2)	-1.0 (-2.2, 0.2)
P_P_DDE	0.9 (-2.2, 4.1)	0.3 (-0.3, 0.8)	0.5 (-0.7, 1.8)
O_P_DDD	-2.2 (-4.9, 0.5)	-0.4 (-0.9, 0.2)	-1.0 (-2.2, 0.1)
P_P_DDD	-0.7 (-4.0, 2.7)	-0.2 (-0.8, 0.5)	-0.5 (-1.8, 0.9)
P_P_DDT	**3.7 (0.6, 6.8)**	**1.4 (0.8, 1.9)** ***	0.2 (-1.0, 1.4)
Mirex	0.0 (-2.9, 3.0)	**0.6 (0.0, 1.1)**	0.3 (-0.9, 1.5)
Total OCPs	1.0 (-1.5, 3.6)	**0.8 (0.3, 1.3)** **	**1.2 (0.1, 2.3)**
BDE28	-0.3 (-3.2, 2.6)	-0.2 (-0.8, 0.3)	0.4 (-0.8, 1.6)
BDE47	-1.9 (-4.6, 1.0)	-0.3 (-0.9, 0.2)	-0.7 (-1.8, 0.5)
BDE100	-1.9 (-4.7, 0.9)	-0.3 (-0.8, 0.2)	-0.7 (-1.8, 0.5)
BDE99	-1.5 (-4.4, 1.4)	0.0 (-0.6, 0.6)	-0.9 (-2.1, 0.4)
BDE85	1.2 (-1.7, 4.2)	0.2 (-0.3, 0.8)	0.1 (-1.1, 1.3)
PBB153	1.3 (-1.7, 4.3)	**0.7 (0.1, 1.2)** *	0.5 (-0.7, 1.7)
BDE154	-0.4 (-2.9, 2.2)	0.1 (-0.4, 0.6)	-0.2 (-1.2, 0.9)
BDE153	-1.5 (-4.2, 1.4)	-0.3 (-0.8, 0.3)	-0.4 (-1.5, 0.8)
BDE183	-0.5 (-2.2, 1.3)	0.1 (-0.2, 0.5)	0.1 (-0.6, 0.7)
Total PBDEs	-2.2 (-5.0, 0.7)	-0.3 (-0.8, 0.3)	-0.9 (-2.0, 0.3)
PCB5_8	2.6 (-0.4, 5.7)	**0.9 (0.3, 1.5)** **	1.1 (-0.1, 2.4)
PCB18_17	3.0 (-0.0, 6.0)	0.4 (-0.2, 1.0)	0.6 (-0.6, 1.9)
PCB31_28	1.3 (-1.6, 4.3)	**0.6 (0.1, 1.2)**	0.7 (-0.6, 1.9)
PCB33_20	2.0 (-0.9, 5.0)	**0.9 (0.3, 1.5)** **	1.1 (-0.2, 2.3)
PCB22	2.1 (-0.9, 5.1)	**0.7 (0.1, 1.3)** *	0.9 (-0.4, 2.1)
PCB52_73	2.5 (-0.4, 5.4)	**0.8 (0.3, 1.4)** **	0.3 (-0.9, 1.4)
PCB49_43	**4.4 (1.4, 7.5)** *	**1.4 (0.9, 2.0)** ***	**1.3 (0.1, 2.6)**
PCB47_48_75	**3.8 (0.9, 6.8)** *	0.5 (-0.1, 1.0)	0.6 (-0.6, 1.7)
PCB44	1.7 (-1.3, 4.8)	0.5 (-0.1, 1.1)	0.6 (-0.7, 1.8)
PCB41_64	1.6 (-1.3, 4.6)	**0.6 (0.1, 1.2)** *	0.7 (-0.6, 1.9)
PCB74_61	**4.1 (1.2, 7.2)** *	**1.1 (0.5, 1.7)** ***	-0.0 (-1.2, 1.2)
PCB70_76	2.6 (-0.4, 5.7)	0.5 (-0.0, 1.1)	0.5 (-0.7, 1.8)
PCB66_80	**4.1 (1.1, 7.2)** *	**0.9 (0.3, 1.5)** **	0.9 (-0.4, 2.1)
PCB93_95	**4.9 (2.0, 7.8)** **	**1.5 (0.9, 2.0)** ***	0.1 (-1.1, 1.2)

Continued Table S5. Associations of dietary pattern scores with chemicals with accounting for non-response of FFQ by inverse probability weighting among the NICHD Fetal Growth Study–Singletons cohort. Multiple linear regression model was applied with weight calculated and included in the model to represent the total population. Statistically significant associations were bolded with the significance of two-sided adjusted *p*-values indicated by the asterisk: *: *p*-value<0.05; **: *p*-value<0.01; ***: *p*-value<0.001, respectively. All models were adjusted for maternal age, physical activity level, pre-pregnancy BMI, education level, income, parity, total energy intake, and tobacco exposure; All chemicals were log-transformed. Chemicals (except for PFASs and metals) were standardized by total lipids, and all chemicals were further log-transformed and scaled. Change% [(exp(beta) − 1) × 100] was reported to benefit interpretation.

Chemicals	aMED [Change% (95% CI)]	aHEI [Change% (95% CI)]	DASH [Change% (95% CI)]
PCB90_101_89	**4.3 (1.4, 7.2)** *	**1.3 (0.8, 1.8)** ***	-0.4 (-1.5, 0.7)
PCB99	1.6 (-1.3, 4.6)	**0.8 (0.2, 1.4)** *	-0.1 (-1.3, 1.1)
PCB110	2.5 (-0.5, 5.6)	**1.1 (0.5, 1.6)** ***	0.6 (-0.6, 1.8)
PCB118_106	**4.9 (2.1, 7.8)** **	**1.3 (0.8, 1.8)** ***	0.7 (-0.4, 1.9)
PCB114_122	0.9 (-2.1, 3.9)	**0.7 (0.2, 1.3)** *	-0.3 (-1.5, 0.9)
PCB105_127	**4.0 (1.1, 7.1)** *	**1.1 (0.6, 1.7)** ***	0.1 (-1.1, 1.3)
PCB146_161	1.6 (-1.4, 4.6)	**0.7 (0.2, 1.3)** *	0.2 (-1.0, 1.4)
PCB153	**3.5 (0.5, 6.6)**	**1.2 (0.7, 1.8)** ***	0.7 (-0.6, 1.9)
PCB137	**3.4 (0.8, 6.1)** *	**1.2 (0.7, 1.7)** ***	0.4 (-0.6, 1.5)
PCB138_158	**3.3 (0.4, 6.4)**	**1.0 (0.5, 1.6)** ***	0.4 (-0.8, 1.5)
PCB128	**5.6 (2.8, 8.5)** **	**1.6 (1.0, 2.1)** ***	0.4 (-0.7, 1.6)
PCB167	**4.6 (1.7, 7.7)** *	**1.3 (0.7, 1.8)** ***	0.8 (-0.3, 2.1)
PCB156	**4.8 (2.2, 7.5)** **	**1.2 (0.7, 1.7)** ***	0.9 (-0.1, 2.0)
PCB157	**3.8 (0.8, 6.9)** *	**1.0 (0.4, 1.5)** **	1.1 (-0.1, 2.3)
PCB182_187	2.9 (-0.1, 6.0)	**0.7 (0.2, 1.3)** *	1.0 (-0.3, 2.2)
PCB183	**4.2 (1.4, 7.1)** *	**0.9 (0.3, 1.4)** **	0.6 (-0.5, 1.7)
PCB177	**4.1 (1.3, 7.1)** *	**1.1 (0.6, 1.7)** ***	0.6 (-0.6, 1.8)
PCB172_192	**5.7 (3.0, 8.5)** **	**1.5 (0.9, 2.0)** ***	0.9 (-0.2, 2.0)
PCB180	**5.2 (2.4, 8.0)** **	**1.1 (0.6, 1.6)** ***	**1.6 (0.5, 2.7)**
PCB170	**4.5 (2.0, 7.1)** **	**1.2 (0.7, 1.6)** ***	**1.0 (0.0, 2.1)**
PCB202	**4.6 (1.7, 7.6)** *	**1.1 (0.5, 1.7)** ***	0.7 (-0.5, 1.9)
PCB199	**3.7 (0.7, 6.7)** *	**1.1 (0.5, 1.6)** ***	0.8 (-0.4, 2.0)
PCB196_203	**4.4 (1.5, 7.4)** *	**1.1 (0.5, 1.7)** ***	**1.2 (0.0, 2.4)**
PCB195	**4.6 (1.7, 7.6)** *	**1.0 (0.4, 1.5)** **	0.7 (-0.5, 1.9)
PCB194	**5.5 (2.7, 8.4)** **	**1.2 (0.7, 1.7)** ***	0.9 (-0.2, 2.0)
PCB208	2.8 (-0.3, 5.9)	0.4 (-0.1, 1.0)	0.9 (-0.4, 2.1)
PCB206	**3.4 (0.4, 6.4)**	**1.0 (0.4, 1.6)** **	**1.3 (0.1, 2.5)**
PCB209	**3.0 (0.1, 6.1)**	**0.8 (0.2, 1.4)** *	1.1 (-0.1, 2.4)
Total PCBs	**4.4 (1.8, 7.2)** **	**1.3 (0.8, 1.8)** ***	0.7 (-0.4, 1.8)
NMeFOSAA	**-3.6 (-6.5, -0.5)**	**-1.0 (-1.5, -0.4)** **	**-2.4 (-3.6, -1.2)** *
PFDS	2.8 (-0.1, 5.8)	**0.9 (0.3, 1.4)** **	-0.3 (-1.5, 0.8)
PFDoDA	-0.9 (-3.5, 1.8)	0.1 (-0.4, 0.6)	-1.0 (-2.1, 0.0)
PFHpA	1.8 (-0.9, 4.7)	0.4 (-0.1, 0.9)	-0.0 (-1.1, 1.1)
PFHxS	0.7 (-2.1, 3.5)	0.4 (-0.1, 0.9)	**-2.0 (-3.1, -0.8)** *
PFOS	1.3 (-1.3, 3.9)	**0.9 (0.4, 1.4)** **	**1.2 (0.1, 2.3)**
PFOA	1.7 (-1.2, 4.7)	**0.9 (0.3, 1.4)** **	**1.3 (0.1, 2.5)**

Continued Table S5. Associations of dietary pattern scores with chemicals with accounting for non-response of FFQ by inverse probability weighting among the NICHD Fetal Growth Study–Singletons cohort. Multiple linear regression model was applied with weight calculated and included in the model to represent the total population. Statistically significant associations were bolded with the significance of two-sided adjusted *p*-values indicated by the asterisk: *: *p*-value<0.05; **: *p*-value<0.01; ***: *p*-value<0.001, respectively. All models were adjusted for maternal age, physical activity level, pre-pregnancy BMI, education level, income, parity, total energy intake, and tobacco exposure; All chemicals were log-transformed. Chemicals (except for PFASs and metals) were standardized by total lipids, and all chemicals were further log-transformed and scaled. Change% [(exp(beta) − 1) × 100] was reported to benefit interpretation.

Chemicals	aMED [Change% (95% CI)]	aHEI [Change% (95% CI)]	DASH [Change% (95% CI)]
PFNA	**6.5 (3.7, 9.5)** ***	**2.1 (1.6, 2.6)** ***	0.1 (-1.0, 1.2)
PFDA	**3.8 (1.0, 6.7)** *	0.2 (-0.3, 0.7)	**1.5 (0.4, 2.6)**
PFUnDA	1.3 (-1.2, 4.0)	**1.1 (0.5, 1.6)** ***	**1.7 (0.6, 2.8)**
Total PFASs	0.8 (-2.2, 3.9)	0.5 (-0.1, 1.1)	**-1.7 (-2.9, -0.5)**
As	2.1 (-1.0, 5.4)	**0.9 (0.3, 1.5)** ***	-0.3 (-1.6, 1.0)
Ba	-0.1 (-3.4, 3.3)	-0.5 (-1.1, 0.2)	-0.7 (-2.0, 0.6)
Cd	0.9 (-2.4, 4.3)	0.3 (-0.4, 0.9)	1.4 (-0.0, 2.7)
Co	0.5 (-2.8, 3.9)	-0.2 (-0.8, 0.4)	0.5 (-0.9, 1.8)
Cr	**3.5 (0.1, 7.0)**	0.5 (-0.2, 1.1)	0.6 (-0.8, 1.9)
Cs	**3.6 (0.5, 6.8)**	**1.2 (0.6, 1.7)** ***	**1.3 (0.1, 2.5)**
Cu	-2.8 (-5.9, 0.3)	-0.5 (-1.1, 0.1)	0.0 (-1.2, 1.3)
Hg	2.5 (-0.6, 5.7)	**1.4 (0.8, 1.9)** ***	0.3 (-0.9, 1.6)
Mn	-2.4 (-5.7, 1.0)	-0.0 (-0.7, 0.6)	-0.8 (-2.2, 0.5)
Mo	2.9 (-0.5, 6.5)	0.3 (-0.4, 0.9)	1.1 (-0.3, 2.5)
Pb	2.9 (-0.1, 5.9)	0.6 (-0.0, 1.1)	0.6 (-0.6, 1.8)
Sb	-1.0 (-4.3, 2.4)	0.0 (-0.6, 0.7)	0.5 (-0.9, 1.9)
Se	-2.0 (-5.2, 1.3)	**-1.1 (-1.7, -0.5)** **	-0.9 (-2.2, 0.4)
Sn	0.4 (-2.9, 3.9)	-0.4 (-1.0, 0.2)	-0.7 (-2.1, 0.7)
Tl	1.4 (-1.9, 4.8)	0.5 (-0.1, 1.1)	1.0 (-0.3, 2.4)
Zn	**-4.8 (-7.9, -1.6)** *	**-0.6 (-1.3, -0.0)**	**-1.6 (-2.9, -0.3)**
Total metals	**-4.7 (-7.9, -1.5)** *	**-0.7 (-1.3, -0.1)** *	-0.8 (-2.2, 0.5)

Table S6. Associations of dietary patterns with each lipophilic chemical with additional adjustment for total lipids among the NICHD Fetal Growth Study–Singletons cohort. Multiple linear regression model was used. Two-sided *p*< 0.05 was bolded, with Benjamini-Hochberg (BH) adjusted *p* values calculated and marked as "*", "**", "***", if adjusted *p* value< 0.05, < 0.01, < 0.001, respectively. All models were adjusted for total lipids (except for PFASs and metals), maternal race/ethnicity, age, physical activity level, pre-pregnancy BMI, education level, income, parity, tobacco exposure, and total energy intake; All chemicals were log-transformed and scaled. change% [(exp(beta) − 1) × 100] was reported to benefit interpretation.

Chemicals	aMED [Change% (95% CI)]	aHEI [Change% (95% CI)]	DASH [Change% (95% CI)]
BetaHCH	1.7 (-0.8, 4.2)	**0.7 (0.3, 1.2)** **	**1.2 (0.2, 2.2)**
GammaHCH	-0.5 (-3.3, 2.5)	-0.3 (-0.8, 0.3)	-0.1 (-1.3, 1.1)
HCB	0.9 (-1.5, 3.4)	0.4 (-0.1, 0.8)	0.9 (-0.1, 1.8)
Oxychlordane	-1.8 (-4.5, 1.0)	-0.4 (-0.9, 0.2)	-1.0 (-2.1, 0.1)
TransChlordane	-0.1 (-3.1, 3.0)	-0.3 (-0.9, 0.3)	-0.5 (-1.7, 0.7)
TransNo_chlor	-0.7 (-3.6, 2.3)	-0.4 (-0.9, 0.2)	-1.0 (-2.2, 0.2)
P_P_DDE	2.1 (-0.6, 4.8)	**0.9 (0.4, 1.4)** **	**1.2 (0.1, 2.3)**
O_P_DDD	-1.4 (-4.2, 1.4)	-0.4 (-0.9, 0.1)	-1.0 (-2.1, 0.1)
P_P_DDD	2.1 (-0.9, 5.2)	**0.8 (0.2, 1.3)** *	**1.2 (0.0, 2.4)**
P_P_DDT	2.3 (-0.4, 5.0)	**1.0 (0.5, 1.5)** ***	**1.7 (0.6, 2.8)**
Mirex	1.0 (-1.9, 4.0)	0.5 (-0.1, 1.0)	0.3 (-0.9, 1.4)
Total OCPs	1.8 (-0.8, 4.5)	**0.8 (0.3, 1.3)** **	**1.2 (0.1, 2.3)**
BDE28	0.1 (-2.9, 3.1)	-0.3 (-0.8, 0.3)	0.4 (-0.8, 1.6)
BDE47	-1.5 (-4.3, 1.5)	-0.3 (-0.9, 0.2)	-0.7 (-1.8, 0.5)
BDE100	-1.4 (-4.3, 1.4)	-0.3 (-0.8, 0.3)	-0.7 (-1.8, 0.5)
BDE99	-1.4 (-4.3, 1.7)	-0.0 (-0.6, 0.5)	-0.9 (-2.1, 0.3)
BDE85	0.1 (-2.5, 2.7)	-0.1 (-0.6, 0.4)	-0.1 (-1.2, 0.9)
PBB153	-0.6 (-3.5, 2.3)	0.1 (-0.5, 0.6)	0.4 (-0.8, 1.6)
BDE154	-1.0 (-3.4, 1.6)	-0.0 (-0.5, 0.4)	-0.3 (-1.3, 0.8)
BDE153	-1.7 (-4.5, 1.2)	-0.3 (-0.8, 0.3)	-0.4 (-1.6, 0.8)
BDE183	-0.0 (-1.6, 1.6)	0.1 (-0.2, 0.3)	-0.0 (-0.6, 0.6)
Total PBDEs	-1.8 (-4.6, 1.1)	-0.3 (-0.8, 0.3)	-0.9 (-2.0, 0.3)
PCB5_8	2.5 (-0.3, 5.3)	0.3 (-0.2, 0.8)	0.4 (-0.7, 1.6)
PCB18_17	2.2 (-0.8, 5.3)	0.2 (-0.3, 0.8)	0.5 (-0.7, 1.7)
PCB31_28	2.4 (-0.6, 5.6)	0.3 (-0.2, 0.9)	0.8 (-0.4, 2.0)
PCB33_20	2.1 (-0.5, 4.8)	0.5 (-0.0, 1.0)	0.9 (-0.1, 2.0)
PCB22	**2.2 (0.3, 4.0)**	**0.5 (0.2, 0.8)** *	**0.9 (0.2, 1.6)**
PCB52_73	2.0 (-1.0, 5.0)	0.4 (-0.2, 0.9)	0.5 (-0.7, 1.7)
PCB49_43	0.9 (-1.8, 3.6)	0.4 (-0.2, 0.9)	0.4 (-0.6, 1.5)
PCB47_48_75	1.3 (-1.3, 3.9)	**0.6 (0.1, 1.1)** *	0.8 (-0.2, 1.9)
PCB44	1.1 (-1.7, 4.0)	0.4 (-0.1, 0.9)	0.5 (-0.7, 1.6)
PCB41_64	1.2 (-1.6, 4.1)	0.4 (-0.1, 1.0)	0.7 (-0.4, 1.8)
PCB74_61	**3.2 (0.3, 6.2)**	**0.8 (0.2, 1.3)** *	0.2 (-0.9, 1.4)
PCB70_76	2.2 (-0.7, 5.2)	**0.7 (0.1, 1.2)** *	1.0 (-0.2, 2.1)
PCB66_80	**3.3 (0.3, 6.3)**	**1.2 (0.7, 1.8)** ***	**1.2 (0.0, 2.4)**
PCB93_95	2.4 (-0.6, 5.5)	0.4 (-0.2, 0.9)	0.4 (-0.8, 1.6)
PCB90_101_89	**3.7 (0.7, 6.8)**	**0.7 (0.2, 1.3)** *	0.8 (-0.4, 2.0)
PCB99	**4.2 (1.2, 7.3)** *	**1.0 (0.5, 1.6)** **	-0.1 (-1.2, 1.1)

Continued Table S6. Associations of dietary patterns with each lipophilic chemical with additional adjustment for total lipids among the NICHD Fetal Growth Study–Singletons cohort. Multiple linear regression model was used. Two-sided $p < 0.05$ was bolded, with Benjamini-Hochberg (BH) adjusted p values calculated and marked as "*", "**", "***", if adjusted p value< 0.05, < 0.01, < 0.001, respectively. All models were adjusted for total lipids (except for PFASs and metals), maternal race/ethnicity, age, physical activity level, pre-pregnancy BMI, education level, income, parity, tobacco exposure, and total energy intake; All chemicals were log-transformed and scaled. change% [(exp(beta) − 1) × 100] was reported to benefit interpretation.

Chemicals	aMED [Change% (95% CI)]	aHEI [Change% (95% CI)]	DASH [Change% (95% CI)]
PCB110	2.4 (-0.5, 5.3)	0.3 (-0.2, 0.8)	0.4 (-0.7, 1.6)
PCB118_106	**4.2 (1.2, 7.3)** *	**1.1 (0.5, 1.7)** ***	0.1 (-1.1, 1.2)
PCB114_122	0.0 (-1.7, 1.8)	0.2 (-0.2, 0.5)	0.1 (-0.6, 0.8)
PCB105_127	**4.0 (1.0, 7.1)** *	**1.3 (0.7, 1.8)** ***	0.1 (-1.1, 1.3)
PCB146_161	**5.2 (2.3, 8.2)** **	**1.5 (0.9, 2.0)** ***	0.3 (-0.8, 1.5)
PCB153	**4.2 (1.4, 7.0)** *	**1.2 (0.7, 1.7)** ***	0.4 (-0.7, 1.5)
PCB137	0.9 (-1.8, 3.6)	0.4 (-0.1, 0.9)	-0.5 (-1.6, 0.6)
PCB138_158	**4.3 (1.5, 7.2)** *	**1.3 (0.7, 1.8)** ***	0.7 (-0.4, 1.8)
PCB128	1.5 (-0.5, 3.5)	**0.6 (0.2, 1.0)** **	0.3 (-0.5, 1.0)
PCB167	**3.1 (0.6, 5.6)** *	**0.9 (0.4, 1.3)** ***	0.4 (-0.6, 1.4)
PCB156	**3.5 (0.6, 6.4)**	**0.9 (0.4, 1.5)** **	0.3 (-0.8, 1.4)
PCB157	0.4 (-1.4, 2.2)	0.2 (-0.1, 0.6)	-0.1 (-0.9, 0.6)
PCB182_187	**6.0 (3.2, 8.9)** ***	**1.4 (0.9, 1.9)** ***	0.9 (-0.2, 2.0)
PCB183	**4.0 (1.1, 6.9)** *	**1.0 (0.4, 1.5)** **	0.5 (-0.7, 1.6)
PCB177	**4.6 (1.9, 7.5)** **	**1.0 (0.5, 1.5)** ***	0.7 (-0.4, 1.8)
PCB172_192	**3.5 (0.9, 6.1)** *	**0.6 (0.1, 1.1)** *	0.8 (-0.2, 1.9)
PCB180	**5.7 (3.1, 8.4)** ***	**1.1 (0.7, 1.6)** ***	**1.0 (0.0, 2.1)**
PCB170	**5.7 (3.0, 8.4)** ***	**1.2 (0.7, 1.7)** ***	0.9 (-0.1, 2.0)
PCB202	**4.2 (1.5, 6.8)** *	**0.7 (0.2, 1.1)** *	0.5 (-0.5, 1.5)
PCB199	**6.0 (3.1, 8.9)** ***	**1.1 (0.6, 1.6)** ***	0.8 (-0.3, 1.9)
PCB196_203	**4.6 (1.8, 7.5)** *	**0.8 (0.3, 1.3)** **	0.5 (-0.6, 1.7)
PCB195	2.1 (-0.1, 4.3)	0.3 (-0.1, 0.7)	0.6 (-0.2, 1.5)
PCB194	**6.0 (3.2, 8.9)** ***	**1.0 (0.5, 1.5)** ***	**1.5 (0.4, 2.6)**
PCB208	**3.2 (0.8, 5.7)** *	**0.7 (0.2, 1.1)** **	0.5 (-0.4, 1.5)
PCB206	**4.8 (1.9, 7.8)** **	**0.9 (0.4, 1.5)** **	1.1 (-0.1, 2.2)
PCB209	**4.3 (1.5, 7.1)** *	**0.8 (0.3, 1.3)** **	0.5 (-0.6, 1.5)
Total PCBs	**5.1 (2.4, 8.0)** **	**1.3 (0.8, 1.8)** ***	0.7 (-0.4, 1.8)

Table S7. Associations of dietary patterns with each chemical: high exposure level VS common exposure level among the NICHD Fetal Growth Study–Singletons cohort. Binary logistics regression model was used. Estimations with raw *p*-value< 0.05 were bolded. To account for multiple comparisons, Benjamini-Hochberg (BH) adjusted *p*-values were calculated with *p*< 0.001, <0.01 and <0.05 marked as ***, **, and * respectively. All *p*-values were two-sided. All models were adjusted for maternal race/ethnicity, age, physical activity level, pre-pregnancy BMI, education level, income, parity, tobacco exposure, and total energy intake; Chemicals (except for PFASs and metals) were standardized by total lipids, and all chemicals were further log-transformed and scaled. Each of the chemical was dichotomized according to the 80th percentage (high level: ≥ 80th, common level< 80th).

Chemicals	aMED [OR (95%CI)]	aHEI [OR (95%CI)]	DASH [OR (95%CI)]
BetaHCH	1.09 (0.98, 1.20)	**1.02 (1.01, 1.04)** *	1.04 (1.00, 1.08)
HCB	1.01 (0.94, 1.10)	1.00 (0.99, 1.02)	1.03 (0.99, 1.06)
Oxychlordane	1.00 (0.92, 1.09)	0.99 (0.98, 1.01)	**0.96 (0.93, 0.99)**
TransChlordane	1.03 (0.95, 1.12)	1.00 (0.98, 1.01)	0.99 (0.96, 1.03)
TransNo_chlor	1.01 (0.93, 1.09)	0.99 (0.98, 1.01)	0.98 (0.95, 1.01)
P_P_DDE	**1.12 (1.03, 1.22)** *	**1.03 (1.02, 1.05)** ***	1.01 (0.97, 1.04)
P_P_DDD	**1.14 (1.05, 1.24)** *	**1.03 (1.02, 1.05)** ***	1.01 (0.98, 1.04)
P_P_DDT	**1.15 (1.06, 1.25)** *	**1.04 (1.02, 1.05)** ***	1.02 (0.99, 1.06)
Mirex	1.05 (0.97, 1.14)	**1.02 (1.00, 1.03)** *	1.01 (0.98, 1.04)
BDE28	1.01 (0.93, 1.09)	1.00 (0.98, 1.01)	1.01 (0.98, 1.04)
BDE47	0.99 (0.91, 1.08)	1.00 (0.99, 1.02)	1.00 (0.97, 1.03)
BDE100	0.98 (0.90, 1.06)	1.00 (0.99, 1.02)	0.99 (0.96, 1.03)
BDE99	0.93 (0.85, 1.00)	0.99 (0.98, 1.01)	**0.95 (0.92, 0.98)**
BDE154	0.95 (0.86, 1.05)	1.00 (0.98, 1.01)	0.97 (0.94, 1.01)
BDE153	0.96 (0.89, 1.04)	0.99 (0.98, 1.01)	0.98 (0.94, 1.01)
PCB5_8	**1.10 (1.01, 1.19)**	**1.02 (1.00, 1.03)** *	1.01 (0.98, 1.04)
PCB18_17	**1.10 (1.02, 1.20)**	1.01 (0.99, 1.02)	1.02 (0.98, 1.05)
PCB31_28	**1.10 (1.01, 1.19)**	**1.02 (1.01, 1.04)** **	1.00 (0.97, 1.04)
PCB52_73	**1.10 (1.02, 1.20)**	1.01 (0.99, 1.02)	1.01 (0.98, 1.04)
PCB74_61	**1.11 (1.02, 1.20)**	**1.02 (1.01, 1.04)** **	1.00 (0.97, 1.04)
PCB93_95	**1.12 (1.03, 1.22)** *	**1.03 (1.02, 1.05)** ***	1.00 (0.97, 1.03)
PCB90_101_89	1.08 (1.00, 1.17)	**1.03 (1.02, 1.05)** ***	0.98 (0.95, 1.02)
PCB99	1.08 (0.99, 1.18)	1.00 (0.99, 1.02)	**1.05 (1.01, 1.09)**
PCB118_106	**1.16 (1.06, 1.26)** *	**1.04 (1.02, 1.06)** ***	1.02 (0.98, 1.05)
PCB105_127	**1.14 (1.05, 1.25)** *	**1.04 (1.02, 1.05)** ***	1.01 (0.98, 1.04)
PCB146_161	**1.16 (1.06, 1.26)** *	**1.04 (1.02, 1.05)** ***	1.03 (1.00, 1.07)
PCB153	**1.12 (1.03, 1.22)** *	**1.03 (1.01, 1.04)** **	1.03 (0.99, 1.07)
PCB138_158	**1.11 (1.02, 1.21)**	**1.02 (1.01, 1.04)** **	1.01 (0.98, 1.05)
PCB156	**1.10 (1.01, 1.20)**	**1.02 (1.01, 1.04)** *	1.01 (0.98, 1.05)
PCB182_187	**1.14 (1.05, 1.24)** *	**1.02 (1.01, 1.04)** *	1.02 (0.98, 1.05)
PCB183	**1.13 (1.04, 1.23)** *	**1.03 (1.01, 1.05)** **	1.01 (0.97, 1.04)
PCB180	**1.17 (1.07, 1.28)** *	**1.03 (1.01, 1.05)** **	**1.05 (1.01, 1.08)**
PCB170	1.08 (0.99, 1.17)	**1.02 (1.00, 1.04)** *	1.00 (0.96, 1.03)
PCB199	1.07 (0.98, 1.16)	1.01 (0.99, 1.02)	1.00 (0.97, 1.04)
PCB196_203	1.04 (0.96, 1.13)	1.01 (0.99, 1.02)	1.00 (0.97, 1.04)
PCB194	1.08 (1.00, 1.17)	1.01 (1.00, 1.03)	1.03 (1.00, 1.06)

Continued Table S7. Associations of dietary patterns with each chemical: high exposure level VS common exposure level among the NICHD Fetal Growth Study–Singletons cohort. Binary logistics regression model was used. Estimations with raw *p*-value< 0.05 were bolded. To account for multiple comparisons, Benjamini-Hochberg (BH) adjusted *p*-values were calculated with *p*< 0.001, <0.01 and <0.05 marked as ***, **, and * respectively. All *p*-values were two-sided. All models were adjusted for maternal race/ethnicity, age, physical activity level, pre-pregnancy BMI, education level, income, parity, tobacco exposure, and total energy intake; Chemicals (except for PFASs and metals) were standardized by total lipids, and all chemicals were further log-transformed and scaled. Each of the chemical was dichotomized according to the 80th percentage (high level: ≥ 80th, common level< 80th).

Chemicals	aMED [OR (95%CI)]	aHEI [OR (95%CI)]	DASH [OR (95%CI)]
NMeFOSAA	0.98 (0.89, 1.07)	0.99 (0.98, 1.01)	0.98 (0.94, 1.01)
PFDoDA	1.05 (0.97, 1.14)	**1.02 (1.00, 1.03)** *	1.00 (0.96, 1.03)
PFHpA	1.02 (0.94, 1.11)	1.01 (0.99, 1.02)	1.00 (0.97, 1.04)
PFHxS	1.05 (0.97, 1.14)	1.01 (1.00, 1.03)	0.99 (0.96, 1.02)
PFOS	1.05 (0.96, 1.15)	**1.02 (1.00, 1.04)**	**1.04 (1.00, 1.08)**
PFOA	**1.12 (1.03, 1.22)** *	**1.03 (1.01, 1.05)** **	**1.04 (1.01, 1.07)**
PFNA	**1.18 (1.08, 1.29)** *	**1.05 (1.03, 1.07)** ***	1.01 (0.97, 1.04)
PFDA	0.96 (0.88, 1.05)	1.01 (0.99, 1.02)	0.99 (0.95, 1.02)
PFUnDA	**1.15 (1.05, 1.27)** *	**1.03 (1.01, 1.05)** **	**1.07 (1.03, 1.11)** *
Ba	1.04 (0.95, 1.13)	1.00 (0.98, 1.01)	1.01 (0.98, 1.04)
Co	1.07 (0.98, 1.17)	1.00 (0.99, 1.02)	1.02 (0.98, 1.05)
Cs	1.04 (0.94, 1.14)	**1.02 (1.01, 1.04)** *	1.01 (0.98, 1.05)
Cu	0.96 (0.88, 1.05)	0.99 (0.98, 1.01)	0.99 (0.95, 1.02)
Hg	1.05 (0.96, 1.15)	**1.04 (1.02, 1.06)** ***	1.01 (0.97, 1.04)
Mo	1.07 (0.97, 1.17)	1.00 (0.99, 1.02)	1.01 (0.98, 1.05)
Sb	1.02 (0.94, 1.11)	1.00 (0.98, 1.01)	**1.04 (1.00, 1.07)**
Se	1.02 (0.93, 1.11)	**0.98 (0.96, 0.99)** **	0.99 (0.95, 1.02)
Sn	1.02 (0.94, 1.12)	1.00 (0.98, 1.01)	0.99 (0.96, 1.03)
Tl	1.05 (0.97, 1.15)	1.02 (1.00, 1.03)	1.03 (0.99, 1.06)
Zn	0.92 (0.84, 1.00)	0.99 (0.97, 1.01)	**0.96 (0.93, 0.99)**

Table S8. Associations of dietary patterns with each chemical and chemical class while including clinical centers as random effect intercept among the NICHD Fetal Growth Study–Singletons cohort. Generalized Linear Mixed Models (GLMM) was used with clinical centers included as a random effect intercept. Estimations with raw *p*-value< 0.05 were bolded. To account for multiple comparisons, Benjamini-Hochberg (BH) adjusted *p*-values were calculated with *p*< 0.001, <0.01 and <0.05 marked as ***, **, and * respectively. All *p*-values were two-sided. All models were adjusted for total lipids (except for PFASs and metals), maternal race/ethnicity, age, physical activity level, pre-pregnancy BMI, education level, income, parity, tobacco exposure, and total energy intake; All chemicals were log-transformed and scaled. change% [(exp(beta) − 1) × 100] was reported to benefit interpretation.

Chemicals	aMED [Change% (95% CI)]	aHEI [Change% (95% CI)]	DASH [Change% (95% CI)]
BetaHCH	1.7 (-0.8, 4.2)	**0.7 (0.2, 1.1)** *	**1.3 (0.3, 2.3)**
GammaHCH	-0.4 (-3.3, 2.7)	-0.1 (-0.6, 0.5)	-0.0 (-1.2, 1.2)
HCB	0.8 (-1.7, 3.2)	0.3 (-0.1, 0.8)	0.8 (-0.2, 1.8)
Oxychlordane	-1.2 (-4.2, 1.8)	-0.2 (-0.8, 0.4)	-1.0 (-2.2, 0.2)
TransChlordane	0.3 (-2.7, 3.4)	-0.1 (-0.7, 0.4)	-0.5 (-1.7, 0.8)
TransNo_chlor	-0.4 (-3.3, 2.5)	-0.1 (-0.7, 0.4)	-0.7 (-1.8, 0.5)
P_P_DDE	-0.1 (-3.1, 3.0)	0.3 (-0.3, 0.8)	0.5 (-0.7, 1.8)
O_P_DDD	-1.5 (-4.2, 1.3)	-0.1 (-0.7, 0.4)	-0.6 (-1.7, 0.5)
P_P_DDD	-0.4 (-3.8, 3.1)	-0.2 (-0.8, 0.5)	-0.5 (-1.8, 0.9)
P_P_DDT	**4.4 (1.4, 7.6)** *	**1.3 (0.8, 1.9)** ***	0.2 (-1.0, 1.4)
Mirex	0.6 (-2.3, 3.6)	0.5 (-0.1, 1.0)	0.2 (-1.0, 1.4)
Total OCPs	1.7 (-0.9, 4.3)	**0.7 (0.2, 1.2)** **	**1.1 (0.1, 2.1)**
BDE28	-0.1 (-3.0, 2.8)	-0.2 (-0.7, 0.3)	0.5 (-0.7, 1.6)
BDE47	-1.2 (-4.0, 1.6)	-0.1 (-0.6, 0.4)	-0.4 (-1.6, 0.7)
BDE100	-1.6 (-4.4, 1.2)	-0.2 (-0.8, 0.3)	-0.5 (-1.7, 0.6)
BDE99	-1.2 (-4.2, 1.8)	0.1 (-0.5, 0.6)	-0.8 (-2.0, 0.4)
BDE85	1.1 (-1.9, 4.2)	0.3 (-0.3, 0.8)	0.1 (-1.1, 1.4)
PBB153	1.5 (-1.5, 4.6)	**0.7 (0.1, 1.3)** *	0.5 (-0.7, 1.7)
BDE154	-0.6 (-3.1, 2.0)	0.1 (-0.4, 0.6)	-0.1 (-1.2, 0.9)
BDE153	-2.1 (-4.9, 0.7)	-0.2 (-0.7, 0.3)	-0.4 (-1.5, 0.8)
BDE183	-0.0 (-1.6, 1.6)	0.2 (-0.1, 0.5)	-0.1 (-0.7, 0.6)
Total PBDEs	-1.6 (-4.3, 1.3)	-0.1 (-0.6, 0.5)	-0.6 (-1.8, 0.5)
PCB5_8	2.8 (-0.3, 6.0)	**0.9 (0.3, 1.5)** **	1.2 (-0.1, 2.4)
PCB18_17	2.7 (-0.4, 5.9)	0.4 (-0.2, 1.0)	0.6 (-0.6, 1.9)
PCB31_28	2.0 (-1.1, 5.2)	**0.7 (0.1, 1.3)** *	0.8 (-0.5, 2.0)
PCB33_20	2.4 (-0.7, 5.5)	**1.0 (0.4, 1.6)** **	1.1 (-0.1, 2.4)
PCB22	2.1 (-1.0, 5.2)	**0.8 (0.2, 1.4)** *	1.0 (-0.2, 2.3)
PCB52_73	**3.4 (0.5, 6.3)**	**0.8 (0.3, 1.4)** **	0.3 (-0.8, 1.5)
PCB49_43	**3.9 (0.8, 7.2)** *	**1.4 (0.9, 2.0)** ***	**1.4 (0.1, 2.6)**
PCB47_48_75	**3.1 (0.1, 6.1)**	**0.6 (0.0, 1.1)**	0.7 (-0.5, 1.9)
PCB44	2.5 (-0.6, 5.7)	0.6 (-0.0, 1.2)	0.7 (-0.6, 1.9)
PCB41_64	1.8 (-1.3, 4.9)	**0.7 (0.2, 1.3)** *	0.8 (-0.5, 2.0)
PCB74_61	**4.3 (1.3, 7.4)** *	**1.1 (0.5, 1.7)** ***	-0.1 (-1.2, 1.1)
PCB70_76	2.9 (-0.2, 6.1)	**0.6 (0.0, 1.2)**	0.6 (-0.7, 1.8)
PCB66_80	**4.1 (1.0, 7.4)** *	**1.0 (0.4, 1.5)** **	0.9 (-0.4, 2.1)
PCB93_95	**4.5 (1.8, 7.3)** **	**1.3 (0.8, 1.8)** ***	-0.1 (-1.2, 0.9)

Continued Table S8. Associations of dietary patterns with each chemical and chemical class while including clinical centers as random effect intercept among the NICHD Fetal Growth Study–Singletons cohort. Generalized Linear Mixed Models (GLMM) was used with clinical centers included as a random effect intercept. Estimations with raw p-value< 0.05 were bolded. To account for multiple comparisons, Benjamini-Hochberg (BH) adjusted p-values were calculated with p< 0.001, <0.01 and <0.05 marked as ***, **, and * respectively. All p-values were two-sided. All models were adjusted for total lipids (except for PFASs and metals), maternal race/ethnicity, age, physical activity level, pre-pregnancy BMI, education level, income, parity, tobacco exposure, and total energy intake; All chemicals were log-transformed and scaled. change% [(exp(beta) − 1) × 100] was reported to benefit interpretation.

Chemicals	aMED [Change% (95% CI)]	aHEI [Change% (95% CI)]	DASH [Change% (95% CI)]
PCB90_101_89	**4.3 (1.7, 7.0)** **	**1.3 (0.8, 1.7)** ***	-0.5 (-1.5, 0.5)
PCB99	1.0 (-2.0, 4.0)	**0.6 (0.0, 1.2)**	-0.5 (-1.6, 0.7)
PCB110	2.9 (-0.1, 6.1)	**1.1 (0.5, 1.6)** ***	0.6 (-0.6, 1.8)
PCB118_106	**4.5 (1.7, 7.4)** **	**1.3 (0.7, 1.8)** ***	0.7 (-0.4, 1.8)
PCB114_122	1.7 (-1.3, 4.8)	**0.7 (0.2, 1.3)** *	-0.3 (-1.5, 0.9)
PCB105_127	**4.5 (1.5, 7.5)** *	**1.1 (0.6, 1.7)** ***	0.1 (-1.1, 1.2)
PCB146_161	1.9 (-1.1, 5.0)	**0.8 (0.2, 1.3)** *	0.2 (-1.0, 1.4)
PCB153	**4.3 (1.2, 7.5)** *	**1.2 (0.7, 1.8)** ***	0.7 (-0.6, 1.9)
PCB137	**4.3 (1.5, 7.1)** *	**1.2 (0.7, 1.7)** ***	0.4 (-0.6, 1.5)
PCB138_158	**3.9 (0.9, 6.9)** *	**1.1 (0.5, 1.6)** ***	0.4 (-0.7, 1.6)
PCB128	**5.2 (2.3, 8.2)** **	**1.5 (0.9, 2.0)** ***	0.3 (-0.8, 1.4)
PCB167	**5.4 (2.3, 8.6)** **	**1.3 (0.7, 1.9)** ***	0.8 (-0.4, 2.0)
PCB156	**5.9 (3.3, 8.7)** ***	**1.2 (0.8, 1.7)** ***	1.0 (-0.1, 2.0)
PCB157	**4.6 (1.5, 7.8)** *	**1.0 (0.4, 1.6)** **	1.1 (-0.1, 2.4)
PCB182_187	**3.4 (0.3, 6.5)**	**0.8 (0.2, 1.4)** *	1.0 (-0.3, 2.2)
PCB183	**4.9 (2.1, 7.9)** **	**0.9 (0.4, 1.4)** **	0.7 (-0.5, 1.8)
PCB177	**4.4 (1.4, 7.4)** *	**1.1 (0.6, 1.7)** ***	0.6 (-0.6, 1.7)
PCB172_192	**6.0 (3.2, 8.9)** ***	**1.4 (0.9, 1.9)** ***	0.9 (-0.2, 2.0)
PCB180	**6.4 (3.5, 9.3)** ***	**1.2 (0.7, 1.7)** ***	**1.6 (0.5, 2.8)**
PCB170	**5.8 (3.2, 8.5)** ***	**1.2 (0.7, 1.6)** ***	**1.0 (0.0, 2.1)**
PCB202	**5.3 (2.2, 8.5)** **	**1.1 (0.5, 1.7)** ***	0.7 (-0.5, 1.9)
PCB199	**4.4 (1.3, 7.6)** *	**1.1 (0.5, 1.7)** ***	0.8 (-0.4, 2.0)
PCB196_203	**5.5 (2.4, 8.7)** **	**1.1 (0.6, 1.7)** ***	**1.2 (0.0, 2.4)**
PCB195	**5.0 (2.0, 8.2)** **	**1.0 (0.4, 1.5)** **	0.7 (-0.5, 1.9)
PCB194	**6.2 (3.3, 9.2)** ***	**1.2 (0.7, 1.7)** ***	0.9 (-0.2, 2.0)
PCB208	2.7 (-0.4, 5.9)	0.5 (-0.1, 1.1)	0.9 (-0.3, 2.2)
PCB206	**3.7 (0.7, 6.9)**	**1.0 (0.4, 1.6)** **	**1.3 (0.1, 2.5)**
PCB209	**3.2 (0.2, 6.4)**	**0.9 (0.3, 1.4)** **	1.2 (-0.0, 2.4)
Total PCBs	**5.3 (2.6, 8.1)** **	**1.3 (0.8, 1.8)** ***	0.7 (-0.4, 1.8)
NMeFOSAA	-2.8 (-5.7, 0.2)	-0.6 (-1.1, 0.0)	**-1.8 (-3.0, -0.6)**
PFDS	**3.0 (0.3, 5.8)**	**0.8 (0.3, 1.3)** **	-0.4 (-1.5, 0.7)
PFDoDA	-1.1 (-3.5, 1.4)	0.3 (-0.2, 0.8)	-0.7 (-1.7, 0.3)
PFHpA	2.2 (-0.4, 4.9)	0.4 (-0.1, 0.9)	0.0 (-1.0, 1.1)
PFHxS	-0.1 (-2.8, 2.7)	0.4 (-0.1, 0.9)	**-2.0 (-3.1, -1.0)**
PFOS	1.9 (-0.7, 4.5)	**0.8 (0.3, 1.2)** **	**1.1 (0.1, 2.1)**
PFOA	2.1 (-0.9, 5.2)	**0.9 (0.3, 1.4)** **	1.2 (-0.0, 2.4)

Continued Table S8. Associations of dietary patterns with each chemical and chemical class while including clinical centers as random effect intercept among the NICHD Fetal Growth Study–Singletons cohort. Generalized Linear Mixed Models (GLMM) was used with clinical centers included as a random effect intercept. Estimations with raw *p*-value< 0.05 were bolded. To account for multiple comparisons, Benjamini-Hochberg (BH) adjusted *p*-values were calculated with *p*< 0.001, <0.01 and <0.05 marked as ***, **, and * respectively. All *p*-values were two-sided. All models were adjusted for total lipids (except for PFASs and metals), maternal race/ethnicity, age, physical activity level, pre-pregnancy BMI, education level, income, parity, tobacco exposure, and total energy intake; All chemicals were log-transformed and scaled. change% [(exp(beta) − 1) × 100] was reported to benefit interpretation.

Chemicals	aMED [Change% (95% CI)]	aHEI [Change% (95% CI)]	DASH [Change% (95% CI)]
PFNA	**6.2 (3.5, 9.0)** ***	**2.0 (1.5, 2.4)** ***	-0.1 (-1.2, 0.9)
PFDA	**3.1 (0.4, 5.9)**	0.3 (-0.2, 0.9)	**1.5 (0.5, 2.6)**
PFUnDA	2.0 (-0.7, 4.7)	**0.9 (0.4, 1.4)** **	**1.6 (0.6, 2.7)**
Total PFASs	0.5 (-2.3, 3.5)	**0.6 (0.1, 1.2)** *	**-1.5 (-2.6, -0.4)**
As	1.8 (-1.4, 5.1)	**0.8 (0.2, 1.4)** *	-0.5 (-1.7, 0.8)
Ba	-0.3 (-3.5, 3.0)	-0.3 (-0.9, 0.3)	-0.2 (-1.6, 1.1)
Cd	0.8 (-2.5, 4.3)	0.3 (-0.4, 0.9)	1.4 (-0.0, 2.7)
Co	1.1 (-2.3, 4.5)	-0.2 (-0.8, 0.5)	0.4 (-0.9, 1.8)
Cr	**3.7 (0.2, 7.3)**	0.5 (-0.2, 1.1)	0.5 (-0.8, 1.9)
Cs	2.8 (-0.2, 6.0)	**1.3 (0.7, 1.9)** ***	**1.4 (0.2, 2.6)**
Cu	-2.2 (-5.3, 1.0)	-0.5 (-1.1, 0.1)	0.0 (-1.2, 1.3)
Hg	2.1 (-1.0, 5.4)	**1.3 (0.7, 1.9)** ***	0.1 (-1.1, 1.4)
Mn	-2.2 (-5.5, 1.2)	0.0 (-0.6, 0.7)	-0.8 (-2.2, 0.5)
Mo	2.5 (-0.9, 6.0)	0.2 (-0.4, 0.9)	1.2 (-0.2, 2.6)
Pb	**3.3 (0.2, 6.5)**	**0.7 (0.1, 1.2)** *	0.7 (-0.5, 1.9)
Sb	-0.5 (-3.7, 2.7)	0.1 (-0.5, 0.7)	0.7 (-0.6, 2.0)
Se	-2.0 (-5.2, 1.3)	**-1.1 (-1.7, -0.5)** **	-0.9 (-2.2, 0.4)
Sn	0.1 (-3.3, 3.6)	-0.4 (-1.0, 0.2)	-0.7 (-2.1, 0.7)
Tl	1.1 (-2.2, 4.6)	0.5 (-0.1, 1.1)	1.1 (-0.3, 2.4)
Zn	**-3.8 (-6.9, -0.6)**	-0.4 (-1.0, 0.3)	-1.1 (-2.4, 0.2)
Total metals	**-3.8 (-7.0, -0.6)**	-0.6 (-1.3, 0.0)	-0.7 (-2.0, 0.7)

Table S9. Imputation analysis for associations of dietary patterns with each chemical among the NICHD Fetal Growth Study–Singletons cohort. Chemical concentration values below the LOD were imputed to assess the result robustness. Estimations with raw p-value< 0.05 were bolded. To account for multiple comparisons, Benjamini-Hochberg (BH) adjusted p-values were calculated with p< 0.001, <0.01 and <0.05 marked as ***, **, and * respectively. All p-values were two-sided. All models were adjusted for maternal race/ethnicity, age, physical activity, maternal race/ethnicity, maternal BMI, maternal educational level, household income level, parity, tobacco exposure, and total energy intake. Chemicals (except for PFASs and metals) were standardized by total lipids, and all chemicals were further log-transformed and scaled. change% [(exp(beta) − 1) × 100] was reported to benefit interpretation.

Chemicals	aMED [Change% (95% CI)]	aHEI [Change% (95% CI)]	DASH [Change% (95% CI)]
BetaHCH	1.5 (-1.4, 4.5)	**0.6 (0.1, 1.2)** *	1.0 (-0.1, 2.1)
GammaHCH	-0.6 (-4.7, 3.7)	-0.2 (-0.9, 0.5)	-0.3 (-1.8, 1.2)
HCB	1.0 (-1.5, 3.6)	0.4 (-0.1, 0.9)	0.8 (-0.2, 1.9)
Oxychlordane	1.6 (-2.2, 5.6)	0.2 (-0.4, 0.9)	-0.4 (-1.9, 1.1)
TransChlordane	0.4 (-2.7, 3.6)	-0.3 (-0.9, 0.3)	-0.7 (-1.9, 0.5)
TransNo_chlor	-1.2 (-4.1, 1.7)	-0.4 (-1.0, 0.1)	-1.1 (-2.2, 0.1)
P_P_DDE	-0.3 (-3.6, 3.1)	-0.1 (-0.8, 0.7)	0.5 (-1.3, 2.3)
O_P_DDD	-2.4 (-5.3, 0.6)	-0.4 (-0.9, 0.2)	-1.0 (-2.3, 0.2)
P_P_DDD	-0.5 (-4.1, 3.1)	-0.2 (-1.0, 0.6)	-0.5 (-2.1, 1.1)
P_P_DDT	**3.5 (0.5, 6.6)**	**1.0 (0.4, 1.5)** **	0.4 (-0.8, 1.6)
Mirex	1.3 (-2.0, 4.6)	0.5 (-0.1, 1.1)	0.5 (-0.8, 1.9)
BDE28	0.0 (-3.6, 3.8)	-0.3 (-0.9, 0.4)	0.3 (-1.1, 1.7)
BDE47	-2.0 (-4.8, 0.9)	-0.4 (-1.0, 0.1)	-0.9 (-2.0, 0.3)
BDE100	-2.0 (-4.8, 0.9)	-0.4 (-0.9, 0.2)	-0.9 (-2.1, 0.3)
BDE99	-2.1 (-5.1, 1.0)	-0.1 (-0.7, 0.5)	-1.1 (-2.3, 0.1)
BDE85	0.4 (-3.6, 4.7)	0.0 (-0.9, 0.9)	0.0 (-2.1, 2.2)
PBB153	0.7 (-2.7, 4.3)	0.1 (-0.5, 0.8)	0.1 (-1.3, 1.5)
BDE154	-0.7 (-3.8, 2.6)	-0.0 (-0.6, 0.6)	-0.1 (-1.2, 1.0)
BDE153	-1.4 (-4.5, 1.8)	-0.3 (-0.9, 0.3)	-0.2 (-1.5, 1.2)
BDE183	0.5 (-2.7, 3.8)	0.1 (-0.5, 0.6)	0.5 (-0.6, 1.5)
PCB5_8	2.2 (-1.0, 5.6)	0.7 (-0.0, 1.3)	0.8 (-0.6, 2.2)
PCB18_17	1.1 (-1.9, 4.2)	-0.0 (-0.6, 0.6)	0.1 (-1.1, 1.4)
PCB31_28	1.1 (-2.0, 4.3)	0.2 (-0.4, 0.8)	-0.0 (-1.3, 1.3)
PCB33_20	1.4 (-1.9, 4.7)	0.5 (-0.0, 1.1)	0.5 (-0.7, 1.8)
PCB22	1.5 (-1.8, 4.9)	0.3 (-0.3, 0.9)	0.2 (-1.1, 1.5)
PCB52_73	**3.6 (0.6, 6.6)**	**0.9 (0.3, 1.4)** **	0.3 (-0.9, 1.5)
PCB49_43	3.0 (-0.1, 6.2)	**1.1 (0.5, 1.6)** **	1.1 (-0.1, 2.3)
PCB47_48_75	1.3 (-1.6, 4.3)	0.1 (-0.5, 0.6)	0.0 (-1.1, 1.2)
PCB44	1.9 (-1.7, 5.7)	0.4 (-0.3, 1.0)	0.5 (-1.0, 1.9)
PCB41_64	0.2 (-2.9, 3.5)	0.2 (-0.4, 0.8)	-0.1 (-1.3, 1.2)
PCB74_61	**4.2 (1.2, 7.4)** *	**1.1 (0.5, 1.7)** ***	0.2 (-1.0, 1.4)
PCB70_76	1.9 (-1.3, 5.2)	0.3 (-0.4, 1.0)	0.2 (-1.3, 1.7)
PCB66_80	**3.6 (0.3, 6.9)**	**0.8 (0.1, 1.4)**	0.5 (-0.8, 1.8)
PCB93_95	**4.7 (1.4, 8.1)** *	**1.4 (0.9, 2.0)** ***	0.2 (-1.0, 1.5)
PCB90_101_89	**4.6 (1.8, 7.6)** *	**1.3 (0.8, 1.8)** ***	-0.3 (-1.5, 0.8)
PCB99	1.0 (-2.6, 4.7)	0.6 (-0.0, 1.2)	-0.9 (-2.2, 0.5)

Continued Table S9. Imputation analysis for associations of dietary patterns with each chemical among the NICHD Fetal Growth Study–Singletons cohort. Chemical concentration values below the LOD were imputed to assess the result robustness. Estimations with raw p-value< 0.05 were bolded. To account for multiple comparisons, Benjamini-Hochberg (BH) adjusted p-values were calculated with p< 0.001, <0.01 and <0.05 marked as ***, **, and * respectively. All p-values were two-sided. All models were adjusted for maternal race/ethnicity, age, physical activity, maternal race/ethnicity, maternal BMI, maternal educational level, household income level, parity, tobacco exposure, and total energy intake. Chemicals (except for PFASs and metals) were standardized by total lipids, and all chemicals were further log-transformed and scaled. change% [(exp(beta) − 1) × 100] was reported to benefit interpretation.

Chemicals	aMED [Change% (95% CI)]	aHEI [Change% (95% CI)]	DASH [Change% (95% CI)]
PCB110	2.3 (-1.2, 5.9)	**1.0 (0.3, 1.7)** *	0.6 (-1.0, 2.3)
PCB118_106	**4.6 (1.8, 7.4)** *	**1.3 (0.8, 1.9)** ***	0.8 (-0.3, 1.9)
PCB114_122	2.3 (-0.8, 5.5)	**0.6 (0.1, 1.2)**	-0.2 (-1.4, 1.0)
PCB105_127	**4.2 (1.2, 7.4)** *	**1.1 (0.6, 1.7)** ***	0.1 (-1.1, 1.3)
PCB146_161	0.3 (-3.8, 4.6)	0.4 (-0.3, 1.2)	-0.4 (-1.9, 1.1)
PCB153	**3.3 (0.2, 6.5)**	**1.0 (0.4, 1.6)** **	0.6 (-0.7, 1.9)
PCB137	**4.3 (1.6, 7.1)** *	**1.3 (0.7, 1.8)** ***	0.5 (-0.6, 1.6)
PCB138_158	**3.0 (0.2, 5.9)**	**0.8 (0.3, 1.4)** **	0.4 (-0.7, 1.5)
PCB128	**4.5 (1.7, 7.4)** *	**1.2 (0.7, 1.8)** ***	0.1 (-1.0, 1.3)
PCB167	**4.3 (1.2, 7.6)** *	**0.9 (0.3, 1.5)** *	0.4 (-0.8, 1.7)
PCB156	**5.8 (3.1, 8.7)** ***	**1.3 (0.8, 1.8)** ***	**1.1 (0.0, 2.1)**
PCB157	**3.7 (0.6, 7.0)**	**0.7 (0.1, 1.2)**	0.7 (-0.5, 1.9)
PCB182_187	**4.1 (0.4, 8.0)**	**0.8 (0.1, 1.6)**	1.0 (-0.3, 2.3)
PCB183	**4.7 (1.6, 8.0)** *	**0.8 (0.2, 1.4)** *	0.5 (-0.6, 1.7)
PCB177	**4.9 (1.6, 8.3)** *	**1.2 (0.6, 1.8)** ***	0.7 (-0.4, 2.0)
PCB172_192	**5.5 (2.7, 8.5)** **	**1.4 (0.9, 1.9)** ***	0.9 (-0.2, 2.0)
PCB180	**6.0 (3.0, 9.0)** **	**1.1 (0.5, 1.6)** **	**1.6 (0.3, 2.8)**
PCB170	**5.8 (3.3, 8.5)** ***	**1.2 (0.8, 1.7)** ***	**1.1 (0.1, 2.1)**
PCB202	**4.0 (0.8, 7.3)**	**0.9 (0.3, 1.5)** **	0.8 (-0.5, 2.0)
PCB199	**3.9 (0.6, 7.3)**	**0.8 (0.2, 1.4)** *	0.8 (-0.5, 2.2)
PCB196_203	**4.4 (0.9, 8.0)**	**0.9 (0.3, 1.5)** **	0.8 (-0.5, 2.1)
PCB195	**4.0 (0.6, 7.5)**	**0.7 (0.1, 1.2)**	0.3 (-0.9, 1.6)
PCB194	**5.7 (2.4, 9.1)** *	**1.1 (0.5, 1.7)** **	0.7 (-0.8, 2.2)
PCB208	2.0 (-1.2, 5.3)	0.2 (-0.4, 0.8)	0.3 (-0.9, 1.6)
PCB206	3.1 (-0.4, 6.8)	**0.7 (0.0, 1.4)**	1.1 (-0.3, 2.5)
PCB209	1.1 (-2.0, 4.3)	0.2 (-0.3, 0.8)	0.4 (-0.8, 1.7)
NMeFOSAA	**-4.4 (-7.3, -1.4)** *	**-0.9 (-1.4, -0.3)** **	**-2.2 (-3.3, -1.0)** *
PFDS	**3.1 (0.2, 6.1)**	**0.9 (0.3, 1.4)** **	-0.3 (-1.4, 0.9)
PFDoDA	-1.7 (-4.3, 1.0)	0.1 (-0.4, 0.6)	-1.0 (-2.1, 0.0)
PFHpA	1.7 (-1.1, 4.5)	0.4 (-0.1, 0.9)	0.0 (-1.1, 1.1)
PFHxS	0.5 (-2.3, 3.4)	0.4 (-0.1, 0.9)	**-2.0 (-3.1, -0.9)** *
PFOS	2.3 (-0.3, 5.0)	**0.9 (0.4, 1.4)** **	**1.3 (0.2, 2.3)**
PFOA	2.5 (-0.9, 6.0)	**0.7 (0.1, 1.3)**	1.2 (-0.1, 2.6)
PFNA	**6.8 (3.9, 9.8)** ***	**2.1 (1.6, 2.7)** ***	0.1 (-1.0, 1.3)
PFDA	2.0 (-1.3, 5.3)	0.1 (-0.5, 0.7)	0.9 (-0.3, 2.2)
PFUnDA	2.1 (-0.8, 5.0)	**0.9 (0.4, 1.4)** **	**1.7 (0.5, 2.8)**

Continued Table S9. Imputation analysis for associations of dietary patterns with each chemical among the NICHD Fetal Growth Study–Singletons cohort. Chemical concentration values below the LOD were imputed to assess the result robustness. Estimations with raw *p*-value< 0.05 were bolded. To account for multiple comparisons, Benjamini-Hochberg (BH) adjusted *p*-values were calculated with *p*< 0.001, <0.01 and <0.05 marked as ***, **, and * respectively. All *p*-values were two-sided. All models were adjusted for maternal race/ethnicity, age, physical activity, maternal race/ethnicity, maternal BMI, maternal educational level, household income level, parity, tobacco exposure, and total energy intake. Chemicals (except for PFASs and metals) were standardized by total lipids, and all chemicals were further log-transformed and scaled. change% [(exp(beta) − 1) × 100] was reported to benefit interpretation.

Chemicals	aMED [Change% (95% CI)]	aHEI [Change% (95% CI)]	DASH [Change% (95% CI)]
As	2.6 (-0.8, 6.3)	**1.0 (0.4, 1.7)** *	-0.2 (-1.5, 1.1)
Ba	-0.1 (-3.4, 3.3)	-0.5 (-1.1, 0.2)	-0.7 (-2.1, 0.6)
Cd	1.0 (-2.4, 4.5)	0.3 (-0.3, 1.0)	1.3 (-0.0, 2.7)
Co	1.0 (-2.3, 4.5)	-0.1 (-0.8, 0.5)	0.4 (-0.9, 1.8)
Cr	3.2 (-0.8, 7.5)	0.4 (-0.3, 1.0)	0.6 (-0.8, 2.1)
Cs	2.8 (-0.3, 6.0)	**1.2 (0.6, 1.7)** ***	**1.3 (0.1, 2.6)**
Cu	-2.1 (-5.1, 1.1)	-0.5 (-1.0, 0.1)	-0.0 (-1.3, 1.3)
Hg	2.8 (-0.4, 6.1)	**1.4 (0.8, 2.0)** ***	0.4 (-0.9, 1.7)
Mn	-1.9 (-6.0, 2.4)	0.0 (-0.6, 0.7)	-0.7 (-2.3, 0.8)
Mo	0.8 (-2.5, 4.2)	0.4 (-0.2, 1.0)	0.5 (-0.9, 1.8)
Pb	2.5 (-0.7, 5.8)	0.4 (-0.2, 0.9)	0.4 (-0.9, 1.6)
Sb	-1.0 (-4.3, 2.4)	-0.0 (-0.6, 0.6)	0.5 (-0.9, 1.8)
Se	-1.9 (-5.0, 1.4)	**-1.1 (-1.7, -0.5)** **	-0.9 (-2.2, 0.4)
Sn	-0.2 (-3.5, 3.2)	-0.4 (-1.0, 0.2)	-0.7 (-2.0, 0.7)
Tl	1.1 (-2.3, 4.6)	0.5 (-0.1, 1.2)	1.0 (-0.3, 2.4)
Zn	**-4.6 (-7.7, -1.4)** *	**-0.7 (-1.3, -0.0)**	**-1.7 (-3.0, -0.4)**

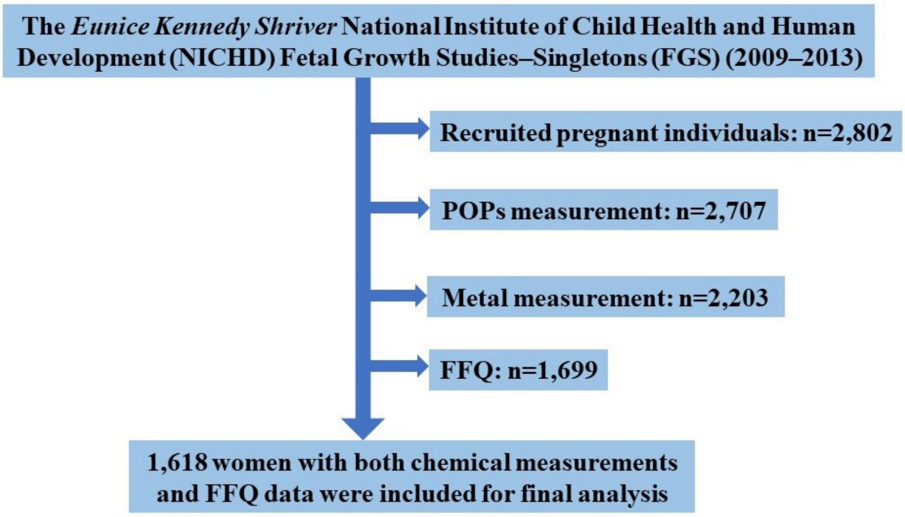

Fig S1| Flowchart of the study analytical population.

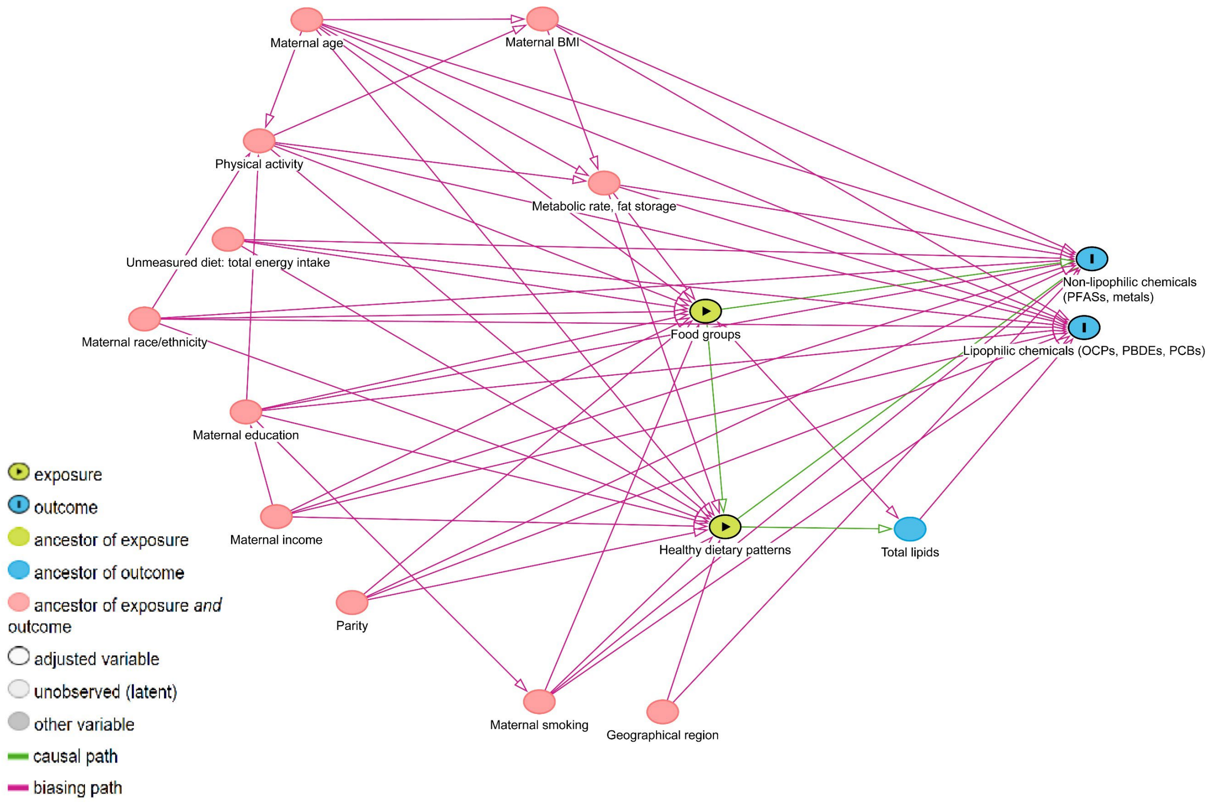

Fig S2| Directed acyclic graph for covariate selection.

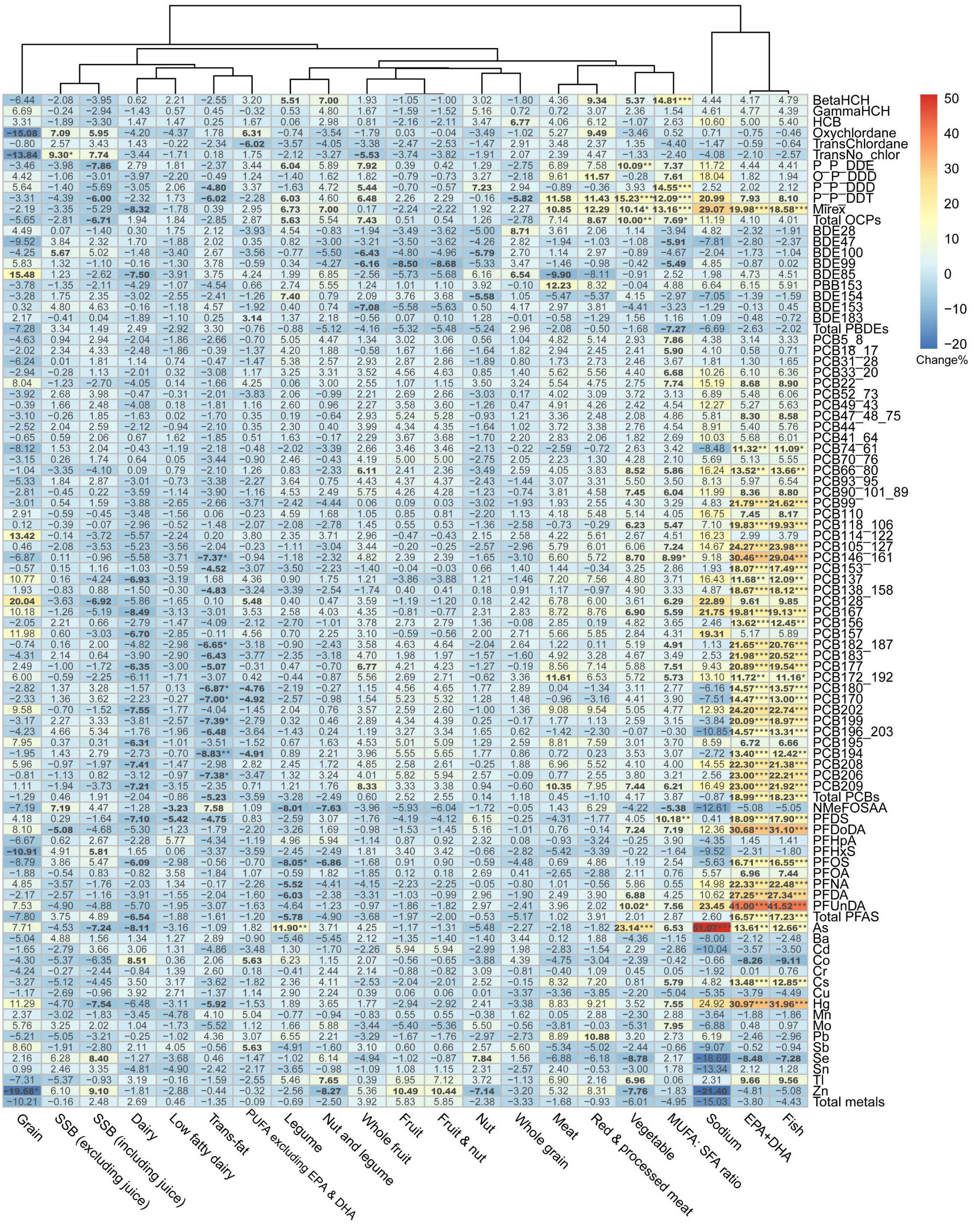

Fig S3| Associations of each food group consumption with each chemical and chemical class using multiple linear regression models among the NICHD Fetal Growth Study–Singletons cohort. Change% was presented in heatmap and cluster analysis was applied. Considering the multiple comparisons among associations between each food group with each chemical, the significant level of two-sided *p*-value was adjusted by the Benjamini-Hochberg procedure. Adjusted *p*-values were presented in the heatmap combined with cluster analysis. Estimations with two-sided and raw *p*-values< 0.05 were bolded, and among them Benjamini-Hochberg (BH) adjusted *p*-values< 0.001, <0.01 and <0.05 were marked as ***, **, and * respectively.

All models were adjusted for maternal race/ethnicity, age, physical activity, maternal race/ethnicity, maternal BMI, maternal educational level, household income level, parity, tobacco exposure, and total energy intake. All chemicals (except for PFASs and metals) were standardized by total lipids, and further log-transformed and scaled. SSB: sugar sweetened beverage. MUFA: monounsaturated fatty acids. SFA: saturated fatty acids. PUFA: polyunsaturated fatty acid.

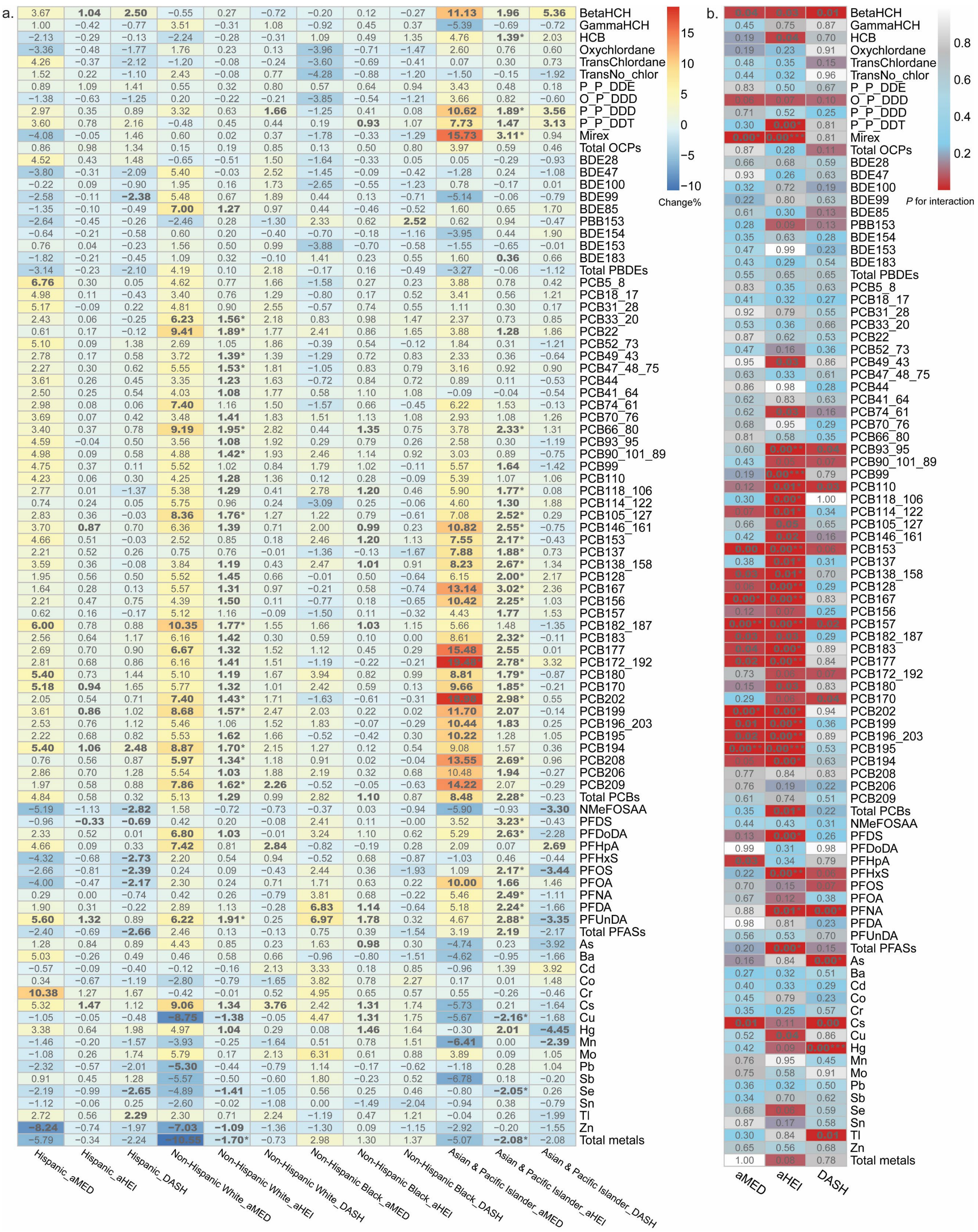

Fig S4| Associations of different dietary patterns with chemicals, stratified by race/ethnicity among the NICHD Fetal Growth Study–Singletons cohort. a, Change% [(exp(beta) − 1) × 100] was reported with different colors to benefit interpretation: the redder the grid cell, the higher the change%, while the bluer, the lower the change%. When change% is associated with raw *p*-value< 0.05, the numbers are shown in black. To account for multiple comparisons, change% with adjusted *p*-value< 0.05, < 0.01, < 0.001 are marked as *, **, ***, respectively. b, Interaction test was examined between Asian & Pacific Islander and other races/ethnicities, with *p*-value< 0.05 shown in black number and adjusted *p*-value< 0.05, < 0.01, < 0.001 marked as *, **, ***,

respectively. All p-values were two-sided. All models were adjusted for maternal age, physical activity, maternal BMI, maternal educational level, household income level, parity, tobacco exposure, and total energy intake. Chemicals (except for PFASs and metals) were standardized by total lipids, and all chemicals were further log-transformed and scaled.

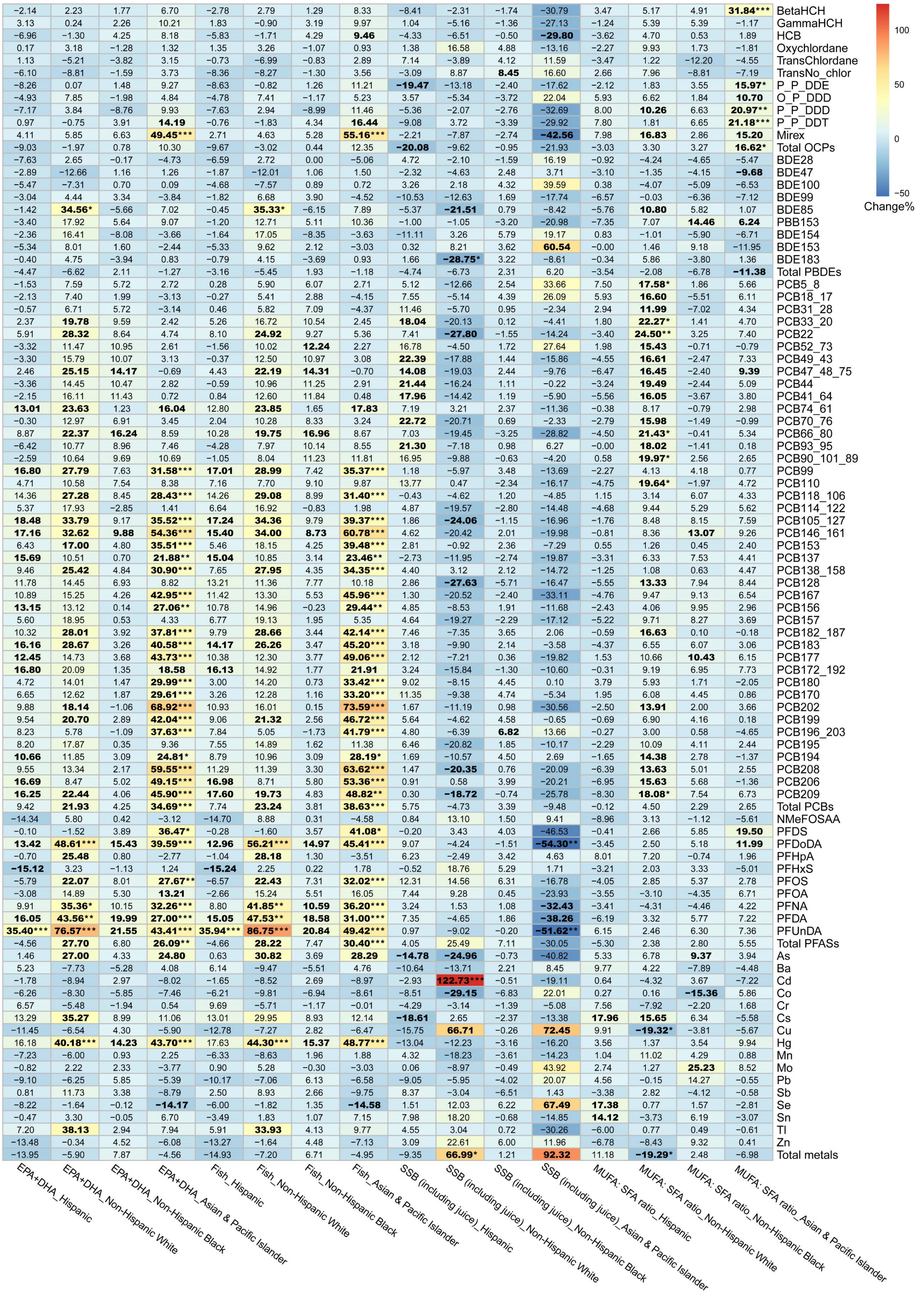

Fig S5| Associations of different food groups with chemicals, stratified by race/ethnicity among the NICHD Fetal Growth Study–Singletons cohort. Only results of food groups that showed obvious differences

among races/ethnicities were listed due to the limited page space. Estimations with two-sided and raw *p*-values< 0.05 were bolded. To account for multiple comparisons, Benjamini-Hochberg (BH) adjusted *p*-values were calculated with *p*-value< 0.001, <0.01 and <0.05 marked as ***, **, and * respectively. All models were adjusted for maternal age, physical activity, maternal race/ethnicity, maternal BMI, maternal educational level, household income level, parity, tobacco exposure, and total energy intake. Chemicals (except for PFASs and metals) were standardized by total lipids, and all chemicals were further log-transformed and scaled. SSB: sugar sweetened beverage. MUFA: monounsaturated fatty acids. SFA: saturated fatty acids. PUFA: polyunsaturated fatty acid.

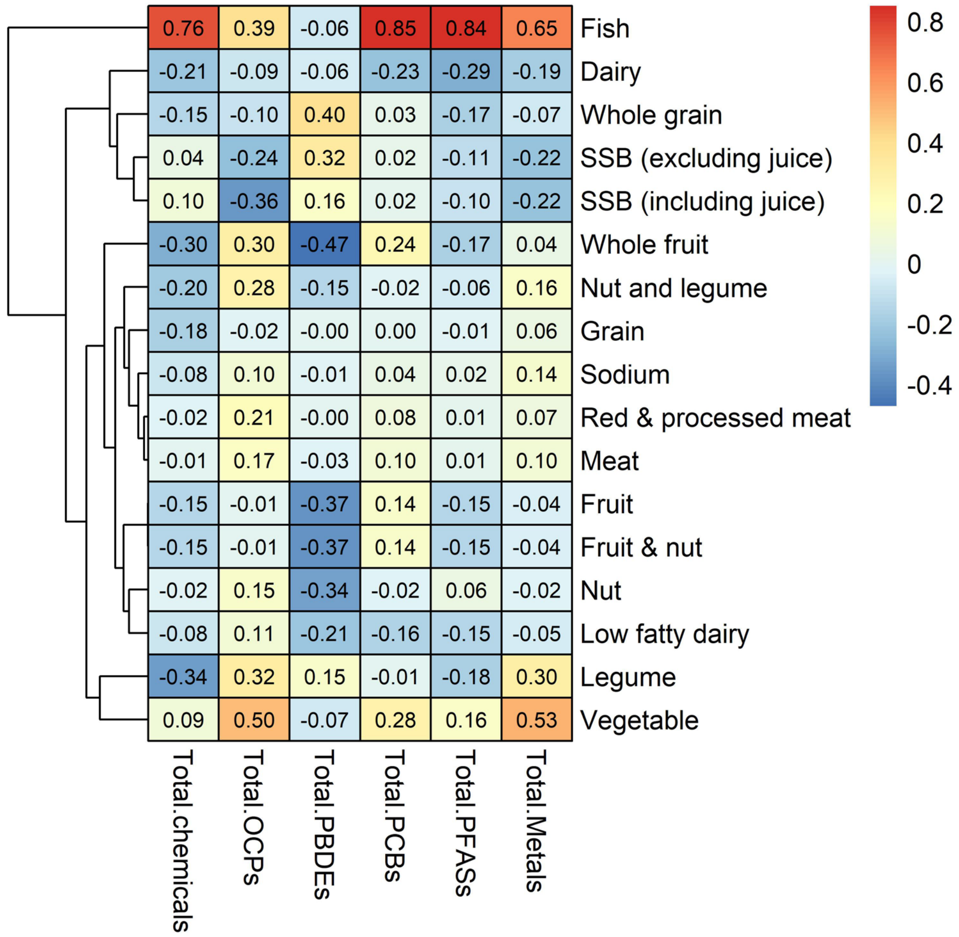

Fig S6| Loading effect of different food groups on chemical classes after excluding food-group derived nutrients among the NICHD Fetal Growth Study–Singletons cohort. Loading effect of different food groups on chemical classes were derived from RRR while adjusting for residuals of the relevant confounders, including maternal age, physical activity, maternal race/ethnicity, maternal BMI, maternal educational level, household income level, parity, tobacco exposure, and total energy intake. SSB: sugar-sweetened beverage. EPA+DHA, MUFA: SFA ratio, and PUFA excluding EPA & DHA derived from different food groups were not included.

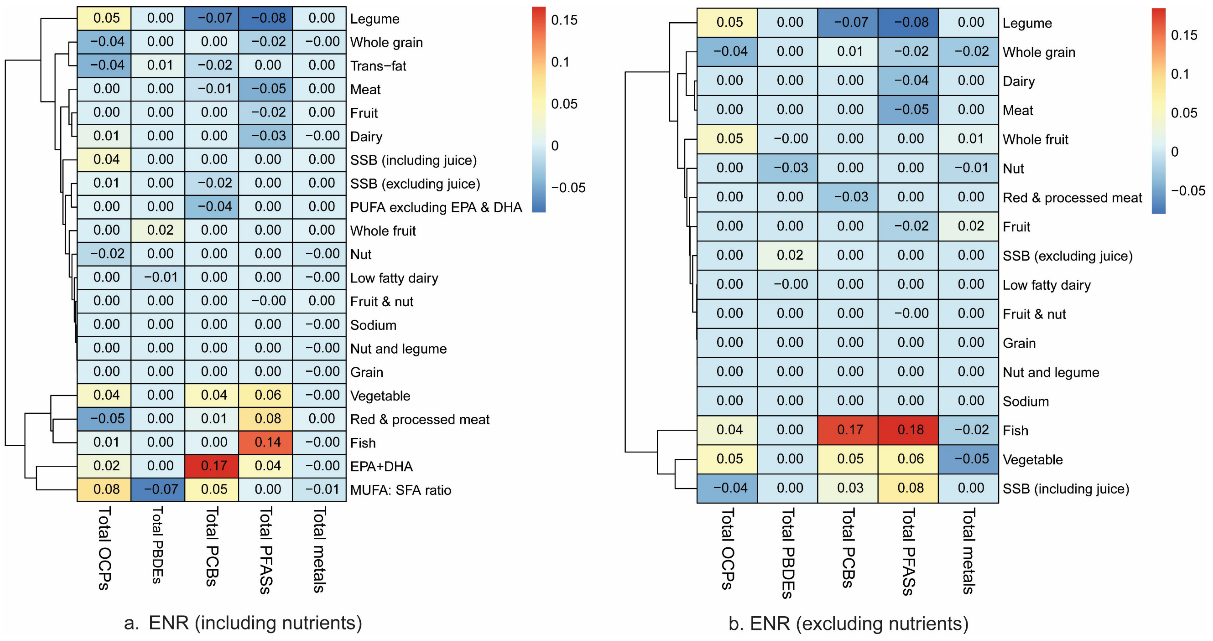

Fig S7| Elastic net regression models for the associations between food groups and different chemical classes (β) among the NICHD Fetal Growth Study–Singletons cohort. Coefficients (β) of each food group were visualized to indicate the strength and direction of the relationship between food groups and the chemical classes. All models were adjusted for maternal age, physical activity, maternal race/ethnicity, maternal BMI, maternal educational level, household income level, parity, tobacco exposure, and total energy intake. a, all food groups (including nutrients) were included. b, food groups excluding EPA+DHA, PUFA excluding EPA & DHA, Trans-fat, and MUFA: SFA ratio were included. SSB: sugar-sweetened beverage. MUFA: monounsaturated fatty acids. SFA: saturated fatty acids. PUFA: polyunsaturated fatty acid.

References

1. Grewal, J., *et al.* Cohort Profile: NICHD Fetal Growth Studies-Singletons and Twins. *International journal of epidemiology* **47**, 25-25l (2018).
2. Li, M., *et al.* Healthy dietary patterns and common pregnancy complications: a prospective and longitudinal study. *The American journal of clinical nutrition* **114**, 1229-1237 (2021).
3. Subar, A.F., *et al.* Comparative validation of the Block, Willett, and National Cancer Institute food frequency questionnaires : the Eating at America's Table Study. *American journal of epidemiology* **154**, 1089-1099 (2001).
4. Chiuve, S.E., *et al.* Alternative dietary indices both strongly predict risk of chronic disease. *The Journal of nutrition* **142**, 1009-1018 (2012).
5. Trichopoulou, A., Costacou, T., Bamia, C. & Trichopoulos, D. Adherence to a Mediterranean diet and survival in a Greek population. *N Engl J Med* **348**, 2599-2608 (2003).
6. McCullough, M.L., *et al.* Adherence to the Dietary Guidelines for Americans and risk of major chronic disease in women. *The American journal of clinical nutrition* **72**, 1214-1222 (2000).
7. Buck Louis, G.M., *et al.* Endocrine disruptors and neonatal anthropometry, NICHD Fetal Growth Studies - Singletons. *Environment international* **119**, 515-526 (2018).
8. Ma, W.L., *et al.* Analysis of polychlorinated biphenyls and organochlorine pesticides in archived dried blood spots and its application to track temporal trends of environmental chemicals in newborns. *Environ Res* **133**, 204-210 (2014).
9. Zheng, Y., *et al*. A Prospective Study of Early Pregnancy Essential Metal(loid)s and Glucose Levels Late in the Second Trimester. *The Journal of clinical endocrinology and metabolism* 104, 4295-4303 (2019).
10. Rahman, M.L., *et al.* Persistent organic pollutants and gestational diabetes: A multi-center prospective cohort study of healthy US women. *Environment international* **124**, 249-258 (2019).
11. Xiao, X., *et al.* Spatial distribution of benthic toxicity and sediment-bound metals and arsenic in Guangzhou urban waterways: Influence of land use. *Journal of hazardous materials* **439**, 129634 (2022).
12. Schisterman, E.F., Whitcomb, B.W., Louis, G.M. & Louis, T.A. Lipid adjustment in the analysis of environmental contaminants and human health risks. *Environ Health Perspect* **113**, 853-857 (2005).
13. Akins, J.R., Waldrep, K. & Bernert, J.T., Jr. The estimation of total serum lipids by a completely enzymatic 'summation' method. *Clinica chimica acta; international journal of clinical chemistry* **184**, 219-226 (1989).
14. Phillips, D.L., *et al.* Chlorinated hydrocarbon levels in human serum: effects of fasting and feeding. *Archives of environmental contamination and toxicology* **18**, 495-500 (1989).
15. Qian, J., *et al.* LARGE-SCALE MULTIVARIATE SPARSE REGRESSION WITH APPLICATIONS TO UK BIOBANK. *Ann Appl Stat* **16**, 1891-1918 (2022).

16. Carwile, J.L., *et al.* Dietary correlates of urinary phthalate metabolite concentrations in 6-19 Year old children and adolescents. *Environ Res* **204**, 112083 (2022).
17. O'Brien, K.M., Upson, K., Cook, N.R. & Weinberg, C.R. Environmental Chemicals in Urine and Blood: Improving Methods for Creatinine and Lipid Adjustment. *Environ Health Perspect* **124**, 220-227 (2016).
18. Faquih, T., *et al.* A Workflow for Missing Values Imputation of Untargeted Metabolomics Data. *Metabolites* **10**(2020).
19. Azur, M.J., Stuart, E.A., Frangakis, C. & Leaf, P.J. Multiple imputation by chained equations: what is it and how does it work? *Int J Methods Psychiatr Res* **20**, 40-49 (2011).
