# Supplementary material for: Healthy dietary patterns are associated with exposure to environmental chemicals in a pregnancy cohort
Source: Nat Food. 2024 Jul 1;5(7):563–8. doi: 10.1038/s43016-024-01013-x (PMC11272572; doi:10.1038/s43016-024-01013-x)
Supplement: Supplementary file 2 — Reporting Summary [file 43016_2024_1013_MOESM2_ESM.pdf]

nature portfolio

Corresponding author(s): Cuilin Zhang

Last updated by author(s): Jun 11, 2024



# Reporting Summary

Nature Portfolio wishes to improve the reproducibility of the work that we publish. This form provides structure for consistency and transparency in reporting. For further information on Nature Portfolio policies, see our Editorial Policies and the Editorial Policy Checklist.

## Statistics

For all statistical analyses, confirm that the following items are present in the figure legend, table legend, main text, or Methods section.

| n/a | Confirmed | |
|---|---|---|
| ☐ | ☒ | The exact sample size (*n*) for each experimental group/condition, given as a discrete number and unit of measurement |
| ☐ | ☒ | A statement on whether measurements were taken from distinct samples or whether the same sample was measured repeatedly |
| ☐ | ☒ | The statistical test(s) used AND whether they are one- or two-sided<br>*Only common tests should be described solely by name; describe more complex techniques in the Methods section.* |
| ☐ | ☒ | A description of all covariates tested |
| ☐ | ☒ | A description of any assumptions or corrections, such as tests of normality and adjustment for multiple comparisons |
| ☐ | ☒ | A full description of the statistical parameters including central tendency (e.g. means) or other basic estimates (e.g. regression coefficient) AND variation (e.g. standard deviation) or associated estimates of uncertainty (e.g. confidence intervals) |
| ☐ | ☒ | For null hypothesis testing, the test statistic (e.g. *F*, *t*, *r*) with confidence intervals, effect sizes, degrees of freedom and *P* value noted<br>*Give P values as exact values whenever suitable.* |
| ☒ | ☐ | For Bayesian analysis, information on the choice of priors and Markov chain Monte Carlo settings |
| ☒ | ☐ | For hierarchical and complex designs, identification of the appropriate level for tests and full reporting of outcomes |
| ☐ | ☒ | Estimates of effect sizes (e.g. Cohen's *d*, Pearson's *r*), indicating how they were calculated |

*Our web collection on statistics for biologists contains articles on many of the points above.*

## Software and code

Policy information about availability of computer code

| | |
|---|---|
| Data collection | No software was used for data collection. |
| Data analysis | SAS 9.4 and R 4.2 were used for data analysis. Open source codes and scripts used for the analyses or figures are available at the GitHub repository (https://github.com/GuoqiYu2023/Nature-Food-2024). |

For manuscripts utilizing custom algorithms or software that are central to the research but not yet described in published literature, software must be made available to editors and reviewers. We strongly encourage code deposition in a community repository (e.g. GitHub). See the Nature Portfolio guidelines for submitting code & software for further information.

## Data

Policy information about availability of data

All manuscripts must include a data availability statement. This statement should provide the following information, where applicable:

- Accession codes, unique identifiers, or web links for publicly available datasets
- A description of any restrictions on data availability
- For clinical datasets or third party data, please ensure that the statement adheres to our policy

The data used in this study are not publicly available due to privacy and confidentiality agreements. Access to the data is restricted to protect the personal and health information of the participants, in accordance with ethical guidelines and regulations. Researchers interested in accessing the data may contact the

corresponding author with a detailed request and may be required to sign a data use agreement to ensure the protection of participant confidentiality.

## Human research participants

Policy information about studies involving human research participants and Sex and Gender in Research.

| | |
|---|---|
| Reporting on sex and gender | In this study, only pregnant women were included for data analysis. Sex (biological attribute) was used to identify the population and our findings can be applied to pregnant women, and even general population. Since only pregnant women were investigated, no gender- or sex-based analysis was performed. |
| Population characteristics | We included a total of 1,618 pregnancies from 2,802 women who gave birth between 2009-2013 in our cohort. The prevalence of GDM diagnosed by a two-step diagnostic test composed of a glucose challenge test (GCT) and an oral glucose tolerance test (OGTT) during 24-28 weeks of gestation, was 3.8%. Mean age at pregnancy initiation was 28.0, 56.7% women have normal BMI. |
| Recruitment | The study is based on prospective and multi-center data originating from a non-profit organization, and the outcome of the model is based on routine pregnancy tests that are comprehensively documented in the electronic health record. All pregnant women participated in the project voluntarily. Participants was provided with a colorful ultrasound print. No cash or other compensations were provided. The prevalence of major pregnancy complications were comparable to the general population. Sensitivity analysis was applied while incorporating weight to represent the total cohort population and showed consistent findings. Thus, the risk of selection bias is limited. |
| Ethics oversight | All recruited women provided written informed consent. Institutional review board approval was obtained at the National Institutes of Health and all participating clinical sites. No compensation was provided to the participants. |

Note that full information on the approval of the study protocol must also be provided in the manuscript.

# Field-specific reporting

Please select the one below that is the best fit for your research. If you are not sure, read the appropriate sections before making your selection.

☐ Life sciences ☐ Behavioural & social sciences ☒ Ecological, evolutionary & environmental sciences

For a reference copy of the document with all sections, see nature.com/documents/nr-reporting-summary-flat.pdf

# Ecological, evolutionary & environmental sciences study design

All studies must disclose on these points even when the disclosure is negative.

| | |
|---|---|
| Study description | This study adopted a prospective cohort design. Population data was obtained by standard questionnaire and electronic medical records. Chemical concentrations of plasma were measured by GC-MS, LC-MS, and ICP-MS. |
| Research sample | A total of 1,618 pregnant women from 2,802 women were finally included. |
| Sampling strategy | No sample size calculation was preformed since sample size was determined by the number of pregnancies recruited in the cohort. Our results showed that we have strong power to detect the differences of chemcials across women with different dietary pattern scores. |
| Data collection | Population data was obtained by standard questionnaire and disease data was extracted from electronic medical records by trained professionals. Chemical concentrations of plasma were measured by GC-MS, LC-MS, and ICP-MS without any knowledge of disease status or exposure information. |
| Timing and spatial scale | Pregnancy data and biospecimens were collected from 2009 to 2013. |
| Data exclusions | Women without chemical concentrations and diet data were excluded. |
| Reproducibility | No replicated experiment was done in terms of the nature of population study. |
| Randomization | No randomization was done in terms of the nature of observational population study. |
| Blinding | Blinding doesn't apply to the study design |

Did the study involve field work? ☐ Yes ☒ No

# Reporting for specific materials, systems and methods

We require information from authors about some types of materials, experimental systems and methods used in many studies. Here, indicate whether each material, system or method listed is relevant to your study. If you are not sure if a list item applies to your research, read the appropriate section before selecting a response.

## Materials & experimental systems

| n/a | Involved in the study |
| --- | --- |
| ☒ | ☐ Antibodies |
| ☒ | ☐ Eukaryotic cell lines |
| ☒ | ☐ Palaeontology and archaeology |
| ☒ | ☐ Animals and other organisms |
| ☐ | ☒ Clinical data |
| ☒ | ☐ Dual use research of concern |

## Methods

| n/a | Involved in the study |
| --- | --- |
| ☒ | ☐ ChIP-seq |
| ☒ | ☐ Flow cytometry |
| ☒ | ☐ MRI-based neuroimaging |

## Clinical data

Policy information about clinical studies

All manuscripts should comply with the ICMJE guidelines for publication of clinical research and a completed CONSORT checklist must be included with all submissions.

| | |
| --- | --- |
| Clinical trial registration | This study is registered in the Clinical Trial Registry (NCT 00912132). |
| Study protocol | Study protocol can be found: https://pubmed-ncbi-nlm-nih-gov.libproxy1.nus.edu.sg/29025016/ |
| Data collection | Pregnant women was recruited and data was collected in 2009-2013. |
| Outcomes | Plasma concentrations of chemicals was included as main outcomes. Chemicals were measured by GC-MS, LC-MS, and ICP-MS, where appropriate. |
